# Ras-Mediated Deregulation of the Circadian Clock in Cancer

**DOI:** 10.1371/journal.pgen.1004338

**Published:** 2014-05-29

**Authors:** Angela Relógio, Philippe Thomas, Paula Medina-Pérez, Silke Reischl, Sander Bervoets, Ewa Gloc, Pamela Riemer, Shila Mang-Fatehi, Bert Maier, Reinhold Schäfer, Ulf Leser, Hanspeter Herzel, Achim Kramer, Christine Sers

**Affiliations:** 1 Institute for Theoretical Biology, Charité - Universitätsmedizin and Humboldt-Universität zu Berlin, Berlin, Germany; 2 Knowledge Management in Bioinformatics, Institute for Computer Science, Humboldt-Universität zu Berlin, Berlin, Germany; 3 Laboratory of Molecular Tumor Pathology, Charité - Universitätsmedizin Berlin, Berlin, Germany; 4 Laboratory of Chronobiology, Institute for Medical Immunology Charité - Universitätsmedizin Berlin, Berlin, Germany; 5 German Cancer Consortium (DKTK) and German Cancer Research Center (DKFZ), Heidelberg, Germany; INSERM, France

## Abstract

Circadian rhythms are essential to the temporal regulation of molecular processes in living systems and as such to life itself. Deregulation of these rhythms leads to failures in biological processes and eventually to the manifestation of pathological phenotypes including cancer. To address the questions as to what are the elicitors of a disrupted clock in cancer, we applied a systems biology approach to correlate experimental, bioinformatics and modelling data from several cell line models for colorectal and skin cancer. We found strong and weak circadian oscillators within the same type of cancer and identified a set of genes, which allows the discrimination between the two oscillator-types. Among those genes are IFNGR2, PITX2, RFWD2, PPARγ, LOXL2, Rab6 and SPARC, all involved in cancer-related pathways. Using a bioinformatics approach, we extended the core-clock network and present its interconnection to the discriminative set of genes. Interestingly, such gene signatures link the clock to oncogenic pathways like the RAS/MAPK pathway. To investigate the potential impact of the RAS/MAPK pathway - a major driver of colorectal carcinogenesis - on the circadian clock, we used a computational model which predicted that perturbation of BMAL1-mediated transcription can generate the circadian phenotypes similar to those observed in metastatic cell lines. Using an inducible RAS expression system, we show that overexpression of RAS disrupts the circadian clock and leads to an increase of the circadian period while RAS inhibition causes a shortening of period length, as predicted by our mathematical simulations. Together, our data demonstrate that perturbations induced by a single oncogene are sufficient to deregulate the mammalian circadian clock.

## Author Summary

Living systems possess an endogenous time-generating system – the circadian clock - accountable for a 24 hours oscillation in the expression of about 10% of all genes. In mammals, disruption of oscillations is associated to several diseases including cancer. In this manuscript, we address the following question: what are the elicitors of a disrupted clock in cancer? We applied a systems biology approach to correlate experimental, bioinformatics and modelling data and could thereby identify key genes which discriminate strong and weak oscillators among cancer cell lines. Most of the discriminative genes play important roles in cell cycle regulation, DNA repair, immune system and metabolism and are involved in oncogenic pathways such as the RAS/MAPK. To investigate the potential impact of the Ras oncogene in the circadian clock we generated experimental models harbouring conditionally active Ras oncogenes. We put forward a direct correlation between the perturbation of Ras oncogene and an effect in the expression of clock genes, found by means of mathematical simulations and validated experimentally. Our study shows that perturbations of a single oncogene are sufficient to deregulate the mammalian circadian clock and opens new ways in which the circadian clock can influence disease and possibly play a role in therapy.

## Introduction

All mammalian cells hold an internal circadian clock able to generate daily-endogenous rhythms with a period of approximately 24 hours. Circadian clocks are evolutionary conserved and regulate the expression of about 10% of all genes [1]–[3]. This time-generating mechanism enables the organism to react to external clues, to anticipate environmental changes and to adapt molecular and behavioural processes to specific day-times with the advantage of separating incompatible metabolic processes.

In mammals, the circadian system is hierarchically organized into two major levels of regulation including a main clock, located within the suprachiasmatic nucleus (SCN) and peripheral oscillators [4], [5]. The peripheral clocks can be found in almost all cells in the body. These are able to respond to and synchronize to output signals of the SCN clock, thereby assuring time-precision of molecular processes throughout the organism [6], [7].

Interconnected genetic networks of transcriptional and translational steps drive the oscillator in each individual clock within a cell [8], [9]. The network system can be represented by a core of two main feedback loops: the RORs/*Bmal1*/REV-ERBs loop and the PERs/CRYs loop which generate oscillations [10], [11]. The core-clock network regulates a series of clock-controlled genes (CCGs) with relevant functions in several cellular and biological processes. CCGs are involved in metabolism, detoxification, cell cycle, cell growth and survival, DNA damage responses and the immune system [12]–[15].

Malfunctions of the circadian clock have been reported in the context of many disorders [16]–[19]. Epidemiological studies have shown that increasing nocturnal light and overnight shift work coincided with a steady increase in the incidence of cancer [20]–[22]. Moreover, the long-term survival rate of patients with metastatic colorectal cancer was about 5-fold higher in those patients with normal clock compared to patients with a severely disrupted clock [23]. Additionally, accelerated malignant growth was observed in mice with an ablated SCN or subjected to experimental chronic jet-lag [24].

Hence, the clock regulation of molecular processes has severe consequences on therapy optimization, and timing of drug intake in cancer [25]–[28]. Cancer patients with altered circadian rhythms have a poorer prognosis [29] and chronotherapy, the administration of anti-cancer drugs at specific times of the day, can improve treatment efficacy as chemotherapeutics may act differently on their targets depending on the time of administration [25], . Furthermore, the rhythmic delivery of cancer therapeutics in colorectal cancer increased the efficacy of oxaliplatin on treatment and patient survival [32], [33].

A molecular basis for the effects of circadian rhythms on cancer patients might be provided by the association of several core-clock genes with cancer promoting mechanisms such as DNA damage [34] [35], metabolism [17] and cell cycle [36]–[38]. Cell cycle check point regulators such as *Wee1* (G_2_- M transition), *Myc* (G_0_- G_1_ transition), and *cyclin D1* (G_1_- S transition) have been shown to be under the direct regulation of the circadian clock and could represent one way in which the circadian clock regulates cell division [3], [18], [28]. The histone deacetylase sirtuin 1 (SIRT1), a key regulator of metabolism, has recently been identified as a core-clock component [39], [40]. The PER1 and Timeless proteins interact with proteins involved in DNA damage response and *Per1* overexpression suppresses growth of human cancer cell lines [26], [41]. Expression of *Per1* and *Per2* is downregulated in colon, breast and endometrial carcinoma [41], [42]. *Per2* is also downregulated in several human lymphoma cell lines and in non-small-cell lung cancer tissues [26], [43]–[45]. In addition, mutations in the clock gene *Npas2*, a paralog of *Clock*, have been associated with increased risk of breast cancer and non-Hodgkin's lymphoma [27], [46]. Furthermore, mutations in the gene *Clock* were found in colon cancer cell lines [47].

These results suggest the existence of a strong cross-regulation between the components of the circadian clock and proto-oncogenes or tumour suppressors. The circadian clock may act as tumour suppressor, whereas a disturbed clock might render the organism more cancer prone. However, a comprehensive view of how cancer genes and clock can influence each other is missing. This motivated us to investigate the mechanism by which the circadian clock might be altered in cancer models.

In the present manuscript, we provide a systems biology approach for the investigation of the circadian clock in several cancer cell lines including colon and skin. Surprisingly, we found strong and weak circadian oscillators within the same type of cancer. We recovered a set of genes which allow the discrimination between the two types of oscillators. Using a theoretical bioinformatics approach, we extended the core-clock network to include a larger set of clock/controlled genes involved in several biological processes and present its interconnection to the discriminative set of genes. Furthermore, we analysed the connections of such discriminative list of genes to cancer pathways among which is the RAS/MAPK pathway. Using experimental data and mathematical modelling, we provide evidence for a putative connection between both systems. Our work provides novel evidence pointing to RAS oncogene being one of the modulators of the mammalian circadian clock.

## Results

### Cancer cell lines show a rich variety of circadian phenotypes

To investigate a possible link between the circadian core-clock oscillator and tumour-associated pathways, we tested the circadian properties of colorectal cancer cell lines well characterized for their genetic properties and oncogenic pathways. The impact of the circadian systems is apparent for this cancer type, because chronotherapy has shown promising results in colon cancer patients [32], [33].

**Figure 1:**
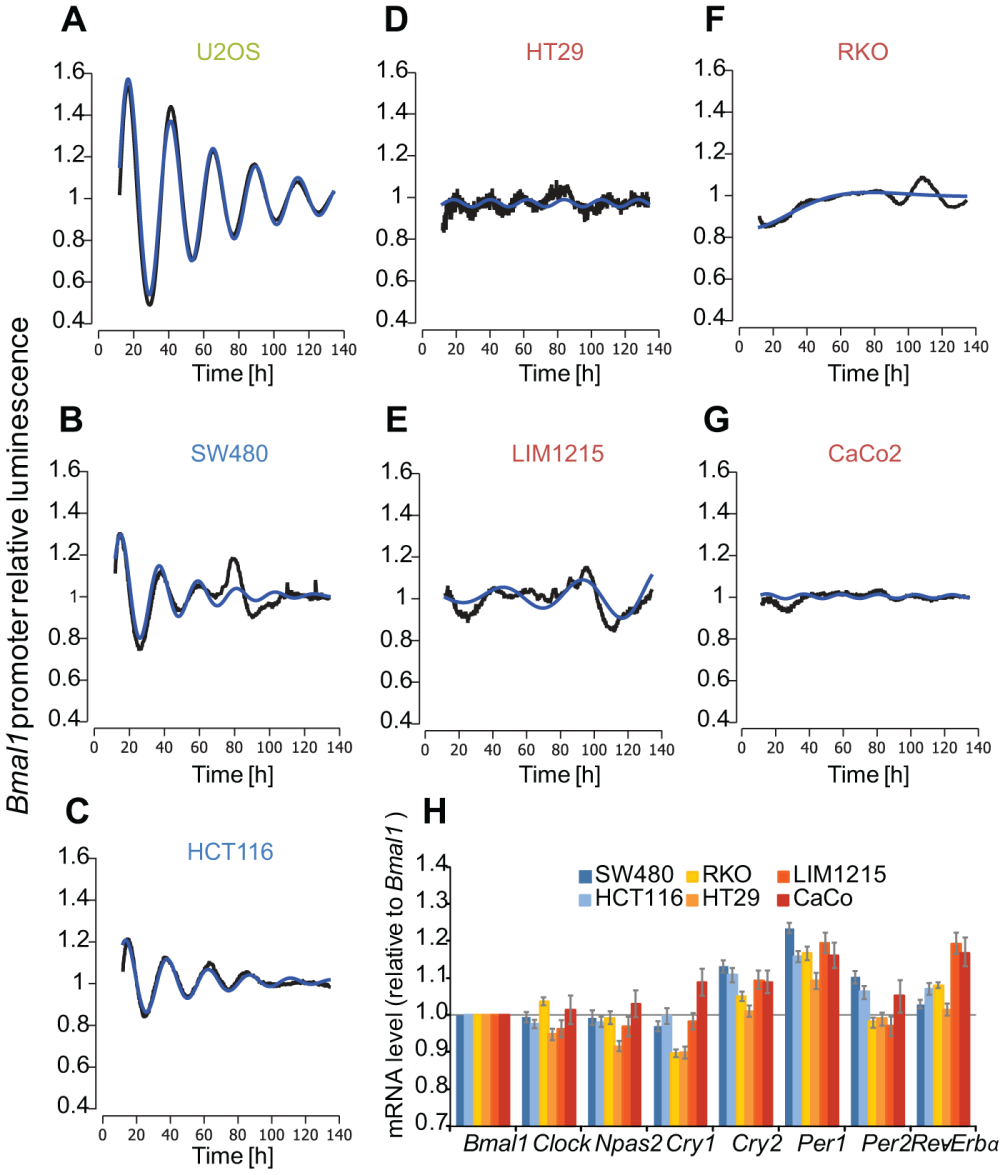
Clock phenotypes of colon cancer cell lines. (**A–G**) Cells were lentivirally transduced with a Bmal1-luciferase construct and bioluminescence was measured over 6 days. Given are detrended time series (black) and the best corresponding cosine fit (blue). Cell line names marked in green represent the test cell line (U2OS); blue - strong oscillators (SW480, HCT116); red - weak oscillators (HT29, LIM1215, RKO, CaCo2). (**H**) Plotted are the mRNA levels relative to *Bmal1* of seven clock genes for the colon cancer cells lines. Note that the bars represent ratios to *Bmal1*.

We studied the oscillation dynamics in the colon carcinoma cell lines HT29, RKO, SW480, LIM1215, CaCo2 and HTC116 using a live-cell imaging approach based on ectopic expression of a luciferase reporter construct driven by the 0.9 kb *Bmal1* promoter fragment. As a control we analysed the human osteosarcoma cell line U2OS, a widely used *in vitro* model to study properties of the mammalian circadian clock. We define a cell line with a clear circadian period and an amplitude variation of at least 20% as a strong oscillator. Surprisingly, the clock properties were very diverse among the colon cancer cell lines (Table 1), which showed strong (Figure 1B, C) and weak to no-oscillation phenotypes (Figure 1D–G). The strongly oscillating cell lines HCT116 and SW480 exhibited shorter doubling times (unpublished observations) indicating an association of perturbations of the circadian clock and effects on the cell cycle and in agreement with previous studies [48]. Moreover, we observed differences in the mRNA levels of the core-clock genes. In Figure 1H, we plotted the relative fold change to *Bmal1* for each clock gene and cell line. The highest value for *Cry1* is observed in a weak oscillator cell line (CaCo2), while the highest value for *Cry2* is observed in the strong oscillator cell lines. For *Per1* and *Per2*, the highest fold change is observed in the strong oscillators. For *Clock* and *Npas2*, weak oscillators show the highest relative change to *Bmal1*. These data clearly show a correlation between expression levels of core-clock genes and the cell oscillator phenotype.

**Table 1:** Circadian properties vary within the different cell lines.

*Bmal1* luciferase activity	U2OS	SW480	HTC116
**Period [hours]**	24.2±0.2	22.0±0.1	24.4±0.4
**amplitude**	0.78±0.05	0.41±0.01	0.28±0.04
**phase**	16.6±0.1	16.7±0.3	13.7±0.3

Indicated are values for the period, amplitude and phase of *Bmal1* luciferase reporter, for the cell lines classified as strong oscillators (n = 3, p<0.05, Student's t-test). The values for the periods, amplitudes and phases (mean ± SEM, n = 3) were determined with the ChronoStar analysis software [100].

### Microarray analysis reveals a set of genes discriminating strong and weak oscillators

**Figure 2:**
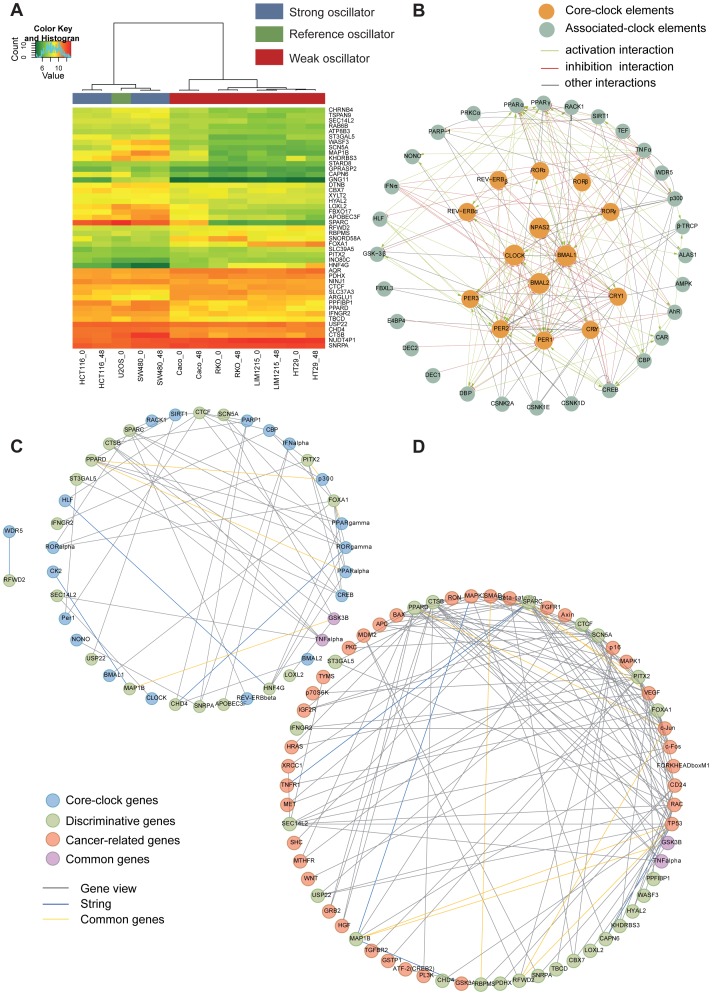
Microarray analysis reveals clock-related gene expression signature. (**A**) Shown is the heatmap generated with the list of 45 discriminative genes. Heatmaps were generated using Pearson distance function and ward clustering. Colour bar (left) indicates the expression levels for genes in the array, from green (low expression) to red (high expression). Colour bar (top) indicates class membership. Blue indicates strong oscillator, red indicates weak oscillator, and green indicates test sample (U2OS). Genes are ordered by profile similarity. (**B**) A comprehensive regulatory network of the mammalian circadian clock. We used a curated text mining approach to search for interactions between our genes of interest. The network represents 108 novel interactions, supported by 132 PubMed references. In the core of the network (orange circles) the main components of the canonical feedback loops are shown. The outer shell of the network (grey circles) shows the clock-regulated genes and proteins feeding back to the core components and thereby potentially influencing the oscillations. Red lines represent inhibitory interactions; green lines, activating interactions; grey lines, other kinds of interactions. A large format of the network is provided as Figure S2. (**C, D**) Networks of circadian and cancer regulation for the discriminative genes. All networks were generated using the GeneView software (expected rate of false positive interactions, 10%) with additional data retrieved from the STRING database. (**C**) Network of interaction correlating circadian clock genes (all genes in (B)) and discriminative genes. A large format of the network is provided as Figure S3. (**D**) Network of interaction correlating discriminative genes and cancer genes. A large format of the network is provided as Figure S4. Cancer-related genes are represented in orange, clock genes in blue, discriminative genes in green and common genes in violet (e.g. TNFα). Grey lines represent text-mined interactions, blue lines interactions from the STRING database, and yellow lines common interactions. A list of colon specific interactions (by filtering for colon cancer related terms) is given in Table S2.

To further investigate potential differences between strong and weak oscillators at the gene expression level, we performed transcriptome analysis for each cell line. A leave-one-out cross validation strategy identified a set of differentially expressed genes that discriminate between strong and weak oscillators (Figure 2A). For each cell line we excluded both replica samples once and determined the 100 most significantly expressed probe sets, allowing the identification of two clock groups using a moderated t-test to select the top genes by confidence. The resulting list of 45 best p-value discriminative genes (Table 2 and Figure 2A) allows positioning of U2OS accordingly within the strong oscillator cluster, although this cell line was not previously used for the list generation. Additionally we tested 5 other colon cancer cell lines (Colo205, SW620, SW403, HKe3, HKe-clone8 with/without mifepristone) and two keratinocyte cell lines (HaCaT and A5RT3). Data is shown in Figure S5 and Text S4. The predefined list of 45 discriminative genes could be used to correctly classify seven out of eight cell lines (Text S4). Using the binomial test, we calculated the probability of observing the same or better classification result by random chance. According to this experiment our classifier performs significantly better than randomly expected (p = 0.03516).

**Table 2:** List of week/strong oscillator cell line discriminative genes.

Gene name	Gene ID	p-value	Description (DAVID)	KEGG_PATHWAY
**APOBEC3F**	200316	1.44E-04	apolipoprotein B mRNA editing enzyme, catalytic polypeptide-like 3F	
**AQR**	9716	1.13E-05	aquarius homolog (mouse)	Spliceosome
**ARGLU1**	55082	3.55E-04	arginine and glutamate rich 1	
**ATP8B3**	148229	1.32E-04	ATPase, class I, type 8B, member 3	
**CAPN6**	827	2.94E-05	calpain 6	
**CBX7**	23492	5.13E-05	chromobox homolog 7	
**CHD4**	1108	1.56E-05	chromodomain helicase DNA binding protein 4	
**CHRNB4**	1143	9.40E-05	cholinergic receptor, nicotinic, beta 4	
**CTCF**	10664	1.06E-04	CCCTC-binding factor (zinc finger protein)	
**CTSB**	1508	1.19E-04	cathepsin B	Lysosome, antigen processing and presentation
**DTNB**	1838	1.19E-04	dystrobrevin, beta	
**FBXO17**	115290	6.52E-05	F-box protein 17	
**FOXA1**	3169	1.70E-04	forkhead box A1	
**GNG11**	2791	9.70E-06	guanine nucleotide binding protein (G protein), gamma 11	Chemokine signalling pathway
**GPRASP2**	114928	4.88E-05	G protein-coupled receptor associated sorting protein 2	
**HNF4G**	3174	3.76E-05	hepatocyte nuclear factor 4, gamma	Maturity onset diabetes of the young
**HYAL2**	8692	2.82E-05	hyaluronoglucosaminidase 2	Glycosaminoglycan degradation
**IFNGR2**	3460	5.83E-05	interferon gamma receptor 2 (interferon gamma transducer 1)	Cytokine-cytokine receptor interaction, Jak-STAT signalling pathway, Natural killer cell mediated cytotoxicity
**INO80C**	125476	1.36E-04	INO80 complex subunit C	
**KHDRBS3**	10656	5.49E-05	KH domain containing, RNA binding, signal transduction associated 3	
**LOXL2**	4017	1.26E-04	lysyl oxidase-like 2	
**MAP1B**	4131	6.93E-05	microtubule-associated protein 1B	
**NINJ1**	4814	3.18E-05	ninjurin 1	
**NUDT4P1**	440672	1.31E-04	nudix (nucleoside diphosphate linked moiety X)-type motif 4; nudix (nucleoside diphosphate linked moiety X)-type motif 4 pseudogene 1	
**PDHX**	8050	1.52E-04	pyruvate dehydrogenase complex, component X	
**PITX2**	5308	9.84E-05	paired-like homeodomain 2	TGF-beta signalling pathway
**PPARδ**	5467	1.96E-05	peroxisome proliferator-activated receptor delta	PPAR signalling pathway, Wnt signalling pathway, pathways in cancer, acute myeloid leukemia
**PPFIBP1**	8496	9.75E-05	PTPRF interacting protein, binding protein 1 (liprin beta 1)	
**RAB6B**	51560	6.76E-05	RAB6B, member RAS oncogene family	
**RBPMS**	11030	1.16E-04	RNA binding protein with multiple splicing	
**RFWD2**	64326	6.53E-05	ring finger and WD repeat domain 2	p53 signalling pathway, hsa04120:Ubiquitin mediated proteolysis
**SCN5α**	6331	1.06E-04	sodium channel, voltage-gated, type V, alpha subunit	
**SEC14L2**	23541	7.94E-05	SEC14-like 2 (*S. cerevisiae*)	
**SLC37A3**	84255	5.70E-05	solute carrier family 37 (glycerol-3-phosphate transporter), member 3	
**SLC39A5**	283375	8.42E-05	solute carrier family 39 (metal ion transporter), member 5	
**SNORD58A**	26791	3.04E-04	U58 small nucleolar RNA	
**SNRPA**	6626	1.70E-04	small nuclear ribonucleoprotein polypeptide A	Spliceosome
**SPARC**	6678	2.15E-05	secreted protein, acidic, cysteine-rich (osteonectin)	
**ST3GAL5**	8869	1.02E-04	ST3 beta-galactoside alpha-2,3-sialyltransferase 5	Glycosphingolipid biosynthesis
**STARD8**	9754	1.33E-04	StAR-related lipid transfer (START) domain containing 8	
**TBCD**	6904	4.37E-05	tubulin folding cofactor D	
**TSPAN9**	10867	4.31E-05	tetraspanin 9	
**USP22**	23326	3.03E-04	ubiquitin specific peptidase 22	
**WASF3**	10810	3.81E-06	WAS protein family, member 3	Adherens junction, Fc gamma R-mediated phagocytosis
**XYLT2**	64132	4.02E-05	xylosyltransferase II	Chondroitin sulfate biosynthesis, heparan sulfate biosynthesis

Raw p-values are derived using moderated t-test between groups of strong versus weak oscillators.

Moreover, previous data [49] shows that the siRNA-dependent -knockdown of the majority of these 45 genes confers a circadian phenotype in U2OS cell lines, underlining their potential importance as regulators of the circadian system.

The methodology used to generate the list of 45 discriminative genes, including their p-values, is explained in detail in Text S1 and the resulting p-values for the short list of 45 genes are additionally given in Table 2.

The composition of the set of top genes indicates that phenotypic circadian clock differences are reflected by gene expression differences both in genes of the core network (Figure 1H), but also in additional genes not directly associated with circadian clock functions.

### The mammalian circadian clock – an extended regulatory network of a time-generating mechanism

To explore a potential correlation of the discriminative set of genes to the mammalian circadian clock we extend the known core-clock network, which encompasses several genes and proteins interconnected by positive and negative feedback loops [10], to a layer of next neighbours. We scanned a total of 21.4 million abstracts by text mining. After careful curation of the text mining results, we assembled a comprehensive genetic network for the mammalian circadian clock (Figure 2B). First, we gathered a network containing the currently known core-elements of the circadian pathway including the 14 genes: *Per1,2,3*, *Cry1,2*, *Bmal1,2*, *Rev-Erbα,β, Rorα,β,γ*, *Clock* and *Npas2*. In the next step, we added 16 elements reported to be directly interacting to this core set [13]. Subsequently, we used our text mining software GeneView [50] to extract all reported interactions between (i) any two elements of the network and (ii) new elements with direct connections to the 14-element core. As a validation of the performance of GeneView, we analysed the part of the network containing common elements to our previously published network [13]. The software provided evidence for 85% of the interactions described (manuscript in preparation). Additionally, we found 17 new elements in the outer shell and 108 novel interactions, after curation, supported by 132 PubMed references. The enriched circadian-core network contains a total of 47 elements and 229 interactions (Figure 2B, Text S2). This network provides an improved level of specificity regarding interactions of core-clock and clock-controlled genes, in comparison to previously published networks [12], [13]. It includes both protein-protein and DNA-protein interactions and selectively assembles elements which are as well reported to be able to influence the core-clock Figure 2B.

To analyse whether and how the network might convey circadian information to specific output pathways, we performed a detailed analysis of the KEGG pathways associated with the different elements as well as of the data set obtained by text mining analysis (Text S2). We found that many of the genes in the periphery of the network play important roles in pathways frequently deregulated in cancer. Examples are the Wnt pathway (CSNK1ε, CSNK2α, p300, GSK3β, βTRC), the TGF-β signalling pathway (CBP, p300, TNFα), the Jak-STAT signalling pathway (CBP, p300, IFNα) and the MAPK signalling pathway (CBP, p300, GSK3β, PPARγ, TNFα). We also found many genes involved in cell cycle and repair mechanisms (NONO, PPAR1, GSK3β) and in immune defense (CREB, GSK3β, AMPK, TNFα, PGC-1α). Moreover, many of the new network elements were also found to link the circadian system to metabolism and xenobiotics detoxification mechanisms and as such are also potentially relevant in terms of therapy and drug response (PPARα, TEF, ALAS1, AhR, CAR, HLF, E4BP4, DEC2, DEC1, DBP).

Altogether, these results clearly support the view that the circadian clock and oncogenic pathways are strongly connected.

### A comprehensive network of circadian regulation in tumourigenesis

To investigate how the identified set of discriminative genes links cancer genes and circadian regulators we again used the text mining software GeneView to create interaction networks between i) the core-clock genes (Figure 2B) and the 45 discriminative genes (Table 2, Text S1); ii) a set of known colon cancer-related genes ([51], Table 3) and the 45 discriminative genes and iii) between all three sets of genes. Only interactions involving elements of two different gene sets were considered (Figure 2B, C). These interactions include both protein-protein as well as DNA-protein interactions. All interactions extracted by the text mining pipeline as well as 391,434 interactions contained in the STRING database version 9.0 [52] were collected. In total 646 Interactions were found to be involved in the assembly of the network (Figure S1). We found 184 connections between our discriminative set of genes and the genes of interest (clock genes and cancer genes, Figure 2C, D). To test whether the number and counts of interactions point to a specific connection between the discriminative genes and the clock/cancer genes, we now tested the significance of the number of connections by comparing to networks build from random gene sets of the same size (45 genes). We created a total of 50 such random sets where each gene of the set was randomly selected from a bin containing genes with the same number of PubMed citations as a gene in the discriminative set. We obtained an average of 165 interactions, standard deviation 28 yielding a p-value of 4.4e-5 when using a non-parametric Wilcoxon-Test. This indicates that a specific connection between the discriminative genes and the clock/cancer genes exists, which points to the relevance of this gene set in the clock-cancer context.

**Table 3:** Selected cancer-related genes, core-clock genes and clock-related genes.

Cancer-related Genes			Core and clock-related genes	
AKT (RAC)	MCC1	VEGF	AHR	REV-ERBα
APC	MDM2	WNT	ALAS1	REV-ERBβ
ATF-2 (CREB 2)	MEK	XRCC1	AMPK	RORα
Axin	MEKK1	XRCC3	βTRCP	RORβ
BAD	MET		BMAL1	RORγ
BAX	MTHFR		BMAL2	SIRT1
b-catenin	NRAS		CAR	TEF
BRAF	PAK		CBP	TNFα
CD21	PDK1		CK2	WDR5
CD24	PRKC-α		CLOCK	
CDC4	p16		CREB	
c-Fos	p70S6K		CRY1	
c-Jun	PL3K		CRY2	
DPD (DPYD)	PLA2		CSNK1δ	
DSH	PLA2G10		CSNK1ε	
DUSP16	PLC		CSNK2α	
ELK1	PLD		DBP	
EPHB2	RAF		Dec1	
ERCC1	RAL		Dec2	
ERCC2	RALGDS		E4BP4	
ERK	RBP		FBXL3	
FGFR1	Rho		GSK3β	
FORKHEAD box M1	RON		HLF	
FZD7	RSK		IFNα	
GRB2	SFRP4		NONO	
GSK3α	SHC		NPAS2	
GSK3β	SMAD4		p300	
GSTP1	SOS		PARP1	
HGF	TCF4		Per1	
HRAS	TGFBR2		Per2	
IGF2R	TNFα		Per3	
JNKK	TNFR1		PPARα	
KRAS	TP53		PPARγ	
MAPK1	TYMS		PRKC-α	
MAP2K2	UGT1A1		RACK1	

Detailed information on selected genes is provided in Text S2 and 3.

Furthermore, from the network analysis, we found that 20 out of the 45 discriminative genes were associated with clock genes (Figure 2C), 27 were found to be associated with cancer-related genes (Figure 2D) among which 18 intersect with the set of clock genes (Table S1). Discriminative genes associated with cancer pathways (Table 2) include *IFNGR2* (involved in the Jak-STAT pathway) [53], *PITX2* (TGF-β pathway), *RFWD2* (p53 signalling), *PPARγ* (Wnt pathway). Moreover, several genes are also involved in the MAPK/RAS pathway: *LOXL2*, *PPARD* and *CTSB* are RAS target genes; *Rab6* is a RAS family member; SPARC is also targeted by the RAS pathway and it was also shown to be a key modulator of extra-cellular matrix (ECM) remodelling, it affects cell proliferation and differentiation and it was recently reported to downregulate VEGF and thereby suppressing angiogenesis. In addition, we searched among our genes of interest for circadian properties by evaluating their expression profiles in published microarray data. We found 83% of the cancer related genes and 24% of the discriminative genes to show measurable oscillations in gene expression in several tissues and cell lines (Text S3).

Taken together, these discriminative genes are likely relevant for the analysis of possible clock malfunctions in a cancer model as a clear connection of these 45 genes to core-clock genes and to distinct cancer-related genes exists.

### Functional coupling of Ras oncogene signalling and the circadian clock

**Figure 3:**
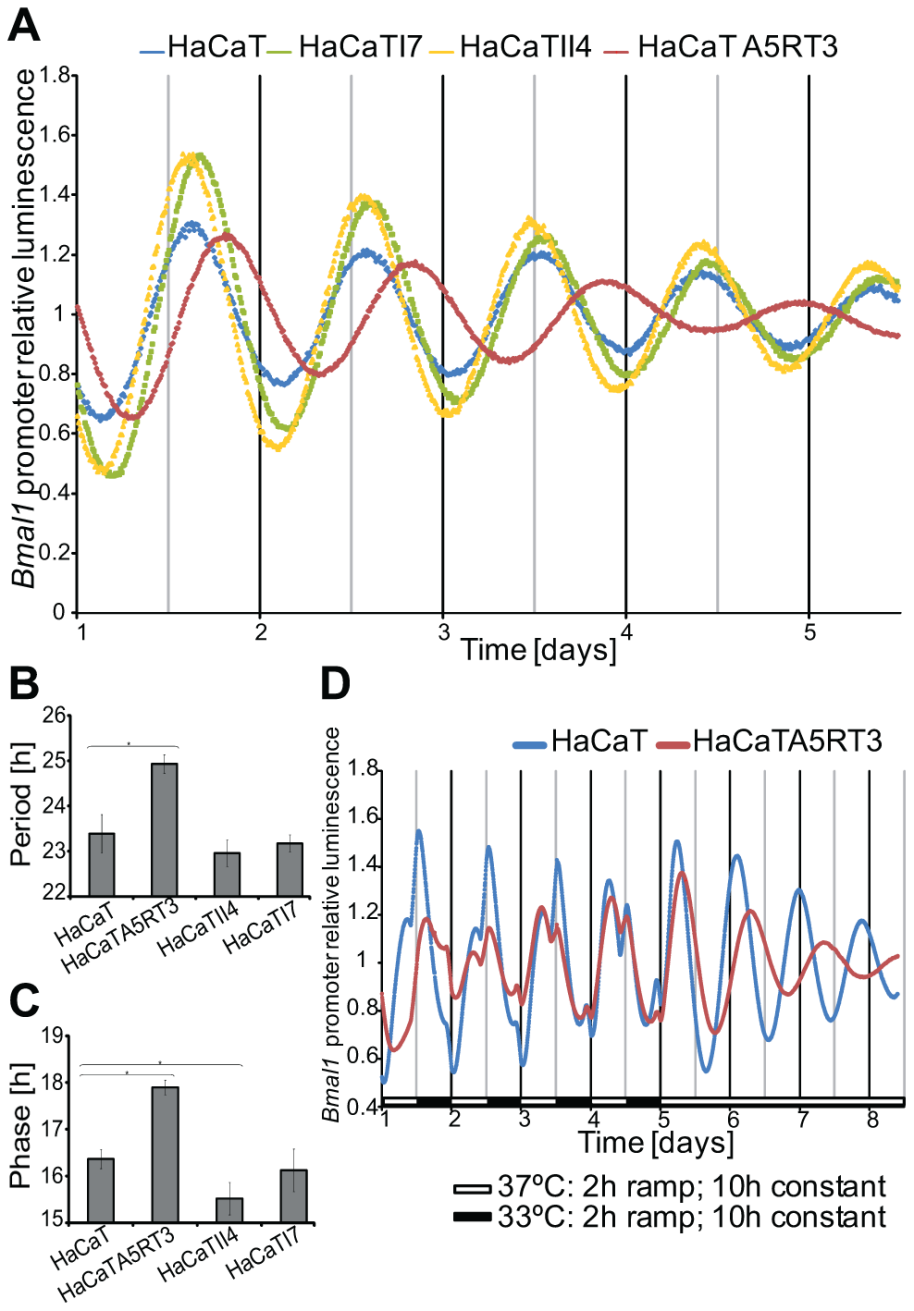
Differential circadian phenotypes of human keratinocytes and their Ras-transformed variants. *Bmal1*-Luciferase bioluminescence was recorded for 5 days after dexamethasone synchronization (**A**). Representative results from five independent experiments are shown (HaCaT II4 n = 3). Normal human keratinocytes display an average period of 23.4±0.4 hours with a peak phase at 16.4±0.2 hours after of synchronization (**B, C**). HRAS transformed HaCaT A5RT3 show a significantly longer period (HaCaT A5RT3 24.9±0.2 hours p<0.05, Student's *t*-test) and a significant phase delay of approximately 1.5 hours (p<0.05, Student's *t*-test). An significantly earlier phase of 0.24 and 0.85 hours was observed in HaCaT I7 and HaCaT II4 cell lines, compared to normal keratinocytes (HaCaT II4, p<0.05, Student's *t*-test). (**D**) Phase delay of H-Ras-transformed human keratinocytes upon temperature entrainment. HaCaT and HaCaT A5RT3 were entrained with temperature cycles for 4 days consisting of 10 hours 37°C and 10 hours 33°C with 2-hour ramps and subsequently released to constant 37°C. Bioluminescence of the *Bmal1*-driven luciferase reporter was recorded for at least 8 days. Shown are representative detrended data from two independent experiments.

The link of the circadian network to distinct cancer related pathways such as RAS/MAPK, Wnt and Jak/STAT led us to investigate the connection of clock regulation and signalling pathways in an experimental model. As a model pathway, we chose RAS/MAPK signalling, which is one of the most frequently altered signalling pathways in human cancer. *KRAS* mutations have a high prevalence in colorectal cancers. However, the colon cancer cells analysed exhibited a highly variable genetic background and thus introduced an extra level of complexity when used as models for functional analysis and for studying deregulation of the circadian clock. Therefore, we used a well-established *in vitro* epithelial model, in which cellular transformation is triggered by the RAS oncogene, that simulates carcinogenesis development, for investigating the influence of RAS transformation on the circadian clock [54]. Human HaCaT skin keratinocytes (and their derivatives represent the different steps of malignant epithelial conversion from the immortal state (HaCaT), to benign (HaCaT I7, class I tumours), advanced (HaCaT II4, class II tumours) and metastatic states (HaCaT A5RT3). The different HaCaT lines were lentivirally transduced with the *Bmal1*-driven luciferase reporter and the oscillation dynamics was monitored for 5 days. Non-transformed human keratinocytes exhibited an average period of τ = 23.4±0.4 hours (mean ± SEM, n = 5) with a strong oscillation that persisted for several days (Figure 3A). HaCaT I7 and HaCaT II4 periods were not significantly different compared to the immortalized cell line HaCaT (τ = 23.18±0.19 hours, n = 5 and τ = 22.96±0.3 hours, n = 3, respectively). However, the metastatic cell line HaCaT A5RT3 showed a significantly longer period of τ = 24.93±0.2 hours (n = 5), than that observed in normal HaCaT cells (*p*<0.05, Student's *t*-test) (Figure 3B). Moreover, HaCaT A5RT3 showed a significantly delayed phase of approximately 1.5 hours (17.9±0.15 hours) compared to HaCaT cells (16.37±0.21 hours; n = 5, *p*<0.05, Student's *t*-test). HaCaT II4 showed the opposite effect, having an advanced phase of approximately 0.85 hour significantly earlier than in normal keratinocytes (15.52±0.35 hours; n = 3 *p*<0.05, Student's *t*-test) (Figure 3C). A larger amplitude was observed in both HaCaT I7 and II4 (1.49±0.04, and 1.52±0.05, respectively) compared to normal keratinocytes (1.36±0.03) or HaCaT A5RT3 (1.33±0.02) (Figure 3A). To rule out possible secondary effects due to cell growth alterations caused by transfection with the BMAL1-promoter-driven luciferase (BLP) construct, proliferation of cells was measured by determining increased conductance of monolayers over time, using XCelligence technology (Roche) (unpublished observations).

To investigate if the observed HaCaT A5RT3 phenotype is indeed due to a different phase of the oscillator, we carried out a temperature entrainment assay with metastatic HaCaT A5RT3 and immortal HaCaT cells. This technique has the advantage that the cells do not respond to a single pulse of an external signal, but are entrained to follow an environmental cue (temperature) along a period of time. Bioluminescence measurement for at least 3 days following the entrainment revealed that HRAS transformed human keratinocytes A5RT3 indeed have a different circadian phenotype in comparison to normal keratinocytes (Figure 3D). The first peak after release of the cells to a constant temperature was used to determine the phase of entrainment. H-Ras-transformed HaCaT A5RT3 cells showed an advanced phase, of approximately 6 hours (Figure 3D). These data demonstrated that Ras transformation can induce a phase shift in the *in vitro* model system which is consistent with the observed period and phase perturbations in the circadian phenotype of A5RT3 cells.

**Figure 4:**
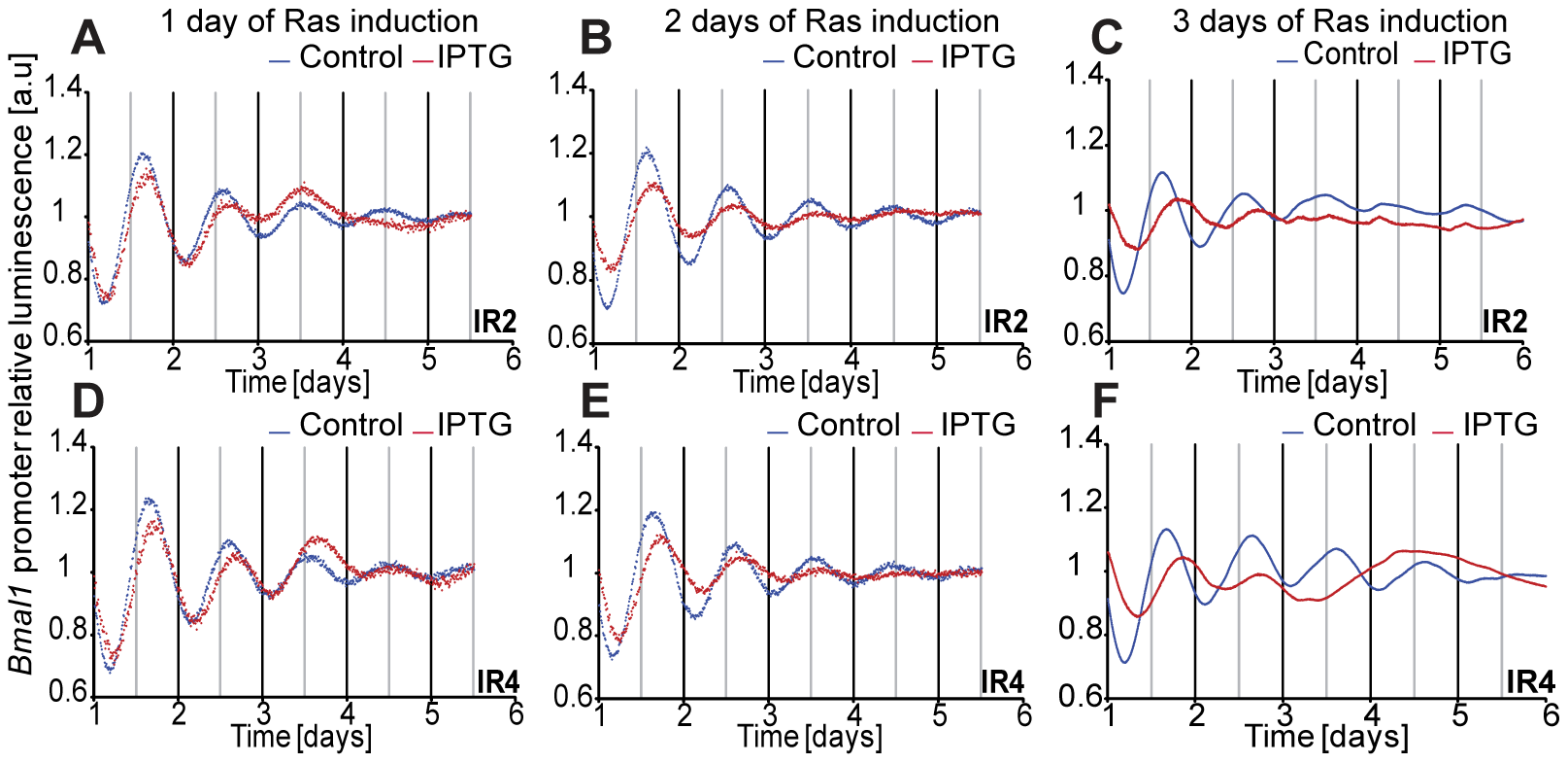
Induction of Ras expression in rat fibroblasts. IR2 and IR4 rat fibroblasts (1×10^5^ cells) were plated in 35 mm dishes 24 hours previous to measurement, and treated with either 20 mM IPTG for Ras induction (red line), or with sterile H_2_O (blue line) immediately after synchronization with dexamethasone (**A**) for IR2 and (**D**) for IR4, or for 48 hours previous to real-time rhythmicity measurement (**B**) for IR2 and (**E**) for IR4. 0.5×10^5^ IR2 (**C**) and IR4 (**F**) cells were plated in 35 mm dishes and treated with either 20 mM IPTG for Ras induction (red line), or with sterile H_2_O (blue line) for 72 hours previous to synchronization and bioluminescence was monitored for several days (A and B: 6 counts/h in LumiCycle; C: 12 counts/h in TopCount). Shown are representative detrended data from three independent experiments.

**Figure 5:**
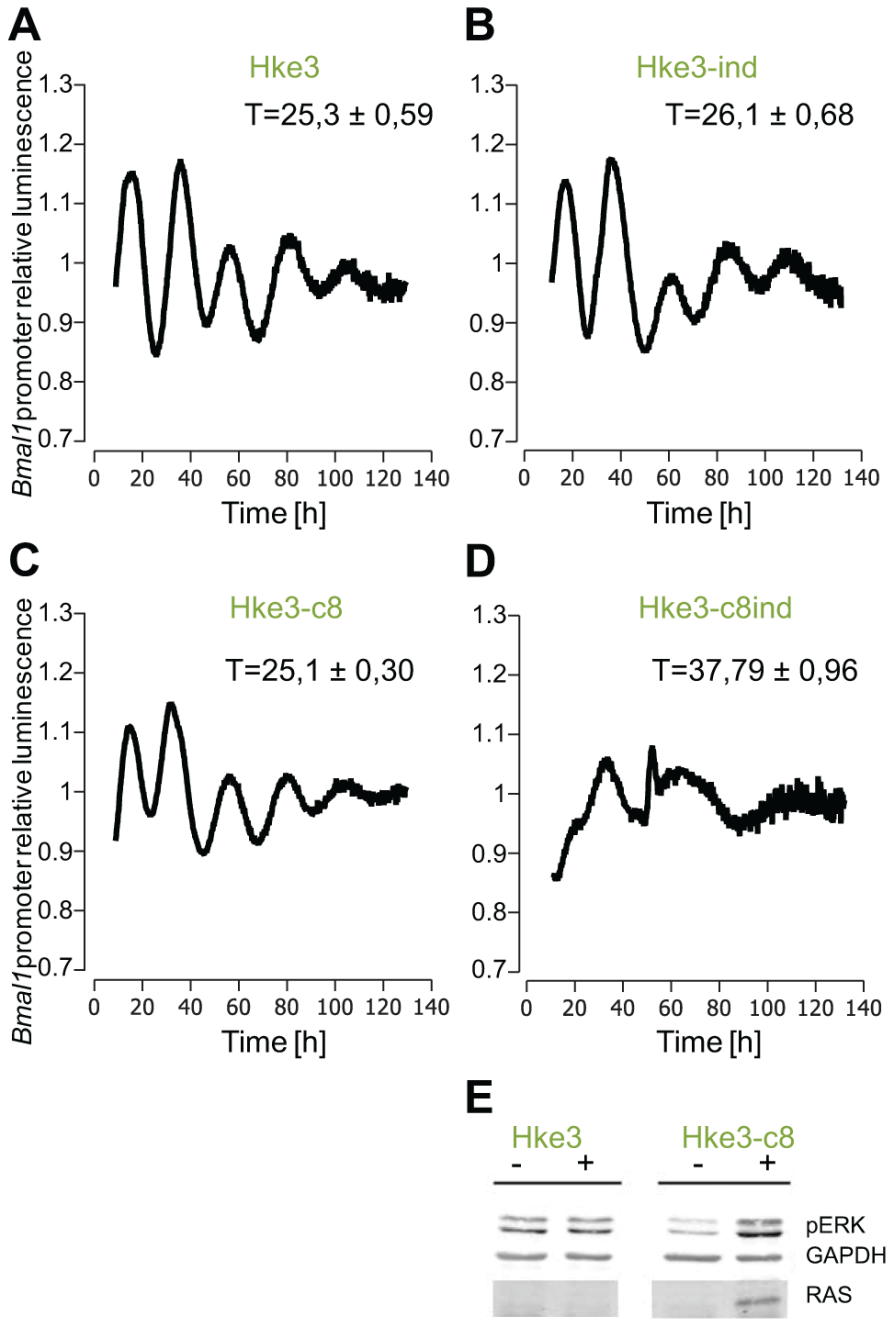
Effect of KRAS induction in HKe3 and HKe3 clone 8 cell lines. Shown are the results from 3 independent experiments. (**A**) HKe3 have a period of 25.3±0.59 hours (p<0.05, Student's *t*-test), (**B**) HKe3 cells treated with mifepristone show a small period increase (26.1±0.68 hours p<0.05, Student's *t*-test). (**C**) HKe3 clone8 present a period of 25.1±0.3 hours (p<0.05, Student's *t*-test). (**D**) HKe3 clone8 cells treated with mifepristone show a large period increase (37.79±0.96 hours p<0.05, Student's *t*-test). (**E**) Western Blot analysis for RAS and phosphorylated ERK in HKe3 and HKe3 clone8 cells following K-Ras induction by mifepristone. Extracts where prepared from untreated cells (−) or 48 hours after addition of mifepristone (+). Levels of RAS and phosphorylated ERK were analysed. GAPDH was used as loading control.

To validate these results in an inducible RAS-system, we took the rat fibroblast cell line, 208F, and two derivative clones: IR2 and IR4. 208F cells are preneoplastic rat immortal fibroblasts. IR2 and IR4 cell lines are clones obtained by stable transfection with the H-*Ras* oncogene (G12V), which is under the *lac* regulatory control harbouring a Ras IPTG-inducible promoter [55]. All cell lines exhibited oscillations with a period of approximately 24 hours. The effect of the overexpression of H-Ras in rat fibroblasts was a clear phase shift in IPTG-treated cells (Figure 4). The levels of RAS and phosphorylated ERK were analysed via Western Blot and are depicted in Figure S6. The results obtained with the HaCaT system and the rat fibroblast cell lines could be reproduced with colorectal cancer cells HKe3 and are presented in Figure 5. The HKe3 cells are derived from HCT116 colorectal cancer cell lines, but KRAS has been disrupted by genetic recombination (HKe3; [56]). HKe3 cells exhibit a period of τ = 25.3±0.59 hours. Furthermore, we used the Hke3-clone 8, in which we introduced a conditional KRASV12 oncogene. In the absence of KRAS induction Hke3 clone 8 exhibits a period of (τ = 25.1±0.3 hours), similar to HKe3. Most interestingly, we observe a clear period phenotype upon activation of the KRAS oncogene (τ = 37.9±0.96 hours). This resembles our previous observations in HaCaT and IR2 cells.

With this data we could confirm our previous results and clearly showed that upon induction of RAS a larger period phenotype could be measured.

### Clock genes are differentially expressed in human keratinocytes

**Figure 6:**
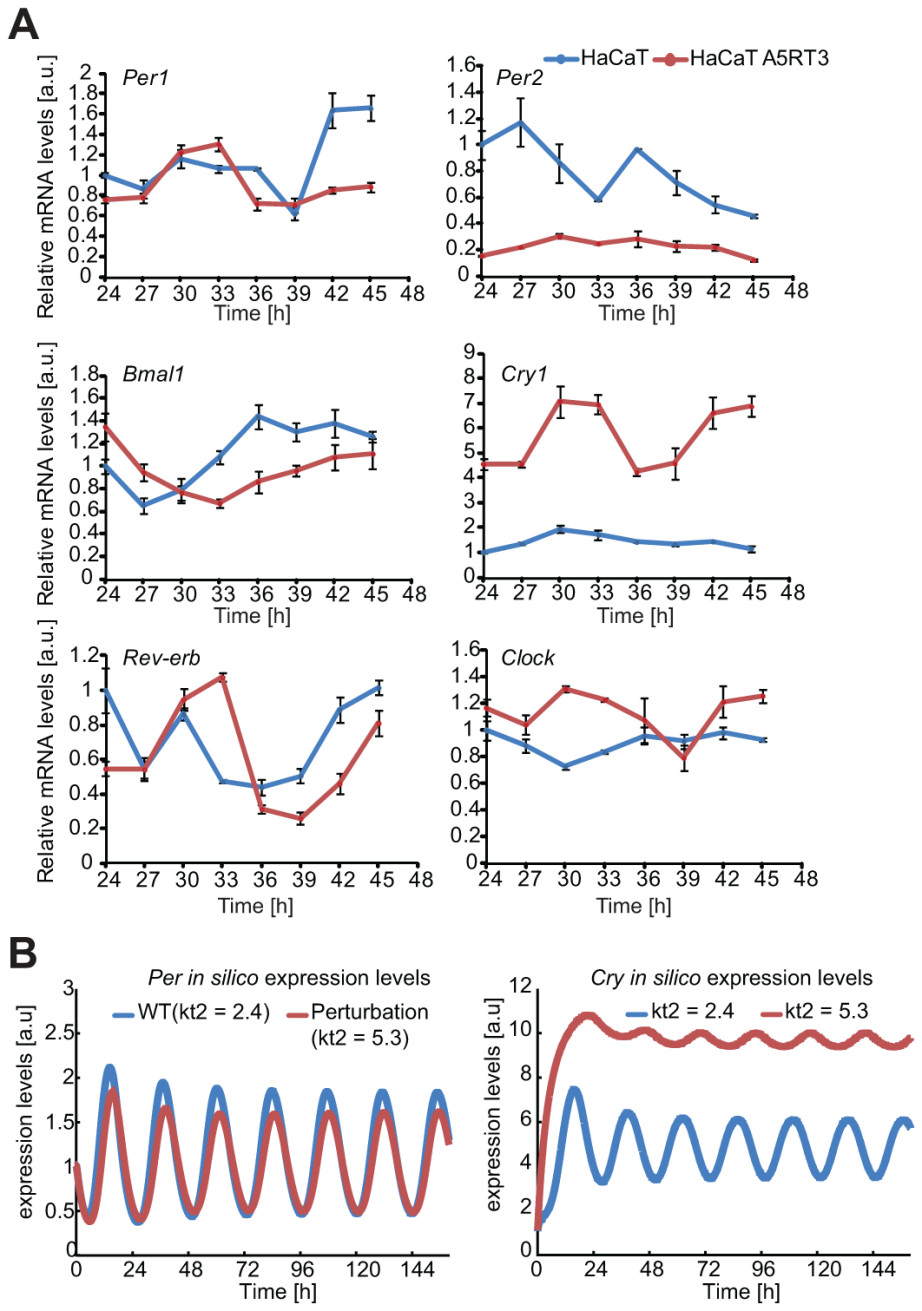
Differential gene expression of core clock genes in normal- and H-Ras-transformed human keratinocytes. (**A**) HaCaT and H-Ras transformed HaCaT A5RT3 cells were synchronized with dexamethasone and after 24 hours harvested in regular 3-hour intervals *Cry1* and *Per2* gene expression magnitudes (relative to *Gapdh*) in HaCaT and HaCaT A5RT3 cells were significantly different (p<0.001, Mann-Whitney U-test). Shown are means and SEM of three independent experiments. (**B**) Shown are *in silico* expression profiles for *Per* and *Cry* obtained from simulations with our model over approximately 6 days. The blue curve represents the wild type (WT) non-perturbed situation (τ = 23 hours) and the red line represents the result of a single parameter perturbation (τ = 23.7 hours).

To investigate the clock specific changes at the gene expression level in human keratinocytes, we performed real-time PCR for the clock genes *Cry1*, *Bmal1*, *Per2*, *Rev-Erb*α and *Clock*. The measurement was started 24 hours after synchronization to avoid influence of immediate early gene response that may not reflect an accurate effect of the oscillator. We measured the expression of the five clock genes in HaCaT and HaCaT A5RT3 cells over the course of 24 hours and all showed clear oscillations (Figure 6A).

We found marked differences in the levels of *Bmal1*, *Per2*, *Cry1* and *Clock mRNAs*, while *Per1* and *Rev-Erbα mRNAs* were largely similar in both cell lines. Consistent with the live-cell oscillation dynamics (Figure 3), *Bmal1* transcript levels oscillate with a phase about 6 hours shorter in HaCaT cells than in HaCaT A5RT3 cells. While *Per2 mRNA* levels are strongly reduced in H-Ras transformed HaCaT cells, *Cry1* and *Clock* gene expression is markedly increased in H-Ras transformed keratinocytes compared to the HaCaT cells, where C*ry1* gene expression remained at low levels.

Overall, gene expression in both cell lines is divergent, especially for *Cry1*, where mRNA levels increased and for *Per2* with decreased levels, in H-Ras transformed keratinocytes. This indicates that oncogenic RAS is likely to directly impinge onto the regulation of the circadian clock, however by yet unknown means.

### Perturbations in BMAL activity lead to changes in the period

**Figure 7:**
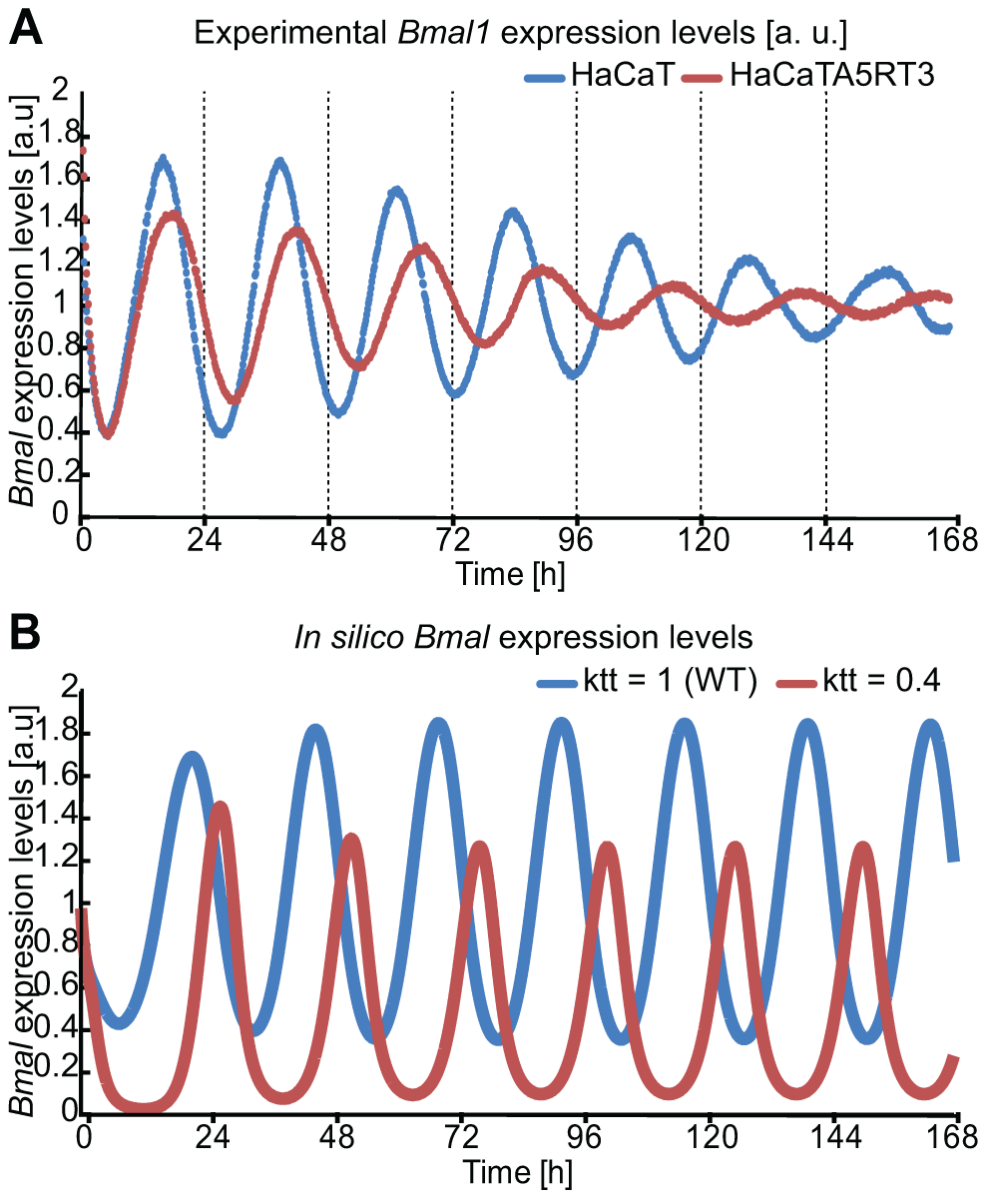
In silico perturbation of BMAL-mediated transcription reproduces HaCaT vs A5RT3 phenotype. (**A**) HaCaT and HRAS transformed HaCaT A5RT3 cells were synchronized with dexamethasone and after 24 hours harvested in regular 3-hour intervals. Shown are representative results from three independent experiments. Normal human keratinocytes shows an average free-running period of 22.9±0.2 hours (p<0.05, Student's *t*-test), and a peak at 15.9±0.4 hours (p<0.05, Student's *t*-test) after synchronization. H-Ras transformed HaCaT A5RT3, present a wide period (HaCaT A5RT3 24.5±0.1 hours p<0.05, Student's *t*-test) and a peak at 16.9±0.05 h (p<0.05, Student's *t*-test). (**B**) Shown are *in silico* expression profiles for *Bmal1* obtained from simulations with our model over approximately 7 days. The blue curve represents the wild type (WT) non-perturbed situation (τ = 23 hours) and the red line represents the result of a perturbation in BMAL-mediated transcription (τ = 24.1 hours).

To unravel potential underlying mechanisms of RAS-mediated clock alterations we used our previously developed mathematical model for the mammalian circadian clock and carried out a control coefficient analysis over all model parameters and analysed the effects on period and magnitude [11]. We filtered for parameters for which a perturbation could induce antipodal changes in the magnitude of *Per* and *Cry* and at the same time an increase in the period. By perturbing a parameter involved in the transcription of the *Cry* gene (kt2, [11]) such an effect could be simulated (Figure 5B). Combinations of several parameters could cause a similar effect. However, in our mathematical model-system, the parameter described was the only which could, on its own, cause the observed phenotype. Interestingly, in our model the parameter kt2 (*Cry* transcription equation [11]) regulates BMAL1/CLOCK-mediated transcription on *Cry* proposing one possible way of how RAS might interfere with the circadian rhythms: weakening BMAL1's role as a transcriptional activator (perturbation of transcription activation for all clock genes [11]). Figure 7 predicts a gene expression profile in agreement with the experimental data suggesting that RAS activation perturbs the clock possibly via modulating BMAL1/CLOCK transactivation activity.

### RAS/MAPK pathway activation is responsible for the phenotype

**Figure 8:**
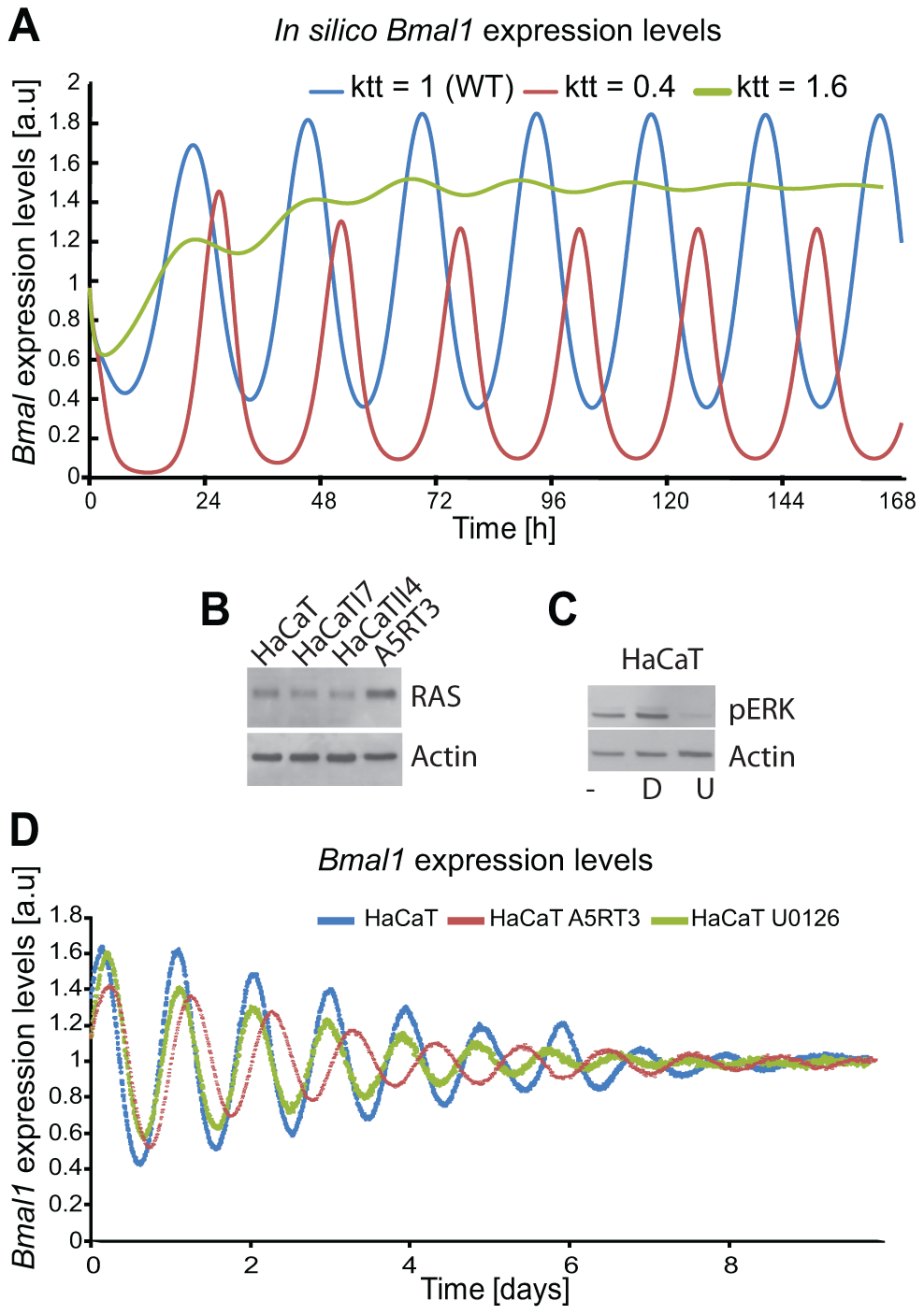
Ras transformation affects the circadian clock via MAPK signalling. (**A**) Shown are *in silico*-generated gene expression oscillations with wild type (WT) in blue, RAS overexpressing in red (i.e., reducing the BMAL1 mediated-transcription by 60% (ktt = 0.4) compared to WT) and reducing RAS activity (i.e., increasing BMAL1 mediated-transcription by 60% (ktt = 1.6)) in green. (**B**) Western Blot analysis of RAS protein expression for the HaCaT cell lines and its derivatives (HaCaTI7, HaCaTII4, A5RT3), shows clear RAS overexpression measured in A5RT3. (**C**) Phosphorylated ERK (pERK) is shown for HaCaT cells under different conditions: non-treated cells, (−), cells treated with DMSO (D), cells treated with U0126 (U). HaCaT cells were treated with a MEK inhibitor (UO126 20 µmol) after synchronization with dexamethasone and circadian activity of *Bmal1*-luciferase reporter was measured over 8 days. (**D**) Shown are the results from 3 independent experiments. Non-treated (NT) HaCaT have a period of 22.9±0.2 hours (p<0.05, Student's *t*-test), HaCaT A5RT3 present a larger period of 24.5±0.1 hours (p<0.05, Student's *t*-test). Cells treated with UO126 (20 µM) show a period decrease (22.2±0.1 hours, p<0.05, Student's *t*-test) and a phase advance.

To test our hypothesis regarding the regulatory effect of RAS on the circadian clock, we first investigated the potential influence of the MAPK/RAS signalling pathway on the circadian phenotype in HaCaT cells. In our model the activation of RAS/MAPK signalling (60% reduction of the parameter which regulates BMAL1-mediated transcription, for each gene) predicts an increase of the period (τ = 24.1 hours), while inhibition of the RAS/MAPK pathway (60% increase in the parameter which regulates BMAL1-mediated transcription, for each gene) led to a shorter period phenotype (τ = 21.4 hours), as shown by the *in silico* expression profiles of *Bmal1* (Figure 8A). To test the predicted effect of reducing RAS/MAPK-mediated signalling on the circadian phenotype experimentally, we treated synchronized HaCaT cells with the MEK inhibitor U0126 (Figure 8B, C). Indeed, U0126-treated cells showed a shorter period compared to vehicle-treated cells (Figure 8D). In HaCaT A5RT3, the MAPK pathway seems to impinge on the length of the period. This can be seen upon the comparison of HaCaT and A5RT3 and now much stronger when we inhibit the MAPK pathway in A5RT3 (Figure S7).

Together, our theoretical and experimental data indicate that the activity of the RAS/MAPK modulates the circadian period (and thereby also the entrained phase), possibly by influencing the transcriptional activity of the CLOCK/BMAL1.

## Discussion

Perturbations of the circadian clock have been described in different pathological conditions. In model systems, genetic manipulations and read-out of the circadian clock were found associated with distinct phenotypes such as insulin resistance and obesity [57], premature aging [58], increased tumour development [28] and altered stem cell homeostasis [59]. This diversity of phenotypes reflects the fact that a large number of genes controlling crucial cellular processes such as the cell cycle, DNA repair and metabolic processes are known to be regulated in a circadian manner. Despite these clear functional associations between clock and disease, there are few mechanistic studies investigating possible molecular processes involved in the interconnection between cancer and clock in a systematic way. In this manuscript, we set out to extensively analyse molecular alterations, in colon and skin cancer, which could bring us closer to understand the processes and mechanism involved in how the deregulation of the circadian clock takes place in carcinogenesis.

### De-regulation of clock at multiple levels: Gene expression, signalling transduction

In this study, we showed that the clock is perturbed differentially within the same cancer type and observed a rich variety of clock phenotypes. To explain these effects, we correlated the gene expression profiles with the clock phenotype of the cells. This analysis revealed a set of genes discriminating between strong and weak oscillator, thus functioning as a clock phenotype predictor. In fact, this set of genes also allowed the correct classification of the osteosarcoma test cell line U2OS. Moreover, previous data [49] showed that the siRNA dependent-knockdown of the majority of these 45 genes confers a circadian phenotype in U2OS cell lines, underlining their potential importance as regulators of the circadian system. To what extend this set of genes exhibits robustness as a clock phenotype predictor beyond our experimental setup is currently unknown. Of note, Caco2 cells have been described by others as a good oscillators, yet the experimental conditions were different [60]. Thus, nature of the predictive gene set might undergo some alterations when alternative conditions are used.

The microarray data revealed important clock genes such as *Nono* (a known splicing factor and recently described as connecting the clock to the cell cycle via PER [36]), *Rac* (involved in proliferation) and *CkII*α to be highly expressed in all cell lines. At the same time, the tumour suppressor gene *Per3* appears weakly expressed in all cell lines as expected. In addition, we identified a characteristic differential expression of several genes encoding epigenetic regulators, signalling molecules and transcriptional regulators not previously connected to circadian rhythm. *CBX7* is an essential component of the polycomb repressive complex 1 (PRC1) involved in the control of histone methylation at tumour suppressor loci such as p16 [61]. *CHD*, encodes a chromodomain regulator of chromatin remodelling and was lost in approximately 50% of colorectal cancers [62]. The YY1 interacting chromatin remodelling complex factor INO80 [63] and the ubiquitous epigenetic regulator and insulator protein CTCF, are associated with the weak oscillators. CTCF controls long-range chromatin interactions and functions in the establishment and maintenance of epigenetic signatures. This renders CTCF a potentially important factor also for controlling circadian genes [64]–[66]. A link between the circadian clock, energy metabolism and epigenetic (re)programming has been established earlier [67]–[69]. Our new observations indicate that epigenetic events involving DNA methylation, histone modification and chromatin remodelling might also induce differential circadian oscillation in tumour cells.

Differential gene expression between strong and weak oscillators was also seen for the signalling molecules *LOXL2*, *SPARC*, *CTSB*, *IFNGR*, *WASF3*, *GNG11* and the metabolism-associated *PPARD* gene [70]. High expression of these genes was strongly associated with the strong oscillator phenotype, but low expression with the weak oscillators. In contrast, high expression of *FOXA1* and *PDHX* were associated with the weak oscillator phenotype. The RAS pathway target genes *LOXL2*, *SPARC* and *CTSB* exert functions in the remodelling of the extracellular matrix, thereby favouring invasion and metastasis of tumour cells [71]–[73]. *WASF3* [74] and *FOXA1* [75] are downstream targets of the anti-apoptotic PI3K signalling pathway and have been shown to control actin polymerisation and invasion as well as differentiation of secretory gut epithelial cells, respectively. GNG11, a member of the heterotrimeric G-protein family, is involved in senescence induction via environmental stimuli [76] and has been reported recently to be deregulated in TGFIIR knock-out epithelial cells capable of increased metastasis [77]. *PPARD* and *PPARB* expression is upregulated in colorectal cancer [78] and the gene was found activated by the K-RAS pathways in rat intestinal epithelial cells [79]. The protein mediates activation of PI3K signalling via PTEN deregulation and can enhance anti-apoptotic signalling and cell survival [70]. Taken together, these observations show that targets of RAS/MAPK-dependent signalling are associated with a certain oscillator phenotype. With the exception of *LOXL2*, the RAS target genes that play a role in cancer invasion and metastasis are downregulated in the weak oscillators, thus being associated with the bad prognosis phenotype. In addition, PI3K signalling might play a functional role in clock deregulation as indicated by differential expression of *WASF3* and *FOXA1*. Recently it was shown that GSK3β is able to phosphorylate and destabilize CLOCK [80]. Overexpression of oncogenic RAS and subsequent activation of PI3K/Akt resulted in GSK3β inhibition and in an increased stabilization of CLOCK. Comparing clock gene expression between immortal and RAS transformed HaCaT cell, we also detected increased levels of *Clock* mRNA. To what extent the elevated *Clock* mRNA levels are due to RAS/MAPK or RAS/PI3K signalling in our cells needs to be investigated.

Furthermore, we correlated the set of discriminative genes found to an extended network of the mammalian circadian clock. From our assembled network it is evident that the core-clock genes directly regulate a set of 33 clock-associated genes involved in the Wnt, the TGF-β, Jak-STAT and MAPK signalling pathway. We also found many of the clock-associated genes to be involved in important biological processes such as cell cycle and repair mechanisms, immune defense, metabolism and the xenobiotics detoxification mechanisms. All these processes and pathways are often found to be deregulated in cancer. The assembled network established in this study provides a novel extension of the circadian system to output clock-associated genes which can potentially be relevant in terms of drug targets or even in the prognosis of cancer. We also suggest that a feedback from the clock-associated genes to the core-clock exists. This might explain the fact that the deregulation of cancer-related pathways can lead to perturbations of the circadian clock leading to the observed phenotype of disturbed sleep patterns as observed in cancer patients. The presented interconnections could as well help to understand why disruption of normal rhythms caused by perturbations of the clock can increase cancer incidence among night shift workers.

### RAS/MAPK can directly alter the circadian clock: Novel insights from modelling

We present in the manuscript several pieces of evidence that hint to effects of the MAPK/RAS pathway in perturbation of the clock phenotype. From the bioinformatics analysis of the extended circadian clock network, to the association of genes in the discriminative set found from the microarray analysis, there is support pointing to the MAPK/RAS pathway as an important regulator in the circadian system.

We extended our study of the connection between oncogenic pathways and the circadian clock with a model system which allowed us - in a controlled way - to explore the role of MAPK/RAS for the circadian oscillator. The starting point for the model was the comparison of the expression levels of 6 clock genes, which revealed downregulation of *Per2* and increased expression of *Cry1* and to a lesser extend of *Clock* in the RAS-transformed A5RT3 cells. Downregulation of *Per2* is in line with previous reports for colorectal and skin cancer [81], [82] indicating that *Per2* deregulation is correlated with malignant transformation in different systems. Interestingly, upregulation of *Clock* and *CKIε* expression was recently found in colorectal cancer tissues as compared to the adjacent normal mucosa and in these tissues the differential expression of *Bmal1* and *Per1* was correlated with metastasis [83]. We used a mathematical model for the mammalian circadian clock which we previously had implemented to develop a hypothesis on the mechanism of RAS/MAPK mediated alterations in the circadian clock. We hypothesised that RAS perturbation alone is sufficient to induce the observed altered phenotype, which corresponds to a deregulation in the circadian clock. This assumption could be verified experimentally. Although our mathematical model was optimized for a clock working under a normal scenario of non-pathogenic conditions, we were able to simulate the cancer phenotype observed experimentally, by perturbing a single parameter. The perturbed parameter is involved in the transcriptional regulation of clock genes, which is carried out by BMAL1 as a transcriptional activator. Published data on cells from the chick pineal gland demonstrated that activated ERK is able to phosphorylate BMAL1. This was confirmed *in vitro*, showing that p-ERK phosphorylates BMAL1 at multiple sites thereby reducing the transactivation capacity of BMAL1:CLOCK [84]. Also in other systems (not related to cancer) indirect correlations between the RAS/MAPK pathway and the circadian clock were reported [85]–[87]. Our observations in the HaCaT and A5RT3 cell lines support this data and suggest that RAS/MAPK activation perturbs the clock by interfering with clock-gene expression. Upon interference with RAS/MAPK signalling in both cells using a MEK inhibitor, we observed the opposite period phenotype as upon RAS overexpression. These results were consistent *in vitro* and *in silico*. Furthermore, using either immortal rat 208F cell lines harbouring an inducible H-Ras oncogene, or colon epithelial cells Hke3 cells harbouring an inducible K-Ras oncogene, we obtained similar results upon transient induction of oncogenic Ras. The deregulation of the circadian clock in the HKe3 cells following Ras induction suggests a stronger effect of oncogenic Ras in the colonic epithelial cells as compared to the keratinocytes, although in both cells a period lengthening phenotype can be observed. This indicates that while an influence of RAS/MAPK onto the circadian clock is visible in different cell types, we cannot easily compare different tissue types with each other. While in colorectal cancer cells the circadian clock gets strongly disrupted upon Ras induction (HKe3 clone 8) and gain of metastatic potential (SW620 cells), the metastatic keratinocytes A5RT3 still oscillate. Therefore, the RAS-mediated deregulation of clock rhythm is not necessarily connected to the metastatic potential of the cells, but can rather be considered a specific response of cells towards RAS oncogene-mediated tumourigenesis.

Together, our findings shed light into the mechanism by which the circadian clock is perturbed in carcinogenesis via a single oncogenic pathway, the RAS/MAPK pathway.

## Materials and Methods

### Text mining approach

Information about protein-protein and protein-DNA interactions were taken from our freely available GeneView repository of facts extracted from PubMed abstracts or full texts [50]. In a nutshell, protein names were recognized using the state-of-the-art tool GNAT [88] and were mapped to Entrez-Gene identifiers. All co-occurring protein citations in a sentence were classified using a custom support vector machine (SVM) kernel [89]. This SVM-kernel achieved very good results in a comprehensive evaluation of nine machine learning kernels for interaction extraction from text [90], with F1-measures ranging between 56.2 and 76.1, depending on target corpus. To account for species specificity we mapped mammalian gene identifiers to HomoloGene cluster [91]. Sentences containing potentially novel interactions were ranked by confidence of the SVM-kernel (distance to the hyperplane). In total, we extracted 15,197,637 protein pairs in 1,372,877 different PubMed articles. Of those we classified 3,921,267 (25%) as interacting. The general quality of this repository was estimated by random evaluation of 181 predicted interactions between circadian elements. From these a domain expert classified the majority of 135 (74.6%) as correct and 46 (25.4%) as incorrect. We also added to the network all relevant interactions described in the STRING database, which provides a regularly updated high quality compendium of protein-protein interactions (PPI) from several PPI-repositories, such as Kegg, Bind, Mint, and IntAct.

### Microarray experiments

#### a) Experimental setup

Cells were all trypsinized and replated on a Friday (8–9:30 am), medium was completely removed and fresh serum was added to make sure that all cells get the mitogenic stimulus at the same time. Cells were plated in 10-cm dishes at a density of 5×10^5^ cells for the 48 h time point to guarantee for constant growth over time and prevent density-dependent growth arrest. On Monday 8 a.m., cells were refed with fresh medium and serum and in cases of K-Ras-induction, the relevant substance was added. Each following day, the cells were collected after 24 hours (microarray time 0) or two days later (microarray time 48).

#### b) Analysis

Gene expression data (Affymetrix Human Gene 1.0 ST microarrays) were analyzed using the statistical programming environment R. The Affymetrix Human Gene 1.0 ST microarray contains 32,321 probesets. 21,307 probesets are associated to 19,942 distinct human genes. Samples are grouped by type (circadian/non-circadian) and time point (0 h/48 h). Quality of all arrays was determined using array QualityMetrics [92]. The arrays were subsequently normalized using robust multichip average (RMA) [93]. Differential Expression was determined by moderated t-test using R-package limma [94]. The moderated t-test was described as a robust method especially useful for small sample sizes and performed better or at least on-a-par with other commonly used statistically methods [95]–[98].

Detailed information is provided in Text S1. We found no significant difference between the time points 0 h/48 h for the same cell line (lowest Benjamin-Hochberg corrected p-value, 0.99). Consequently, these can be considered as biological replicates.

To find a set of best-discriminating genes, we followed a leave-one-out cross validation strategy. For each cell line we excluded both samples (0 and 48 hour time points) once and determined the 100 most significant probe sets, between the remaining strong/weak oscillator phenotypes. We used moderated t-test to select the most discriminative genes ranked by confidence (p-value). Following this procedure we obtained 6 lists. The quality of each list was evaluated as follows: 1) we generated a heatmap using expression profiles for all six cell lines using the current list of 100 discriminating probes. 2) For heatmaps which correctly cluster the excluded cell line into the expected group, results are retained. We found this procedure to be more robust than global comparison of all arrays, because outliers were filtered out through the intersection. This procedure was repeated for all six different cell lines. 3) Finally, we generated a discriminative list of 45 genes by taking the intersection between all useful discriminative gene-lists (Text S1). The microarray dataset was submitted to GEO with the ref: GSE46549 and will be released upon publication.

#### c) Validation

For validation purpose we normalized the cell lines using frozen Robust Multi-array Analysis fRMA, which has better characteristics when working incrementally with new batches of arrays [99]. To further reduce batch effects we replaced the cell lines CaCo2 and HCT116 with respective biological replicates. We then generated a heat map for each cell line using the previously defined 45 genes.

### Modelling data

All simulations were done using a mathematical model for the mammalian circadian clock which we previously developed [11]. The plots in Figures 6B and 7A were produced using XPPAUT, version 5.85 (http://www.math.pitt.edu/~bard/xpp/xpp.html) and the XPPAUT subsystem AUTO.

### Circadian data

Periods and amplitudes were estimated by fitting the cosine wave function via the ChronoStar analysis software [100]. For visualization, data were smoothened by a 4 hours-running average. Different basal luciferase levels from raw data were included by the fold change in luciferase activity relative to GFP controls for de-trended and smoothed data.

### Cell culture

HCT116 human colorectal carcinoma cells were maintained in McCoy's 5A culture medium. HaCaT keratinocytes and its derivatives HaCaT I7, II4 and A5RT3, the osteosarcoma U2OS, the human colon carcinoma cell lines HT-29, RKO, SW480, LIM1512, CaCo2 and the rat fibroblast cells 208F and IR2 were cultured in Dulbecco's modified medium (Gibco). HKe3 cells were cultivated in D10 with G418, HKe3 clone 8 cells in D10 with G418, Zeocin and puromycin (all Sigma). For K-Ras induction mifepristone was added at 2.5 µM. All culture media were supplemented with 10% fetal calf serum and 1% penicillin-streptomycin (Biochrom). Stable-transduced cell populations were selected and maintained in medium containing puromycin (Sigma). For live-cell bioluminescence recording (evaluation/analysis/monitoring), cells were maintained in phenol red-free DMEM (Gibco) containing 10% fetal calf serum, 1% penicillin-steptomycin and 0.1 mM D-Luciferin (PJK). Cell morphology and density were controlled by light microscopy. All cells were incubated at 37°C with 5% CO_2_ atmosphere.

### Crypt cultures

Crypt organoid cultures from *Per2* transgenic mice [101] for bioluminescence imaging were established as previously described [102]. Briefly, crypts from the proximal half of the small intestine were embedded in Matrigel (BD) at a density of app. 75 crypts/25 µl. For LumiCycle analysis 3 µl of 10 mM D-Luciferin were added per 25 µl of Matrigel with crypts and 10 drops of 28 µl volume each were plated per dish. Crypt organoids were cultured in Advanced DMEM/F12 without phenol red (Gibco/Life Technologies) supplemented with 0.1 mM D-Luciferin as well as additives and growth factors.

### Lentivirus production

Lentiviral elements containing a BMAL1-promoter-driven luciferase (BLP) were generated as previously described [103]. HEK293T cells were seeded in 175 cm^2^ culture flasks and co-transfected with 12.5 µg packaging plasmid psPAX, 7.5 µg envelope plasmid pMD2G and 17.5 µg BMAL1-promoter (BLP) luciferase expression plasmid using the CalPhos mammalian transfection kit (Clontech) according to the manufacturer's instruction. To harvest the lentiviral particles, the supernatant was centrifuged at 4100× g for 15 min to remove cell debris and passed through a 45 µm filter. The lentiviral particles were concentrated to obtain a retentate by passing the supernatant through an Amicon Ultra centrifugal filter device (Millipore). The obtained retentate, containing 100× concentrated recombinant lentivirus, was stored at −80°C.

### Transduction with lentiviral vectors

All cell lines were seeded in 12-well plates and incubated at standard cell culture conditions until they reached 80% of confluency. Culture medium was removed and cells were washed once with 1× PBS. Cells were covered with DMEM containing 8 µg/ml protamin sulphate (Sigma-Aldrich) and 300 µl of supernatant or 20× retentate (diluted in DMEM) of the corresponding lentivirus (BLP: BMAL1-promoter luciferase reporter). The next day, the medium was replaced with selection medium (DMEM supplemented 10% fetal calf serum, 1% penicillin-streptomycin and puromycin,) was added to obtain stable transduced cells and incubated at 37°C with 5% CO_2_ atmosphere.

### Synchronization and measurement of circadian rhythms

To guarantee that confluency is reached only by the end of the measurements, each cell line was specifically tested both prior to the live-cell bioluminescence experiments and then again following transduction with the lentivirus harbouring the *Bmal1* reporter.

For bioluminescence measurement, 1–5×10^5^ cells were plated in 35 mm dishes or 1×10^4^ cells in 96-well plates and synchronized by a single pulse of 1 µM dexamethasone (Sigma) for 1 hour. Next, cells were washed once with 1× PBS and cultured with phenol-red-free DMEM supplemented with 0.1 mM D-Luciferin and puromycin or hygromycin. *Bmal1*-promoter-(BLP)-reporter activity was measured, using a photomultiplier tube (PMT)-based system (Hamamatsu Photonics) in a LumiCycle instrument (Actimetrics) or in a plate luminometer (TopCount equipment, Perkin Elmer). Raw luminescence data were de-trended by dividing luminescence counts by the 24 hours running average. Phase, period and amplitude were analysed using the ChronoStar analysis software [100].

### Temperature entrainment

1–5×10^5^ cells were plated in 35 mm dishes and synchronized with 1 µM dexamethasone for 1 hour. Subsequently, cells were washed once with 1× PBS and cultured with phenol red-free DMEM supplemented with 0.1 mM luciferin and puromycin. Cells were subsequently entrained to temperature cycles (5 d: 10 hours 37°C constant; 2 hours 37°C ramp; 33°C constant; 2 hours 33°C ramp; 37°C constant). BLP-reporter activity was measured in the PMT-based system. Detrending of the time series was performed by dividing the luminescence counts by 24 hours running averages. Phase, period and amplitude were analysed using the ChronoStar analysis software [100].

### RNA isolation

Cells were seeded in 35 mm dishes with a density of 1–5×10^5^ cells/dish and synchronized for 1 hour with dexamethasone. 24 hours later, cells were collected every three hours and total RNA was extracted. The measurement was started 24 hours after synchronization to avoid an influence of the immediate early gene response that may not reflect an accurate result of the oscillator. Total RNA was isolated using the RNeasy extraction kit (Qiagen), including DNAse digestion, according to manufacturer's instructions. Cells were lysed with 350 µl RLT buffer (with 1% β-mercaptoethanol) and lysate was homogenized with a syringe. Total RNA concentration was determined by OD_260_ using an Ultrospec 3000 UV spectrophotometer and stored at −80°C.

### cDNA synthesis and quantitative real-time PCR

Total RNA was isolated using the RNeasy extraction kit (Qiagen), according to manufacturer's instructions. Total RNA concentration was determined by OD_260_ using an UV spectrophotometer Ultrospec 3000. 1 µg of total RNA was reverse-transcribed to cDNA with 2 U/µl RevertAid M-MuLV reverse transcriptase and 500 µM random 15-mer primers (Fermentas).

For quantitative SYBR-green based real-time PCR we used 20 ng of cDNA and the commercial primers from QuantiTect primer assays for *Gapdh, Bmal1, Per1, Per2, Rorα, Cry1, Cry2 and Clock* (Qiagen). For each primer, a non-template control (NTC) was always included. The reaction was performed in an ABI Prism 7000 SDS thermocycler (Applied Biosystems). The obtained data were analysed using the ABI Prism 7000 System SDS software (Applied Biosystems). Each sample was measured in triplicates. The expression levels were normalized to those of *Gapdh* (ΔCT) and calibrated to the value at time 0 of the control cell line (ΔΔCT). The relative quantification was performed using the 2_−ΔΔCT_ method.

### Western blot

Total protein lysates for immunoblotting were obtained using SDS lysisbuffer (10 mM Tris-HCl [pH 7.5], 1% SDS, and 2 mM EDTA) supplemented with protease inhibitors (1 mM PMSF, 50 mM NaF, 50 µg/ml leupeptin, 1 mM orthovanadate, 4 µg/ml aprotinin). Protein concentration was determined by the Pierce BCA Protein Assay Kit (Thermo Scientific #23227F). 30 µg of protein were resolved by SDS-PAGE and transferred to a nitrocellulose membrane. Antibodies used were phospho-ERK1/2 (Thr202/Y204; Cell Signaling #4370), Ras (Thermo Scientific #1862335) and GAPDH (Ambion Am4300). Secondary antibodies were IRDye 800CW goat anti-rabbit IgG (LI-COR Bioscience 926-32211), IRDye 800CW goat anti-mouse IgG (LI-COR Bioscience 92632210) and IRDye 680 goat anti-mouse IgG (LI-COR Bioscience 926-322220). Blots were developed using the LI-COR Odyssey system.

### MEK inhibition

1–5×10^5^ cells were plated in 35 mm dishes and cultured overnight. The next day the cells were synchronized by a single pulse of 1 µM dexamethasone (Sigma) for 1 hour after which a MEK inhibitor (UO126 final concentrations between 5 and 20 µM, Promega) was added to the culture and incubated for 7 days. Circadian rhythmicity was determined as described above.

## Supporting Information

Figure S1 Network of interaction correlating all three sets of genes. In total 646 Interactions with 13,226 individual evidences (sentences) were found to be involved in the assembly of the network. Cancer-related genes are represented in orange, clock genes in blue, discriminative genes in green and common genes in violet (e.g. TNFα). Grey lines represent text-mined interactions, blue lines interactions from the STRING database, and yellow lines common interactions.
(EPS)

Figure S2 A comprehensive regulatory network of the mammalian circadian clock. We used a curated text mining approach to search for interactions between our genes of interest. The network represents 108 novel interactions, supported by 132 PubMed references. In the core of the network (orange circles) the main components of the canonical feedback loops are shown. The outer shell of the network (grey circles) shows the clock-regulated genes and proteins feeding back to the core components and thereby potentially influencing the oscillations. Red lines represent inhibitory interactions; green lines, activating interactions; grey lines, other kinds of interactions.
(EPS)

Figure S3 Networks of circadian regulation for the discriminative genes. All networks were generated using the GeneView software with additional data retrieved from the STRING database. Network of interaction correlating circadian clock genes and discriminative genes. Clock genes are represented in blue, discriminative genes in green and common genes in violet. Grey lines represent text-mined interactions, blue lines interactions from the STRING database, and yellow lines common interactions.
(EPS)

Figure S4 Networks of cancer regulation for the discriminative genes. All networks were generated using the GeneView software with additional data retrieved from the STRING database. Network of interaction correlating discriminative genes and cancer genes. Cancer-related genes are represented in orange, clock genes in blue, discriminative genes in green and common genes in violet (e.g. TNFα). Grey lines represent text-mined interactions, blue lines interactions from the STRING database, and yellow lines common interactions.
(EPS)

Figure S5 Clock phenotypes of different cell lines. Cells were lentivirally transduced with a *Bmal1*-luciferase reporter construct and bioluminescence was measured over 6 days. Given are detrended time series (black line) (**A**) human osteosarcoma cell line (U2OS). (**B–H**) Human colon cancer cell lines; blue - strong oscillators (HKe3, HCT116); red - weak oscillators (SW403, SW620, Colo205, CaCo2). (**I**) Colon crypts derived from PER2:LUC transgenic mice [101].
(EPS)

Figure S6 Western blot analysis for RAS and phosphorylated ERK in 208F and IR2 cells following IPTG induction. Rat immortal fibroblast 208F and the IR2 derivatives harbouring a conditional H-Ras oncogene were treated with 10 µM IPTG for the time indicated. Extracts were prepared from untreated cells (−), from cells treated with the solvent DMSO (c) or from cells treated with 10 µM IPTG (10). Levels of RAS and phosphorylated ERK were analysed via Western Blot.
(EPS)

Figure S7 Effect of RAS/MAPK inhibition in HaCaT and A5RT3 cell lines. Shown are the results from 3 independent experiments. (**A**) HaCaT have a period of 24.6±0.26 hours (p<0.05, Student's *t*-test), (**B**) HaCaT cells treated with UO126 (15 µM) show a period decrease (23.3±0.49 hours p<0.05, Student's *t*-test). (**C**) HaCaT A5RT3 present a larger period of 26.2±0.05 hours (p<0.05, Student's *t*-test). (**D**) A5RT3 cells treated with UO126 (5 µM) show a period decrease (23.5±0.02 hours p<0.05, Student's *t*-test). (**E**) Overlap of panels (A–D). (**F**) HaCaT cells and A5RT3 cells where treated with 5, 10, 15 or 20 µm U0126 to inhibit MEK. Control extracts where prepared from cells left untreated (−), or from cells treated with the solvent DMSO in the appropriate concentration (c). Levels of RAS and phosphorylated ERK were analysed. GAPDH was used as loading control in both cases.
(EPS)

Table S1 List of interactions represented in the networks of Figure 4 and corresponding references.
(XLS)

Table S2 Colon cancer-specific interactions retrieved by text-mining.
(XLS)

Text S1 Identification of a list of best discriminative genes: cross-validation procedure.
(DOC)

Text S2 A comprehensive regulatory network for the mammalian circadian clock.
(DOC)

Text S3 Analysis of circadian expression data for the genes of interest indicates enrichment in circadian genes.
(DOC)

Text S4 Statistical significance of the 45-discriminative-gene list.
(DOC)

## References

[1] Young MW, Kay SA (2001) Time zones: a comparative genetics of circadian clocks. Nat Rev Genet 2: 702–715. 11533719 10.1038/35088576

[2] Bell-Pedersen D, Cassone VM, Earnest DJ, Golden SS, Hardin PE, et al (2005) Circadian rhythms from multiple oscillators: lessons from diverse organisms. Nat Rev Genet 6: 544–556. 15951747 10.1038/nrg1633 PMC2735866

[3] Panda S, Antoch MP, Miller BH, Su AI, Schook AB, et al (2002) Coordinated transcription of key pathways in the mouse by the circadian clock. Cell 109: 307–320. 12015981 10.1016/s0092-8674(02)00722-5

[4] Hankins MW, Peirson SN, Foster RG (2008) Melanopsin: an exciting photopigment. Trends Neurosci 31: 27–36. 18054803 10.1016/j.tins.2007.11.002

[5] Stokkan KA, Yamazaki S, Tei H, Sakaki Y, Menaker M (2001) Entrainment of the circadian clock in the liver by feeding. Science 291: 490–493. 11161204 10.1126/science.291.5503.490

[6] Suter DM, Schibler U (2009) Physiology. Feeding the clock. Science 326: 378–379. 19833950 10.1126/science.1181278

[7] Albrecht U (2012) Timing to perfection: the biology of central and peripheral circadian clocks. Neuron 74: 246–260. 22542179 10.1016/j.neuron.2012.04.006

[8] Takahashi JS, Shimomura K, Kumar V (2008) Searching for genes underlying behavior: lessons from circadian rhythms. Science 322: 909–912. 18988844 10.1126/science.1158822 PMC3744585

[9] Brown SA, Kowalska E, Dallmann R (2012) (Re)inventing the circadian feedback loop. Dev Cell 22: 477–487. 22421040 10.1016/j.devcel.2012.02.007

[10] Ueda HR, Hayashi S, Chen W, Sano M, Machida M, et al (2005) System-level identification of transcriptional circuits underlying mammalian circadian clocks. Nat Genet 37: 187–192. 15665827 10.1038/ng1504

[11] Relogio A, Westermark PO, Wallach T, Schellenberg K, Kramer A, et al (2011) Tuning the mammalian circadian clock: robust synergy of two loops. PLoS Comput Biol 7: e1002309. 22194677 10.1371/journal.pcbi.1002309 PMC3240597

[12] Wallach T, Schellenberg K, Maier B, Kalathur RK, Porras P, et al (2013) Dynamic circadian protein-protein interaction networks predict temporal organization of cellular functions. PLoS Genet 9: e1003398. 23555304 10.1371/journal.pgen.1003398 PMC3610820

[13] Bozek K, Relogio A, Kielbasa SM, Heine M, Dame C, et al (2009) Regulation of clock-controlled genes in mammals. PLoS One 4: e4882. 19287494 10.1371/journal.pone.0004882 PMC2654074

[14] Bass J (2012) Circadian topology of metabolism. Nature 491: 348–356. 23151577 10.1038/nature11704

[15] Masri S, Zocchi L, Katada S, Mora E, Sassone-Corsi P (2012) The circadian clock transcriptional complex: metabolic feedback intersects with epigenetic control. Ann N Y Acad Sci 1264: 103–109. 22834651 10.1111/j.1749-6632.2012.06649.x PMC3464365

[16] Takahashi JS, Hong HK, Ko CH, McDearmon EL (2008) The genetics of mammalian circadian order and disorder: implications for physiology and disease. Nat Rev Genet 9: 764–775. 18802415 10.1038/nrg2430 PMC3758473

[17] Sahar S, Sassone-Corsi P (2009) Metabolism and cancer: the circadian clock connection. Nat Rev Cancer 9: 886–896. 19935677 10.1038/nrc2747

[18] Matsuo T, Yamaguchi S, Mitsui S, Emi A, Shimoda F, et al (2003) Control mechanism of the circadian clock for timing of cell division in vivo. Science 302: 255–259. 12934012 10.1126/science.1086271

[19] Kondratova AA, Kondratov RV (2012) The circadian clock and pathology of the ageing brain. Nat Rev Neurosci 13: 325–335. 22395806 10.1038/nrn3208 PMC3718301

[20] Fu L, Lee CC (2003) The circadian clock: pacemaker and tumour suppressor. Nat Rev Cancer 3: 350–361. 12724733 10.1038/nrc1072

[21] Rafnsson V, Tulinius H, Jonasson JG, Hrafnkelsson J (2001) Risk of breast cancer in female flight attendants: a population-based study (Iceland). Cancer Causes Control 12: 95–101. 11246849 10.1023/a:1008983416836

[22] Kubo T, Ozasa K, Mikami K, Wakai K, Fujino Y, et al (2006) Prospective cohort study of the risk of prostate cancer among rotating-shift workers: findings from the Japan collaborative cohort study. Am J Epidemiol 164: 549–555. 16829554 10.1093/aje/kwj232

[23] Mormont MC, Waterhouse J, Bleuzen P, Giacchetti S, Jami A, et al (2000) Marked 24-h rest/activity rhythms are associated with better quality of life, better response, and longer survival in patients with metastatic colorectal cancer and good performance status. Clin Cancer Res 6: 3038–3045. 10955782

[24] Filipski E, Levi F (2009) Circadian disruption in experimental cancer processes. Integr Cancer Ther 8: 298–302. 20042408 10.1177/1534735409352085

[25] Levi F, Okyar A, Dulong S, Innominato PF, Clairambault J (2010) Circadian timing in cancer treatments. Annu Rev Pharmacol Toxicol 50: 377–421. 20055686 10.1146/annurev.pharmtox.48.113006.094626

[26] Gery S, Komatsu N, Baldjyan L, Yu A, Koo D, et al (2006) The circadian gene per1 plays an important role in cell growth and DNA damage control in human cancer cells. Mol Cell 22: 375–382. 16678109 10.1016/j.molcel.2006.03.038

[27] Hoffman AE, Zheng T, Stevens RG, Ba Y, Zhang Y, et al (2009) Clock-cancer connection in non-Hodgkin's lymphoma: a genetic association study and pathway analysis of the circadian gene cryptochrome 2. Cancer Res 69: 3605–3613. 19318546 10.1158/0008-5472.CAN-08-4572 PMC3175639

[28] Fu L, Pelicano H, Liu J, Huang P, Lee C (2002) The circadian gene Period2 plays an important role in tumor suppression and DNA damage response in vivo. Cell 111: 41–50. 12372299 10.1016/s0092-8674(02)00961-3

[29] Lis CG, Grutsch JF, Wood P, You M, Rich I, et al (2003) Circadian timing in cancer treatment: the biological foundation for an integrative approach. Integr Cancer Ther 2: 105–111. 15035897 10.1177/1534735403002002002

[30] Levi F, Schibler U (2007) Circadian rhythms: mechanisms and therapeutic implications. Annu Rev Pharmacol Toxicol 47: 593–628. 17209800 10.1146/annurev.pharmtox.47.120505.105208

[31] Bernard S, Cajavec Bernard B, Levi F, Herzel H (2010) Tumor growth rate determines the timing of optimal chronomodulated treatment schedules. PLoS Comput Biol 6: e1000712. 20333244 10.1371/journal.pcbi.1000712 PMC2841621

[32] Hrushesky WJ, Grutsch J, Wood P, Yang X, Oh EY, et al (2009) Circadian clock manipulation for cancer prevention and control and the relief of cancer symptoms. Integr Cancer Ther 8: 387–397. 19926611 10.1177/1534735409352086

[33] Innominato PF, Levi FA, Bjarnason GA (2010) Chronotherapy and the molecular clock: Clinical implications in oncology. Adv Drug Deliv Rev 62: 979–1001. 20600409 10.1016/j.addr.2010.06.002

[34] Gaddameedhi S, Reardon JT, Ye R, Ozturk N, Sancar A (2012) Effect of circadian clock mutations on DNA damage response in mammalian cells. Cell Cycle 11: 3481–3491. 22918252 10.4161/cc.21771 PMC3466558

[35] Sancar A, Lindsey-Boltz LA, Kang TH, Reardon JT, Lee JH, et al (2010) Circadian clock control of the cellular response to DNA damage. FEBS Lett 584: 2618–2625. 20227409 10.1016/j.febslet.2010.03.017 PMC2878924

[36] Kowalska E, Ripperger JA, Hoegger DC, Bruegger P, Buch T, et al (2012) NONO couples the circadian clock to the cell cycle. Proc Natl Acad Sci U S A 110: 1592–9. 23267082 10.1073/pnas.1213317110 PMC3562797

[37] Borgs L, Beukelaers P, Vandenbosch R, Belachew S, Nguyen L, et al (2009) Cell “circadian” cycle: new role for mammalian core clock genes. Cell Cycle 8: 832–837. 19221497 10.4161/cc.8.6.7869

[38] Wood PA, Du-Quiton J, You S, Hrushesky WJ (2006) Circadian clock coordinates cancer cell cycle progression, thymidylate synthase, and 5-fluorouracil therapeutic index. Mol Cancer Ther 5: 2023–2033. 16928823 10.1158/1535-7163.MCT-06-0177

[39] Nakahata Y, Sahar S, Astarita G, Kaluzova M, Sassone-Corsi P (2009) Circadian control of the NAD+ salvage pathway by CLOCK-SIRT1. Science 324: 654–657. 19286518 10.1126/science.1170803 PMC6501775

[40] Brooks CL, Gu W (2009) How does SIRT1 affect metabolism, senescence and cancer? Nat Rev Cancer 9: 123–128. 19132007 10.1038/nrc2562 PMC2857763

[41] Kondratov RV, Antoch MP (2007) Circadian proteins in the regulation of cell cycle and genotoxic stress responses. Trends Cell Biol 17: 311–317. 17644383 10.1016/j.tcb.2007.07.001

[42] Chen ST, Choo KB, Hou MF, Yeh KT, Kuo SJ, et al (2005) Deregulated expression of the PER1, PER2 and PER3 genes in breast cancers. Carcinogenesis 26: 1241–1246. 15790588 10.1093/carcin/bgi075

[43] Hua H, Wang Y, Wan C, Liu Y, Zhu B, et al (2006) Circadian gene mPer2 overexpression induces cancer cell apoptosis. Cancer Sci 97: 589–596. 16827798 10.1111/j.1349-7006.2006.00225.x PMC2662332

[44] Chen-Goodspeed M, Lee CC (2007) Tumor suppression and circadian function. J Biol Rhythms 22: 291–298. 17660446 10.1177/0748730407303387

[45] Yang WS, Stockwell BR (2008) Inhibition of casein kinase 1-epsilon induces cancer-cell-selective, PERIOD2-dependent growth arrest. Genome Biol 9: R92. 18518968 10.1186/gb-2008-9-6-r92 PMC2481424

[46] Yi CH, Zheng T, Leaderer D, Hoffman A, Zhu Y (2009) Cancer-related transcriptional targets of the circadian gene NPAS2 identified by genome-wide ChIP-on-chip analysis. Cancer Lett 284: 149–156. 19457610 10.1016/j.canlet.2009.04.017 PMC3182267

[47] Alhopuro P, Bjorklund M, Sammalkorpi H, Turunen M, Tuupanen S, et al (2010) Mutations in the circadian gene CLOCK in colorectal cancer. Mol Cancer Res 8: 952–960. 20551151 10.1158/1541-7786.MCR-10-0086

[48] Levi F, Filipski E, Iurisci I, Li XM, Innominato P (2007) Cross-talks between circadian timing system and cell division cycle determine cancer biology and therapeutics. Cold Spring Harb Symp Quant Biol 72: 465–475. 18419306 10.1101/sqb.2007.72.030

[49] Zhang EE, Liu AC, Hirota T, Miraglia LJ, Welch G, et al (2009) A genome-wide RNAi screen for modifiers of the circadian clock in human cells. Cell 139: 199–210. 19765810 10.1016/j.cell.2009.08.031 PMC2777987

[50] Thomas P, Starlinger J, Vowinkel A, Arzt S, Leser U (2012) GeneView: a comprehensive semantic search engine for PubMed. Nucleic Acids Res 40: W585–591. 22693219 10.1093/nar/gks563 PMC3394277

[51] Walther A, Johnstone E, Swanton C, Midgley R, Tomlinson I, et al (2009) Genetic prognostic and predictive markers in colorectal cancer. Nat Rev Cancer 9: 489–499. 19536109 10.1038/nrc2645

[52] Szklarczyk D, Franceschini A, Kuhn M, Simonovic M, Roth A, et al (2010) The STRING database in 2011: functional interaction networks of proteins, globally integrated and scored. Nucleic Acids Res 39: D561–568. 21045058 10.1093/nar/gkq973 PMC3013807

[53] Schroder K, Hertzog PJ, Ravasi T, Hume DA (2004) Interferon-gamma: an overview of signals, mechanisms and functions. J Leukoc Biol 75: 163–189. 14525967 10.1189/jlb.0603252

[54] Fusenig NE, Boukamp P (1998) Multiple stages and genetic alterations in immortalization, malignant transformation, and tumor progression of human skin keratinocytes. Mol Carcinog 23: 144–158. 9833775 10.1002/(sici)1098-2744(199811)23:3<144::aid-mc3>3.0.co;2-u

[55] Liu HS, Scrable H, Villaret DB, Lieberman MA, Stambrook PJ (1992) Control of Ha-ras-mediated mammalian cell transformation by Escherichia coli regulatory elements. Cancer Res 52: 983–989. 1737361

[56] Shirasawa S, Furuse M, Yokoyama N, Sasazuki T (1993) Altered growth of human colon cancer cell lines disrupted at activated Ki-ras. Science 260: 85–88. 8465203 10.1126/science.8465203

[57] Shi SQ, Ansari TS, McGuinness OP, Wasserman DH, Johnson CH (2013) Circadian disruption leads to insulin resistance and obesity. Curr Biol 23: 372–381. 23434278 10.1016/j.cub.2013.01.048 PMC3595381

[58] Kondratov RV, Kondratova AA, Gorbacheva VY, Vykhovanets OV, Antoch MP (2006) Early aging and age-related pathologies in mice deficient in BMAL1, the core componentof the circadian clock. Genes Dev 20: 1868–1873. 16847346 10.1101/gad.1432206 PMC1522083

[59] Janich P, Pascual G, Merlos-Suarez A, Batlle E, Ripperger J, et al (2011) The circadian molecular clock creates epidermal stem cell heterogeneity. Nature 480: 209–214. 22080954 10.1038/nature10649

[60] Ballesta A, Dulong S, Abbara C, Cohen B, Okyar A, et al (2011) A combined experimental and mathematical approach for molecular-based optimization of irinotecan circadian delivery. PLoS Comput Biol 7: e1002143. 21931543 10.1371/journal.pcbi.1002143 PMC3169519

[61] Li Q, Wang X, Lu Z, Zhang B, Guan Z, et al (2010) Polycomb CBX7 directly controls trimethylation of histone H3 at lysine 9 at the p16 locus. PLoS One 5: e13732. 21060834 10.1371/journal.pone.0013732 PMC2966406

[62] Kim MS, Chung NG, Kang MR, Yoo NJ, Lee SH (2011) Genetic and expressional alterations of CHD genes in gastric and colorectal cancers. Histopathology 58: 660–668. 21447119 10.1111/j.1365-2559.2011.03819.x

[63] Wu S, Shi Y, Mulligan P, Gay F, Landry J, et al (2007) A YY1-INO80 complex regulates genomic stability through homologous recombination-based repair. Nat Struct Mol Biol 14: 1165–1172. 18026119 10.1038/nsmb1332 PMC2754171

[64] Majumder P, Gomez JA, Chadwick BP, Boss JM (2008) The insulator factor CTCF controls MHC class II gene expression and is required for the formation of long-distance chromatin interactions. J Exp Med 205: 785–798. 18347100 10.1084/jem.20071843 PMC2292219

[65] Majumder P, Boss JM (2010) CTCF controls expression and chromatin architecture of the human major histocompatibility complex class II locus. Mol Cell Biol 30: 4211–4223. 20584980 10.1128/MCB.00327-10 PMC2937552

[66] Merkenschlager M, Odom DT (2013) CTCF and Cohesin: Linking Gene Regulatory Elements with Their Targets. Cell 152: 1285–1297. 23498937 10.1016/j.cell.2013.02.029

[67] Sassone-Corsi P (2013) Physiology. When metabolism and epigenetics converge. Science 339: 148–150. 23307727 10.1126/science.1233423

[68] Sassone-Corsi P (2012) Minireview: NAD+, a circadian metabolite with an epigenetic twist. Endocrinology 153: 1–5. 22186411 10.1210/en.2011-1535 PMC3249676

[69] Feng D, Lazar MA (2012) Clocks, metabolism, and the epigenome. Mol Cell 47: 158–167. 22841001 10.1016/j.molcel.2012.06.026 PMC3408602

[70] Park JI, Kwak JY (2012) The role of peroxisome proliferator-activated receptors in colorectal cancer. PPAR Res 2012: 876418. 23024650 10.1155/2012/876418 PMC3447370

[71] Bradshaw AD (2012) Diverse biological functions of the SPARC family of proteins. Int J Biochem Cell Biol 44: 480–488. 22249026 10.1016/j.biocel.2011.12.021 PMC3312742

[72] Mohamed MM, Sloane BF (2006) Cysteine cathepsins: multifunctional enzymes in cancer. Nat Rev Cancer 6: 764–775. 16990854 10.1038/nrc1949

[73] Cano A, Santamaria PG, Moreno-Bueno G (2012) LOXL2 in epithelial cell plasticity and tumor progression. Future Oncol 8: 1095–1108. 23030485 10.2217/fon.12.105

[74] Sossey-Alaoui K, Li X, Ranalli TA, Cowell JK (2005) WAVE3-mediated cell migration and lamellipodia formation are regulated downstream of phosphatidylinositol 3-kinase. J Biol Chem 280: 21748–21755. 15826941 10.1074/jbc.M500503200

[75] Ye DZ, Kaestner KH (2009) Foxa1 and Foxa2 control the differentiation of goblet and enteroendocrine L- and D-cells in mice. Gastroenterology 137: 2052–2062. 19737569 10.1053/j.gastro.2009.08.059 PMC2789913

[76] Hossain MN, Sakemura R, Fujii M, Ayusawa D (2006) G-protein gamma subunit GNG11 strongly regulates cellular senescence. Biochem Biophys Res Commun 351: 645–650. 17092487 10.1016/j.bbrc.2006.10.112

[77] Matise LA, Palmer TD, Ashby WJ, Nashabi A, Chytil A, et al (2012) Lack of transforming growth factor-beta signaling promotes collective cancer cell invasion through tumor-stromal crosstalk. Breast Cancer Res 14: R98. 22748014 10.1186/bcr3217 PMC3680921

[78] Harman FS, Nicol CJ, Marin HE, Ward JM, Gonzalez FJ, et al (2004) Peroxisome proliferator-activated receptor-delta attenuates colon carcinogenesis. Nat Med 10: 481–483. 15048110 10.1038/nm1026

[79] Luo F, Brooks DG, Ye H, Hamoudi R, Poulogiannis G, et al (2009) Mutated K-ras(Asp12) promotes tumourigenesis in Apc(Min) mice more in the large than the small intestines, with synergistic effects between K-ras and Wnt pathways. Int J Exp Pathol 90: 558–574. 19765110 10.1111/j.1365-2613.2009.00667.x PMC2768154

[80] Spengler ML, Kuropatwinski KK, Schumer M, Antoch MP (2009) A serine cluster mediates BMAL1-dependent CLOCK phosphorylation and degradation. Cell Cycle 8: 4138–4146. 19946213 10.4161/cc.8.24.10273 PMC4073639

[81] Oshima T, Takenoshita S, Akaike M, Kunisaki C, Fujii S, et al (2011) Expression of circadian genes correlates with liver metastasis and outcomes in colorectal cancer. Oncol Rep 25: 1439–1446. 21380491 10.3892/or.2011.1207

[82] Lengyel Z, Lovig C, Kommedal S, Keszthelyi R, Szekeres G, et al (2012) Altered expression patterns of clock gene mRNAs and clock proteins in human skin tumors. Tumour Biol 34: 811–819. 23242607 10.1007/s13277-012-0611-0

[83] Oshima T, Takenoshita S, Akaike M, Kunisaki C, Fujii S, et al Expression of circadian genes correlates with liver metastasis and outcomes in colorectal cancer. Oncol Rep 25: 1439–1446. 21380491 10.3892/or.2011.1207

[84] Sanada K, Okano T, Fukada Y (2002) Mitogen-activated protein kinase phosphorylates and negatively regulates basic helix-loop-helix-PAS transcription factor BMAL1. J Biol Chem 277: 267–271. 11687575 10.1074/jbc.M107850200

[85] Williams JA, Su HS, Bernards A, Field J, Sehgal A (2001) A circadian output in Drosophila mediated by neurofibromatosis-1 and Ras/MAPK. Science 293: 2251–2256. 11567138 10.1126/science.1063097

[86] Weber F, Hung HC, Maurer C, Kay SA (2006) Second messenger and Ras/MAPK signalling pathways regulate CLOCK/CYCLE-dependent transcription. J Neurochem 98: 248–257. 16805811 10.1111/j.1471-4159.2006.03865.x

[87] Nomura K, Takeuchi Y, Fukunaga K (2006) MAP kinase additively activates the mouse Per1 gene promoter with CaM kinase II. Brain Res 1118: 25–33. 17020748 10.1016/j.brainres.2006.08.087

[88] Hakenberg J, Gerner M, Haeussler M, Solt I, Plake C, et al (2011) The GNAT library for local and remote gene mention normalization. Bioinformatics 27: 2769–2771. 21813477 10.1093/bioinformatics/btr455 PMC3179658

[89] Airola A, Pyysalo S, Bjorne J, Pahikkala T, Ginter F, et al (2008) All-paths graph kernel for protein-protein interaction extraction with evaluation of cross-corpus learning. BMC Bioinformatics 9 Suppl 11: S2. 19025688 10.1186/1471-2105-9-S11-S2 PMC2586751

[90] Tikk D, Thomas P, Palaga P, Hakenberg J, Leser U (2010) A comprehensive benchmark of kernel methods to extract protein-protein interactions from literature. PLoS Comput Biol 6: e1000837. 20617200 10.1371/journal.pcbi.1000837 PMC2895635

[91] Sayers EW, Barrett T, Benson DA, Bolton E, Bryant SH, et al (2011) Database resources of the National Center for Biotechnology Information. Nucleic Acids Res 40: D13–25. 22140104 10.1093/nar/gkr1184 PMC3245031

[92] Kauffmann A, Gentleman R, Huber W (2009) arrayQualityMetrics–a bioconductor package for quality assessment of microarray data. Bioinformatics 25: 415–416. 19106121 10.1093/bioinformatics/btn647 PMC2639074

[93] Irizarry RA, Bolstad BM, Collin F, Cope LM, Hobbs B, et al (2003) Summaries of Affymetrix GeneChip probe level data. Nucleic Acids Res 31: e15. 12582260 10.1093/nar/gng015 PMC150247

[94] Smyth GK (2004) Linear models and empirical bayes methods for assessing differential expression in microarray experiments. Stat Appl Genet Mol Biol 3: Article3. 16646809 10.2202/1544-6115.1027

[95] Jeanmougin M, de Reynies A, Marisa L, Paccard C, Nuel G, et al (2010) Should we abandon the t-test in the analysis of gene expression microarray data: a comparison of variance modeling strategies. PLoS One 5: e12336. 20838429 10.1371/journal.pone.0012336 PMC2933223

[96] Kooperberg C, Aragaki A, Strand AD, Olson JM (2005) Significance testing for small microarray experiments. Stat Med 24: 2281–2298. 15889452 10.1002/sim.2109

[97] Murie C, Woody O, Lee AY, Nadon R (2009) Comparison of small n statistical tests of differential expression applied to microarrays. BMC Bioinformatics 10: 45. 19192265 10.1186/1471-2105-10-45 PMC2674054

[98] Jeffery IB, Higgins DG, Culhane AC (2006) Comparison and evaluation of methods for generating differentially expressed gene lists from microarray data. BMC Bioinformatics 7: 359. 16872483 10.1186/1471-2105-7-359 PMC1544358

[99] McCall MN, Jaffee HA, Irizarry RA (2012) fRMA ST: frozen robust multiarray analysis for Affymetrix Exon and Gene ST arrays. Bioinformatics 28: 3153–3154. 23044545 10.1093/bioinformatics/bts588 PMC3509489

[100] Sporl F, Schellenberg K, Blatt T, Wenck H, Wittern KP, et al (2010) A circadian clock in HaCaT keratinocytes. J Invest Dermatol 131: 338–348. 20962856 10.1038/jid.2010.315

[101] Yoo SH, Yamazaki S, Lowrey PL, Shimomura K, Ko CH, et al (2004) PERIOD2::LUCIFERASE real-time reporting of circadian dynamics reveals persistent circadian oscillations in mouse peripheral tissues. Proc Natl Acad Sci U S A 101: 5339–5346. 14963227 10.1073/pnas.0308709101 PMC397382

[102] Sato T, Vries RG, Snippert HJ, van de Wetering M, Barker N, et al (2009) Single Lgr5 stem cells build crypt-villus structures in vitro without a mesenchymal niche. Nature 459: 262–265. 19329995 10.1038/nature07935

[103] Brown SA, Fleury-Olela F, Nagoshi E, Hauser C, Juge C, et al (2005) The period length of fibroblast circadian gene expression varies widely among human individuals. PLoS Biol 3: e338. 16167846 10.1371/journal.pbio.0030338 PMC1233413

