# Supplementary material for: Ras-Mediated Deregulation of the Circadian Clock in Cancer
Source: PLoS Genet. 2014 May 29;10(5):e1004338. doi: 10.1371/journal.pgen.1004338 (PMC4038477; doi:10.1371/journal.pgen.1004338)
Supplement: Text S1 — Identification of a list of best discriminative genes: cross-validation procedure. (DOC) [file pgen.1004338.s010.doc]

**Text S1 – Identification of a list of best discriminative genes: cross-validation procedure.**

**Quality control of microarrays**

A quality control procedure was carried out in all arrays. For array quality control we used the bioconductor package arrayQualityMetrics [1]. A total of six quality tests were performed (Figures 1 – 12). Arrays which failed more than two tests were not used for subsequent analysis. The outlier detection criteria are explained below in the respective sections. Arrays that were called outliers by at least one criterion are marked in Table 1, where the overall performance of the arrays is listed.

| **array** | **[*1](http://www2.informatik.hu-berlin.de/~thomas/qcFinal/#hm)** | **[*2](http://www2.informatik.hu-berlin.de/~thomas/qcFinal/#box)** | **[*3](http://www2.informatik.hu-berlin.de/~thomas/qcFinal/#rle)** | **[*4](http://www2.informatik.hu-berlin.de/~thomas/qcFinal/#nuse)** | **[*5](http://www2.informatik.hu-berlin.de/~thomas/qcFinal/#ma)** | **[*6](http://www2.informatik.hu-berlin.de/~thomas/qcFinal/#spm)** | **Cell line** | **Time point**  **[hours]** |
| --- | --- | --- | --- | --- | --- | --- | --- | --- |
| **1** |  |  |  | x | x |  | U2OS | 0 |
| **2** |  |  |  |  |  | x | HCT116 | 0 |
| **3** |  |  |  |  |  |  | RKO | 0 |
| **4** |  |  | x |  |  |  | RKO | 48 |
| **5** |  |  |  |  |  |  | Caco | 0 |
| **6** |  |  |  |  |  |  | Caco | 48 |
| **7** |  |  |  |  | x |  | HCT116 | 48 |
| **8** |  |  |  |  | x |  | LIM1215 | 0 |
| **9** |  |  |  |  |  |  | LIM1215 | 48 |
| **10** |  |  |  |  |  |  | HT29 | 0 |
| **11** |  |  |  |  | x |  | HT29 | 48 |
| **12** |  |  |  |  | x |  | SW480 | 0 |
| **13** |  |  |  |  | x |  | SW480 | 48 |

[**Table1. Array metadata and outlier detection overview**](javascript: toggle('arraymetadata'))**.** The columns with * indicate the calls from the different outlier detection methods: 1 - outlier detection by Distances between arrays; 2 - outlier detection by [Boxplots](http://www2.informatik.hu-berlin.de/~thomas/qcFinal/#box); 3 - outlier detection by Relative Log Expression (RLE); 4 - outlier detection by Normalized Unscaled Standard Error (NUSE); 5 - outlier detection by MA plots; 6 - outlier detection by Spatial distribution of M.

## 1 – Outlier detection by distance between arrays


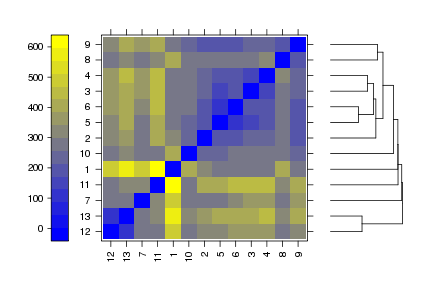


**Figure 1.** False colour heatmap of the distances between arrays. The colour scale is chosen to cover the range of distances encountered in the dataset. Patterns in this plot can indicate clustering of the arrays either because of intended biological or unintended experimental factors (batch effects). The distance *d_ab_* between two arrays *a* and *b* is computed as the mean absolute difference (L_1_-distance) between the data of the arrays (using the data from all probes without filtering). In formula, *d_ab_* = mean | *M_ai_ - M_bi_* |, where *M_ai_* is the value of the *i*-th probe on the *a*-th array. Outlier detection was performed by looking for arrays for which the sum of the distances to all other arrays, *S_a_* = Σ*_b_* *d_ab_* was exceptionally large. No such arrays were detected.


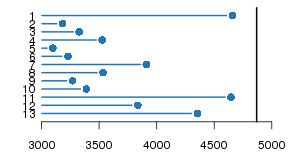


**Figure 2.** Bar chart of the sum of distances to other arrays Sa, the outlier detection criterion from the previous figure. The bars are shown in the original order of the arrays. Based on the distribution of the values across all arrays, a threshold of 4870 was determined, which is indicated by the vertical line. None of the arrays exceeded the threshold and was considered an outlier.

**2 - Outlier detection by [Boxplots](http://www2.informatik.hu-berlin.de/~thomas/qcFinal/#box)**


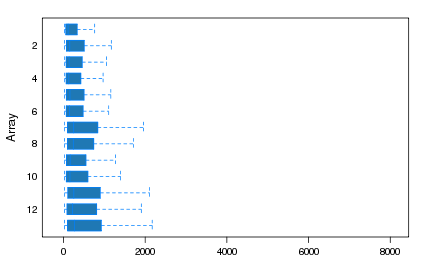


**Figure 3.** Boxplots representing summaries of the signal intensity distributions of the arrays. Each box corresponds to one array. Typically, it is expected that the boxes have similar positions and widths. If the distribution of an array is very different from the others, this may indicate an experimental problem. Outlier detection was performed by computing the Kolmogorov-Smirnov statistic *K_a_* between each array's distribution and the distribution of the pooled data.


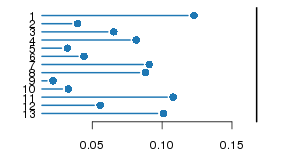


**Figure 4**. Bar chart of the Kolmogorov-Smirnov statistic *K_a_*, the outlier detection criterion from the previous figure. The bars are shown in the original order of the arrays. Based on the distribution of the values across all arrays, a threshold of 0.168 was determined, which is indicated by the vertical line. None of the arrays exceeded the threshold and was considered an outlier.

**3 - Outlier detection by Relative Log Expression (RLE)**


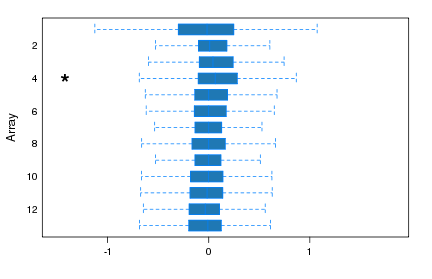


**Figure 5.** *Relative Log Expression (RLE)* plot. Arrays whose boxes are centered away from 0 and/or are more spread out are potentially problematic. Outlier detection was performed by computing the Kolmogorov-Smirnov statistic *R_a_* between each array's RLE values and the pooled, overall distribution of RLE values.


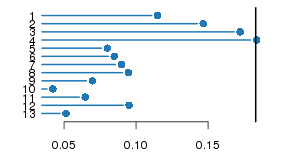


**Figure 6.** Bar chart of the Kolmogorov-Smirnov statistic Ra of the RLE values, the outlier detection criterion from the previous figure. The bars are shown in the original order of the arrays. Based on the distribution of the values across all arrays, a threshold of 0.183 was determined, which is indicated by the vertical line. One array exceeded the threshold.

**4 - Outlier detection by Normalized Unscaled Standard Error (NUSE)**


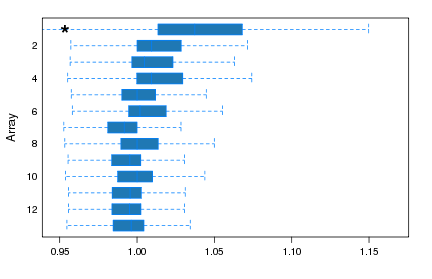


**Figure 7**. *Normalized Unscaled Standard Error (NUSE)* plot. For each array, the boxes should be centered around 1. An array were the values are elevated relative to the other arrays is typically of lower quality. Outlier detection was performed by computing the 75% quantile *N_a_* of each array's NUSE values and looking for arrays with large *N_a_*.


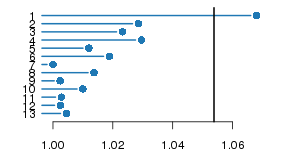

**Figure 8.** Bar chart of the *N_a_*, the outlier detection criterion from the previous figure. The bars are shown in the original order of the arrays. Based on the distribution of the values across all arrays, a threshold of 1.05 was determined, which is indicated by the vertical line. One array exceeded the threshold.

**5 - Outlier detection by MA plots**


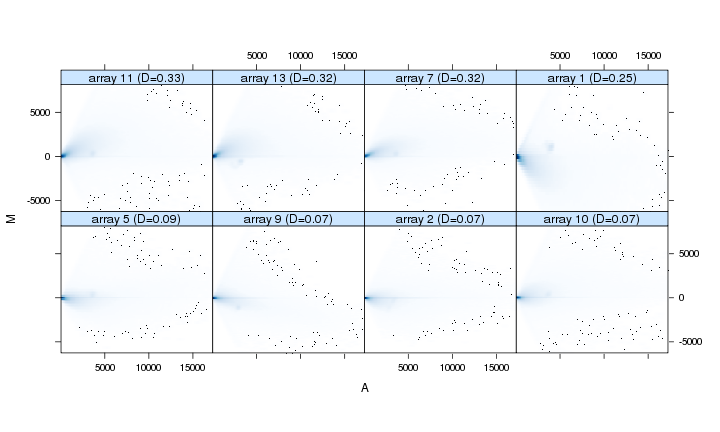


**Figure 9**. MA plots. M and A are defined as: M = log_2_(I_1_) - log_2_(I_2_) A = 1/2 (log_2_(I_1_)+log_2_(I_2_)), where I_1_ is the intensity of the array studied, and I_2_ is the intensity of a "pseudo"-array that consists of the median across arrays. Typically, we expect the mass of the distribution in an MA plot to be concentrated along the M = 0 axis, and there should be no trend in M as a function of A. If there is a trend in the lower range of A, this often indicates that the arrays have different background intensities; this may be addressed by background correction. A trend in the upper range of A can indicate saturation of the measurements; in mild cases, this may be addressed by non-linear normalisation (e.g. quantile normalisation). Outlier detection was performed by computing Hoeffding's statistic *D_a_* on the joint distribution of A and M for each array. Shown are the 4 arrays with the highest value of *D_a_* (top row), and the 4 arrays with the lowest value (bottom row). The value of *D_a_* is shown in the panel headings. 6 arrays had *D_a_*>0.15 and were marked as outliers.


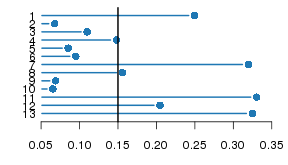

**Figure 10.** Bar chart of the Hoeffding's statistic *D_a_*, the outlier detection criterion from the previous figure. The bars are shown in the original order of the arrays. A threshold of 0.15 was used, which is indicated by the vertical line. 6 arrays exceeded the threshold.

**6 - Outlier detection by Spatial distribution of M.**


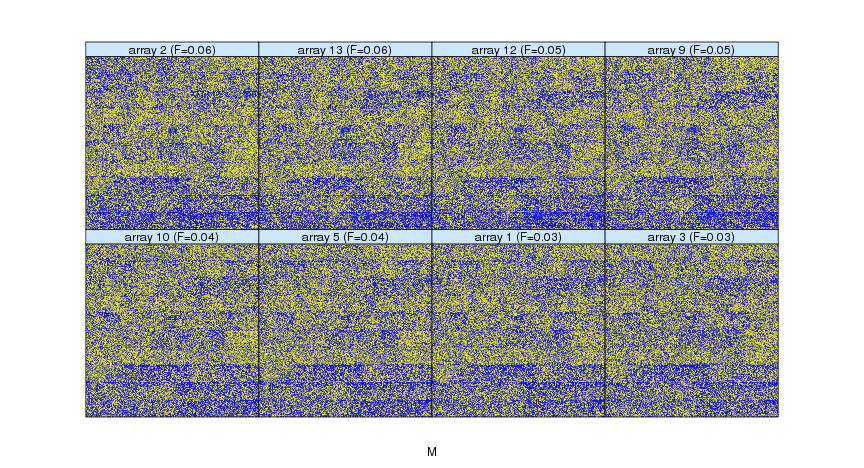

**Figure 11**. False colour representations of the arrays' spatial distributions of feature intensities (M). When the features are distributed randomly on the arrays, a uniform distribution is expected; control features with particularly high or low intensities stand out. The colour scale is proportional to the ranks of the probe intensities. The rank scale has the potential to amplify patterns that are small in amplitude but systematic within an array. It is possible to switch off the rank scaling by modifying the argument scale in the call of the aqm.spatial function. Outlier detection was performed by computing *F_a_* , the sum of the absolutes value of low frequency Fourier coefficients, as a measure of large scale spatial structures. Shown are the 4 arrays with the highest value of *S* (top row), and the 4 arrays with the lowest value (bottom row). The value of *F_a_* is shown in the panel headings.


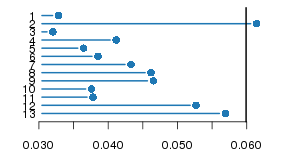


**Figure 12.** Bar chart of the *F_a_*, the outlier detection criterion from the previous figure. The bars are shown in the original order of the arrays. Based on the distribution of the values across all arrays, a threshold of 0.0599 was determined, which is indicated by the vertical line. One array exceeded the threshold.

**Determination of a list of discriminative genes: cross validation procedure**

To find a set of best-discriminating genes, we followed a leave-one-out cross validation strategy. For each cell-line we excluded both samples (0 and 48 hour time points) once and determined the 100 most significant probe sets, allowing the identification of two clock groups. We used moderated t-test to select the top genes by confidence. The set obtained allowed the discrimination at the gene expression level between weak and strong oscillators, when a particular cell line was excluded. Following this procedure we obtained 6 lists. The quality of each list is evaluated as follows: 1) we generated a heatmap using expression profiles for all six cell-lines using the current list of 100 discriminating probes. Results for the different cell lines are shown in Figures 13-19 and Tables 2-8. 2) For heatmaps which correctly cluster the excluded cell-line into the expected group, results are retained. This procedure is repeated for all six different cell-lines. 3) Finally, we generated a discriminative list of 45 genes by taking the intersection between all useful discriminative gene-lists. The resulting list of 45 best-pvalue discriminative genes is presented in Table 9 and illustrated in Figure 20.

**
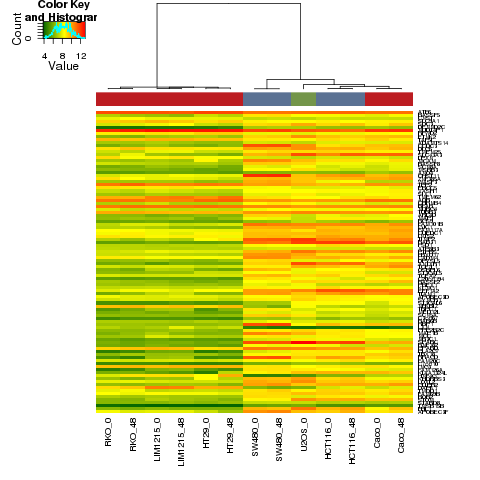
**

**Figure 13.** The arrays for the cell line Caco (Caco_0h and Caco_48h) were excluded. A list of 100 best discriminative genes was retrieved (Table2). The performance of the list was tested by clustering the 13 arrays and the corresponding heatmap is depicted in the figure. Pearson distance function and ward clustering were used. Colour bar on the right corners of the pictures indicates the expression levels for genes in the array, from green (low expressed) to red (high expressed). Colour bar on top of the heatmap indicates class membership. Blue indicates strong oscillator, red indicates weak oscillator, and green indicates reference sample (U2OS).

| **rawp** | **Gene Name** | **Entrez gene ID** | **rawp** | **Gene Name** | **Entrez gene ID** |
| --- | --- | --- | --- | --- | --- |
| 4.26E-12 | **RAB31** | 11031 | 3.02E-06 | **FAM117A** | 81558 |
| 7.48E-12 | **SPARC** | 6678 | 3.33E-06 | **CADM4** | 199731 |
| 2.35E-11 | **PPP2R2C** | 5522 | 3.66E-06 | **CUEDC1** | 404093 |
| 4.61E-11 | **FAM101B** | 359845 | 3.95E-06 | **RFWD2** | 64326 |
| 1.26E-10 | **GLIPR2** | 152007 | 4.53E-06 | **NDRG4** | 65009 |
| 3.38E-10 | **TBX18** | 9096 | 4.86E-06 | **SIX1** | 6495 |
| 7.69E-10 | **DENND2C** | 163259 | 5.21E-06 | **FAM69B** | 138311 |
| 1.45E-09 | **FHL1** | 2273 | 5.55E-06 | **TCF7L1** | 83439 |
| 2.80E-09 | **GJC1** | 10052 | 5.61E-06 | **NFKBIZ** | 64332 |
| 7.19E-09 | **LXN** | 56925 | 5.89E-06 | **PREX1** | 57580 |
| 1.34E-08 | **ETNK2** | 55224 | 6.14E-06 | **ANO4** | 121601 |
| 1.87E-08 | **RNF182** | 221687 | 6.33E-06 | **APOBEC3F** | 200316 |
| 2.67E-08 | **RASSF2** | 9770 | 6.53E-06 | **DAPK1** | 1612 |
| 3.82E-08 | **C20orf194** | 25943 | 7.66E-06 | **WASF3** | 10810 |
| 3.84E-08 | **PTPRS** | 5802 | 7.69E-06 | **NUDT4P1** | 440672 |
| 4.32E-08 | **TIAM1** | 7074 | 7.78E-06 | **KHDRBS3** | 10656 |
| 7.63E-08 | **NR3C1** | 2908 | 7.87E-06 | **RASSF5** | 83593 |
| 8.05E-08 | **LOXL2** | 4017 | 8.37E-06 | **SCN5A** | 6331 |
| 1.02E-07 | **ANTXR1** | 84168 | 8.38E-06 | **ADAMTS14** | 140766 |
| 1.09E-07 | **FSD1** | 79187 | 9.09E-06 | **TMOD1** | 7111 |
| 1.16E-07 | **FAM126A** | 84668 | 1.05E-05 | **OSBPL6** | 114880 |
| 1.50E-07 | **C7orf10** | 79783 | 1.06E-05 | **TMEM62** | 80021 |
| 1.67E-07 | **TMSB15B** | 286527 | 1.25E-05 | **MCC** | 4163 |
| 1.92E-07 | **MED12L** | 116931 | 1.30E-05 | **CHRNB4** | 1143 |
| 1.94E-07 | **C3orf67** | 200844 | 1.44E-05 | **ST3GAL5** | 8869 |
| 2.35E-07 | **RAB6B** | 51560 | 1.51E-05 | **RBP1** | 5947 |
| 2.88E-07 | **FBXO17** | 115290 | 1.61E-05 | **TRPC1** | 7220 |
| 2.89E-07 | **TCEA2** | 6919 | 1.63E-05 | **MAPT** | 4137 |
| 3.22E-07 | **SCN8A** | 6334 | 1.69E-05 | **TAF5L** | 27097 |
| 3.49E-07 | **RRAGD** | 58528 | 1.76E-05 | **TPP2** | 7174 |
| 3.53E-07 | **MAP1B** | 4131 | 1.76E-05 | **BFAR** | 51283 |
| 3.91E-07 | **KCNG1** | 3755 | 1.80E-05 | **SDC3** | 9672 |
| 4.88E-07 | **EEF1A2** | 1917 | 1.83E-05 | **RGS9** | 8787 |
| 5.20E-07 | **PLA2G7** | 7941 | 1.98E-05 | **RASSF8** | 11228 |
| 5.68E-07 | **SYNGR1** | 9145 | 2.01E-05 | **DLG5** | 9231 |
| 6.13E-07 | **ROR2** | 4920 | 2.01E-05 | **TUBB3** | 10381 |
| 7.13E-07 | **SLC43A3** | 29015 | 2.02E-05 | **ST3GAL6** | 10402 |
| 8.04E-07 | **STOM** | 2040 | 2.25E-05 | **ATF6** | 22926 |
| 1.05E-06 | **TMEM25** | 84866 | 2.28E-05 | **APOBEC3D** | 140564 |
| 1.21E-06 | **STARD8** | 9754 | 2.30E-05 | **ARL4D** | 379 |
| 1.58E-06 | **CCDC3** | 83643 | 2.32E-05 | **CNST** | 163882 |
| 1.66E-06 | **DPYSL5** | 56896 | 2.56E-05 | **ZNF385A** | 25946 |
| 1.67E-06 | **KIAA1324L** | 222223 | 2.61E-05 | **AQR** | 9716 |
| 1.69E-06 | **HNF4G** | 3174 | 2.69E-05 | **SLC9A1** | 6548 |
| 1.80E-06 | **FAM20C** | 56975 | 2.73E-05 | **INTS2** | 57508 |
| 1.80E-06 | **TIMP2** | 7077 | 3.01E-05 | **ERCC5** | 2073 |
| 1.89E-06 | **ATP8B3** | 148229 | 3.10E-05 | **PPARD** | 5467 |
| 2.14E-06 | **ING3** | 54556 | 3.13E-05 | **LPXN** | 9404 |
| 2.58E-06 | **VASH1** | 22846 | 3.31E-05 | **MCAM** | 4162 |
| 2.69E-06 | **MSRB3** | 253827 | 3.33E-05 | **CHST11** | 50515 |

**Table 2.** Top 100 discriminative genes excluding the cell line CaCo (18 genes from the final discriminative list-Table 9- where found and are highlighted in green).

**
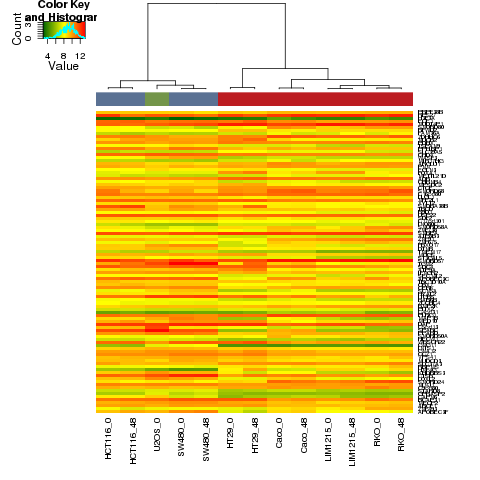
**

**Figure 14.** The arrays for the cell line HT29 (HT29_0h and HT29_48h) were excluded**.** A list of 100 best discriminative genes was retrieved (Table3). The performance of the list was tested by clustering the 13 arrays and the corresponding heatmap is depicted in the figure. Pearson distance function and ward clustering were used. Colour bar on the right corners of the pictures indicates the expression levels for genes in the array, from green (low expressed) to red (high expressed). Colour bar on top of the heatmap indicates class membership. Blue indicates strong oscillator, red indicates weak oscillator, and green indicates reference sample (U2OS).

| **rawp** | **Gene Name** | **Entrez gene ID** | **rawp** | **Gene Name** | **Entrez gene ID** |
| --- | --- | --- | --- | --- | --- |
| 9.22E-08 | **SNORD24** | 26820 | 1.88E-04 | **ZDHHC6** | 64429 |
| 1.38E-06 | **CHD4** | 1108 | 1.88E-04 | **APOBEC3F** | 200316 |
| 4.20E-06 | **ZNF675** | 171392 | 1.91E-04 | **NUDCD3** | 23386 |
| 5.11E-06 | **AQR** | 9716 | 1.96E-04 | **PITX2** | 5308 |
| 6.19E-06 | **ZNF738** | 148203 | 1.98E-04 | **SNORD58A** | 26791 |
| 1.02E-05 | **SNORD80** | 26774 | 2.07E-04 | **ZNF493** | 284443 |
| 1.35E-05 | **IFNGR2** | 3460 | 2.09E-04 | **TBC1D10A** | 83874 |
| 1.97E-05 | **RFWD2** | 64326 | 2.24E-04 | **FOXA1** | 3169 |
| 2.15E-05 | **WASF3** | 10810 | 2.29E-04 | **VPS41** | 27072 |
| 2.46E-05 | **PLRG1** | 5356 | 2.38E-04 | **FBXO17** | 115290 |
| 2.89E-05 | **CCZ1** | 51622 | 2.49E-04 | **ZCCHC4** | 29063 |
| 3.23E-05 | **MOGS** | 7841 | 2.57E-04 | **USP22** | 23326 |
| 3.47E-05 | **PPARD** | 5467 | 2.58E-04 | **BMP2K** | 55589 |
| 4.30E-05 | **NINJ1** | 4814 | 2.64E-04 | **RAB6B** | 51560 |
| 4.71E-05 | **GNG11** | 2791 | 2.72E-04 | **C16orf80** | 29105 |
| 5.16E-05 | **INTS1** | 26173 | 2.74E-04 | **MED10** | 84246 |
| 5.18E-05 | **ATP9A** | 10079 | 2.79E-04 | **SEC14L2** | 23541 |
| 5.89E-05 | **TBCD** | 6904 | 2.79E-04 | **SDC4** | 6385 |
| 6.02E-05 | **MIR17HG** | 407975 | 2.86E-04 | **MAP1B** | 4131 |
| 6.12E-05 | **ABCF1** | 23 | 2.86E-04 | **EGLN3** | 112399 |
| 6.47E-05 | **CTCF** | 10664 | 2.93E-04 | **GNA12** | 2768 |
| 6.49E-05 | **KHDRBS3** | 10656 | 2.93E-04 | **ATP8B3** | 148229 |
| 6.74E-05 | **NUCB2** | 4925 | 2.95E-04 | **H3F3A** | 3020 |
| 6.75E-05 | **ST3GAL5** | 8869 | 3.04E-04 | **DTNB** | 1838 |
| 7.18E-05 | **HYAL2** | 8692 | 3.15E-04 | **TMEM17** | 200728 |
| 7.60E-05 | **ECE1** | 1889 | 3.17E-04 | **B9D1** | 27077 |
| 7.94E-05 | **TSPAN9** | 10867 | 3.18E-04 | **FAM36A** | 116228 |
| 8.08E-05 | **DAP** | 1611 | 3.23E-04 | **RPS27** | 6232 |
| 8.17E-05 | **SNORD57** | 26792 | 3.29E-04 | **BCAP31** | 10134 |
| 8.70E-05 | **C9orf80** | 58493 | 3.37E-04 | **RBPMS** | 11030 |
| 9.48E-05 | **C5orf13** | 9315 | 3.56E-04 | **C4orf31** | 79625 |
| 9.88E-05 | **METTL21D** | 79609 | 3.62E-04 | **INO80C** | 125476 |
| 9.92E-05 | **SPARC** | 6678 | 3.63E-04 | **PDHX** | 8050 |
| 1.02E-04 | **NFE2L1** | 4779 | 3.71E-04 | **LLGL1** | 3996 |
| 1.03E-04 | **XYLT2** | 64132 | 3.78E-04 | **CTSB** | 1508 |
| 1.08E-04 | **CBX7** | 23492 | 3.80E-04 | **ITGB5** | 3693 |
| 1.14E-04 | **ARGLU1** | 55082 | 3.83E-04 | **CHRNB4** | 1143 |
| 1.19E-04 | **GPRASP2** | 114928 | 3.86E-04 | **SDF2** | 6388 |
| 1.37E-04 | **ZNF91** | 7644 | 3.87E-04 | **PPFIBP1** | 8496 |
| 1.40E-04 | **CAPN6** | 827 | 3.94E-04 | **WBSCR22** | 114049 |
| 1.41E-04 | **MESDC2** | 23184 | 4.14E-04 | **SNRPA** | 6626 |
| 1.42E-04 | **SLC37A3** | 84255 | 4.46E-04 | **LOXL2** | 4017 |
| 1.44E-04 | **SLC39A5** | 283375 | 4.58E-04 | **SCN5A** | 6331 |
| 1.51E-04 | **HNF4G** | 3174 | 4.61E-04 | **STARD8** | 9754 |
| 1.65E-04 | **PRPF38B** | 55119 | 4.63E-04 | **HECW1** | 23072 |
| 1.69E-04 | **PNN** | 5411 | 4.74E-04 | **SNORD68** | 606500 |
| 1.72E-04 | **APOBEC3C** | 27350 | 4.94E-04 | **NUDT4P1** | 440672 |
| 1.75E-04 | **SNORA38B** | 100124536 | 5.01E-04 | **C17orf101** | 79701 |
| 1.83E-04 | **MECP2** | 4204 | 5.03E-04 | **TGM2** | 7052 |
| 1.87E-04 | **SNORD50A** | 26799 | 5.04E-04 | **CERK** | 64781 |

**Table 3.** Top 100 discriminative genes excluding the cell line HT29 (45 genes from the final discriminative list-Table 9- where found and are highlighted in green).

**
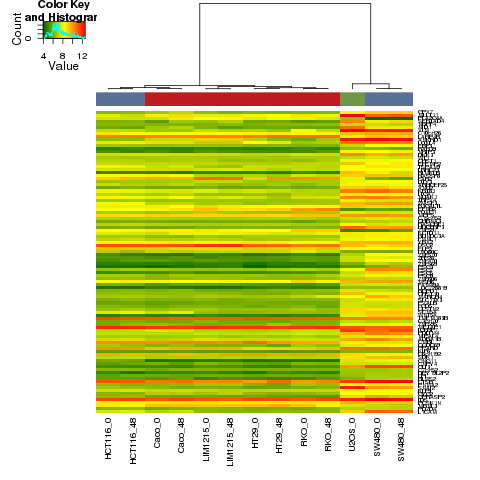
**

**Figure 15.** The arrays for the cell line HCT116 (HCT116_0h and HCT116_48h) were excluded**.** A list of 100 best discriminative genes was retrieved (Table4). The performance of the list was tested by clustering the 13 arrays and the corresponding heatmap is depicted in the figure. Pearson distance function and ward clustering were used. Colour bar on the right corners of the pictures indicates the expression levels for genes in the array, from green (low expressed) to red (high expressed). Colour bar on top of the heatmap indicates class membership. Blue indicates strong oscillator, red indicates weak oscillator, and green indicates reference sample (U2OS).

| **rawp** | **Gene Name** | **Entrez gene ID** | **rawp** | **Gene Name** | **Entrez gene ID** |
| --- | --- | --- | --- | --- | --- |
| **2.68E-12** | **GNG11** | 2791 | 8.93E-07 | **INO80C** | 125476 |
| **5.51E-12** | **NRIP3** | 56675 | 9.18E-07 | **MPP5** | 64398 |
| **7.06E-12** | **ANO2** | 57101 | 9.19E-07 | **LARP4B** | 23185 |
| **2.31E-11** | **GLI3** | 2737 | 9.79E-07 | **KLF9** | 687 |
| **4.38E-11** | **HDGFRP3** | 50810 | 1.19E-06 | **TMEM183B** | 653659 |
| **5.28E-11** | **DKK3** | 27122 | 1.20E-06 | **GYG2** | 8908 |
| **5.45E-11** | **CLSTN2** | 64084 | 1.30E-06 | **PNMA2** | 10687 |
| **9.16E-11** | **FAM49A** | 81553 | 1.41E-06 | **CCDC69** | 26112 |
| **1.13E-10** | **ADRA1B** | 147 | 1.43E-06 | **C10orf26** | 54838 |
| **2.68E-10** | **KRT81** | 3887 | 1.49E-06 | **MLLT11** | 10962 |
| **4.81E-10** | **IGFBP7** | 3490 | 1.51E-06 | **SLC13A3** | 64849 |
| **8.18E-10** | **WASF3** | 10810 | 1.55E-06 | **RGPD1** | 400966 |
| **9.36E-10** | **SDK1** | 221935 | 1.75E-06 | **LPL** | 4023 |
| **1.23E-09** | **ZFP28** | 140612 | 1.77E-06 | **C7orf52** | 375607 |
| **1.49E-09** | **PVALB** | 5816 | 1.81E-06 | **PSG7** | 5676 |
| **1.58E-09** | **VIM** | 7431 | 1.93E-06 | **CHST10** | 9486 |
| **1.65E-09** | **PDLIM4** | 8572 | 2.05E-06 | **SERTAD4** | 56256 |
| **2.51E-09** | **LOC728819** | 728819 | 2.36E-06 | **GPRASP2** | 114928 |
| **2.60E-09** | **WNT5A** | 7474 | 2.56E-06 | **ARHGEF25** | 115557 |
| **8.32E-09** | **FGD5** | 152273 | 2.68E-06 | **PSG9** | 5678 |
| **1.61E-08** | **ZNF470** | 388566 | 2.90E-06 | **CTSB** | 1508 |
| **2.01E-08** | **DPY19L2P2** | 349152 | 2.93E-06 | **ITGA7** | 3679 |
| **4.11E-08** | **ZNF570** | 148268 | 3.08E-06 | **KIF6** | 221458 |
| **5.03E-08** | **CMBL** | 134147 | 3.55E-06 | **PPARD** | 5467 |
| **5.61E-08** | **ZNF597** | 146434 | 3.91E-06 | **SCN5A** | 6331 |
| **5.61E-08** | **SNAI2** | 6591 | 4.11E-06 | **B3GALTL** | 145173 |
| **5.64E-08** | **LOXL4** | 84171 | 4.28E-06 | **KCTD11** | 147040 |
| **6.12E-08** | **CHRNA3** | 1136 | 4.37E-06 | **ZNF606** | 80095 |
| **6.51E-08** | **RIMKLB** | 57494 | 4.74E-06 | **ELP2** | 55250 |
| **6.64E-08** | **CHST1** | 8534 | 5.07E-06 | **FAR2** | 55711 |
| **7.76E-08** | **NBPF4** | 148545 | 5.42E-06 | **WT1** | 7490 |
| **8.85E-08** | **ZNF595** | 152687 | 5.45E-06 | **NID1** | 4811 |
| **1.09E-07** | **YBX2** | 51087 | 5.50E-06 | **ANKRD1** | 27063 |
| **1.10E-07** | **RAB39** | 54734 | 6.03E-06 | **RASGRF1** | 5923 |
| **1.44E-07** | **GPX7** | 2882 | 6.24E-06 | **C6orf192** | 116843 |
| **1.65E-07** | **MBNL3** | 55796 | 6.27E-06 | **EBF3** | 253738 |
| **1.97E-07** | **EFNB2** | 1948 | 6.53E-06 | **IDS** | 3423 |
| **2.05E-07** | **PSG5** | 5673 | 6.83E-06 | **LRRC23** | 10233 |
| **2.12E-07** | **ZNF569** | 148266 | 6.92E-06 | **MSH6** | 2956 |
| **2.24E-07** | **FMNL1** | 752 | 7.23E-06 | **METAP1** | 23173 |
| **2.25E-07** | **C3orf70** | 285382 | 7.27E-06 | **PSG3** | 5671 |
| **2.30E-07** | **RUNDC3A** | 10900 | 7.82E-06 | **DSG2** | 1829 |
| **2.46E-07** | **NIPAL4** | 348938 | 9.56E-06 | **FOXG1** | 2290 |
| **3.12E-07** | **PDZD4** | 57595 | 9.59E-06 | **C15orf52** | 388115 |
| **3.93E-07** | **L1CAM** | 3897 | 9.79E-06 | **TSPAN9** | 10867 |
| **4.13E-07** | **NUAK1** | 9891 | 1.02E-05 | **MARCH4** | 57574 |
| **4.78E-07** | **CNPY4** | 245812 | 1.03E-05 | **RASSF8** | 11228 |
| **5.32E-07** | **EFEMP2** | 30008 | 1.09E-05 | **PLA2G4A** | 5321 |
| **5.56E-07** | **TRPV4** | 59341 | 1.10E-05 | **PCSK1N** | 27344 |
| **8.26E-07** | **DTX3** | 196403 | 1.11E-05 | **RIMS2** | 9699 |

**Table 4.** Top 100 discriminative genes excluding the cell line HCT116 (8 genes from the final discriminative list-Table 9- where found and are highlighted in green).

**
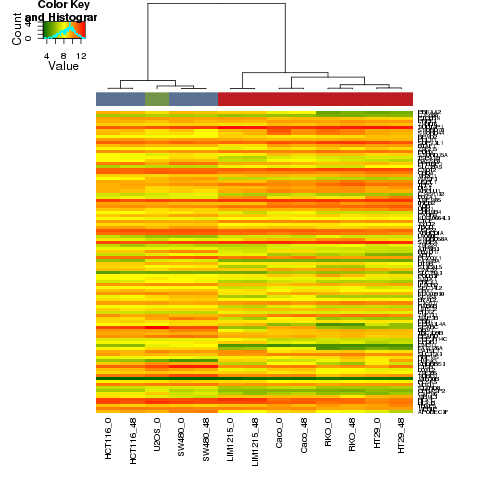
**

**Figure 16.** The arrays for the cell line LIM1215 (LIM1215_0h and LIM1215_48h) were excluded**.** A list of 100 best discriminative genes was retrieved (Table5). The performance of the list was tested by clustering the 13 arrays and the corresponding heatmap is depicted in the figure. Pearson distance function and ward clustering were used. Colour bar on the right corners of the pictures indicates the expression levels for genes in the array, from green (low expressed) to red (high expressed). Colour bar on top of the heatmap indicates class membership. Blue indicates strong oscillator, red indicates weak oscillator, and green indicates reference sample (U2OS).

| **rawp** | **Gene Name** | **Entrez gene ID** | **rawp** | **Gene Name** | **Entrez gene ID** |
| --- | --- | --- | --- | --- | --- |
| **1.44E-06** | **AQR** | 9716 | 3.01E-04 | **ATP8B3** | 148229 |
| **1.89E-05** | **PPARD** | 5467 | 3.05E-04 | **MAP1B** | 4131 |
| **1.94E-05** | **WASF3** | 10810 | 3.17E-04 | **ZNF395** | 55893 |
| **2.05E-05** | **SLC37A3** | 84255 | 3.21E-04 | **SLC39A5** | 283375 |
| **2.42E-05** | **PTGFRN** | 5738 | 3.33E-04 | **CHRNB4** | 1143 |
| **2.44E-05** | **CNOT2** | 4848 | 3.37E-04 | **INO80C** | 125476 |
| **2.51E-05** | **CBX7** | 23492 | 3.43E-04 | **MIR30B** | 407030 |
| **2.58E-05** | **HYAL2** | 8692 | 3.51E-04 | **THAP5** | 168451 |
| **3.17E-05** | **GATSL1** | 389523 | 3.55E-04 | **PAQR4** | 124222 |
| **3.37E-05** | **GNG11** | 2791 | 3.63E-04 | **ST3GAL5** | 8869 |
| **3.63E-05** | **EPB41L4A** | 64097 | 3.77E-04 | **KIAA0664L3** | 100132341 |
| **3.64E-05** | **SLC19A3** | 80704 | 3.79E-04 | **PRR7** | 80758 |
| **3.65E-05** | **CHD4** | 1108 | 3.84E-04 | **ARGLU1** | 55082 |
| **4.27E-05** | **SEC14L2** | 23541 | 3.88E-04 | **NUDT4P1** | 440672 |
| **4.61E-05** | **NINJ1** | 4814 | 3.96E-04 | **SNORD44** | 26806 |
| **5.94E-05** | **PPFIBP1** | 8496 | 4.05E-04 | **MBNL3** | 55796 |
| **6.02E-05** | **TSPAN9** | 10867 | 4.06E-04 | **FAM49A** | 81553 |
| **6.80E-05** | **IREB2** | 3658 | 4.18E-04 | **DTNB** | 1838 |
| **7.06E-05** | **KIAA0930** | 23313 | 4.24E-04 | **CTSB** | 1508 |
| **8.61E-05** | **ZNF224** | 7767 | 4.44E-04 | **SNTA1** | 6640 |
| **1.05E-04** | **DBN1** | 1627 | 4.61E-04 | **STARD8** | 9754 |
| **1.06E-04** | **SPARC** | 6678 | 4.62E-04 | **SCN5A** | 6331 |
| **1.11E-04** | **GPRASP2** | 114928 | 4.83E-04 | **PRPF3** | 9129 |
| **1.15E-04** | **CAPN6** | 827 | 5.00E-04 | **EPDR1** | 54749 |
| **1.21E-04** | **PRKAA2** | 5563 | 5.06E-04 | **LOXL2** | 4017 |
| **1.22E-04** | **USP22** | 23326 | 5.06E-04 | **SNORD15A** | 6079 |
| **1.26E-04** | **XYLT2** | 64132 | 5.12E-04 | **APOBEC3F** | 200316 |
| **1.32E-04** | **PCSK5** | 5125 | 5.16E-04 | **HLA-C** | 3107 |
| **1.37E-04** | **C1orf88** | 128344 | 5.17E-04 | **CWC22** | 57703 |
| **1.44E-04** | **PCYOX1** | 51449 | 5.32E-04 | **C14orf132** | 56967 |
| **1.47E-04** | **TBCD** | 6904 | 5.52E-04 | **MPRIP** | 23164 |
| **1.48E-04** | **PPP1R14C** | 81706 | 5.58E-04 | **TPP2** | 7174 |
| **1.67E-04** | **IFNGR2** | 3460 | 5.63E-04 | **UBR1** | 197131 |
| **1.70E-04** | **HNF4G** | 3174 | 5.67E-04 | **SNORD78** | 692198 |
| **1.87E-04** | **RBPMS** | 11030 | 5.72E-04 | **ARHGDIA** | 396 |
| **1.90E-04** | **ZNF131** | 7690 | 5.72E-04 | **HLA-B** | 3106 |
| **1.98E-04** | **USPL1** | 10208 | 5.80E-04 | **PREX1** | 57580 |
| **2.08E-04** | **SNORD58A** | 26791 | 5.84E-04 | **DIS3** | 22894 |
| **2.31E-04** | **KHDRBS3** | 10656 | 5.90E-04 | **TBC1D9B** | 23061 |
| **2.33E-04** | **RAB6B** | 51560 | 5.94E-04 | **NR2C1** | 7181 |
| **2.35E-04** | **INTS12** | 57117 | 5.95E-04 | **POLR3F** | 10621 |
| **2.36E-04** | **RFWD2** | 64326 | 6.00E-04 | **FOXA1** | 3169 |
| **2.38E-04** | **PITX2** | 5308 | 6.00E-04 | **BTAF1** | 9044 |
| **2.40E-04** | **ZNF215** | 7762 | 6.40E-04 | **NDRG1** | 10397 |
| **2.55E-04** | **ZNHIT6** | 54680 | 6.43E-04 | **FAM126A** | 84668 |
| **2.55E-04** | **CTCF** | 10664 | 6.55E-04 | **C3orf17** | 25871 |
| **2.84E-04** | **SUPV3L1** | 6832 | 6.70E-04 | **TMEM85** | 51234 |
| **2.96E-04** | **SNRPA** | 6626 | 6.71E-04 | **RASSF8** | 11228 |
| **2.96E-04** | **PDHX** | 8050 | 6.78E-04 | **EGLN1** | 54583 |
| **2.97E-04** | **FBXO17** | 115290 | 7.01E-04 | **APLF** | 200558 |

**Table 5.** Top 100 discriminative genes excluding the cell line LIM1215 (45 genes from the final discriminative list-Table 9- where found and are highlighted in green).

**
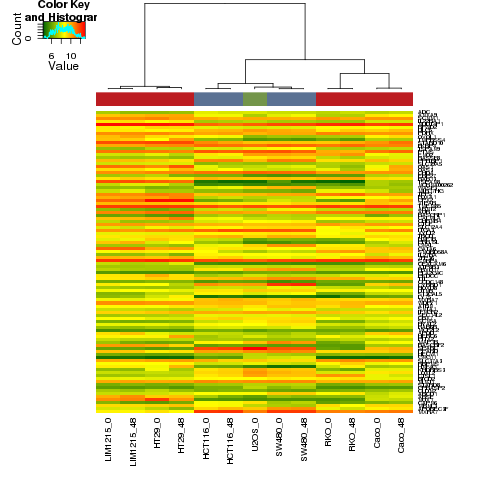
**

**Figure 17.** The arrays for the cell line RKO (RKO_0h and RKO_48h) were excluded**.** A list of 100 best discriminative genes was retrieved (Table6). The performance of the list was tested by clustering the 13 arrays and the corresponding heatmap is depicted in the figure. Pearson distance function and ward clustering were used. Colour bar on the right corners of the pictures indicates the expression levels for genes in the array, from green (low expressed) to red (high expressed). Colour bar on top of the heatmap indicates class membership. Blue indicates strong oscillator, red indicates weak oscillator, and green indicates reference sample (U2OS).

| **rawp** | **Gene Name** | **Entrez gene ID** | **rawp** | **Gene Name** | **Entrez gene ID** |
| --- | --- | --- | --- | --- | --- |
| **3.65E-07** | **NCRNA00262** | 283460 | 2.20E-04 | **ATRN** | 8455 |
| **3.70E-07** | **SLC39A5** | 283375 | 2.21E-04 | **ANXA9** | 8416 |
| **2.98E-06** | **CTSL1** | 1514 | 2.23E-04 | **VIPAR** | 63894 |
| **7.00E-06** | **GPX2** | 2877 | 2.25E-04 | **SCNM1** | 79005 |
| **8.44E-06** | **DTNB** | 1838 | 2.31E-04 | **CLDN2** | 9075 |
| **8.66E-06** | **ITGA3** | 3675 | 2.52E-04 | **STARD10** | 10809 |
| **1.28E-05** | **CHD4** | 1108 | 2.62E-04 | **RAB6B** | 51560 |
| **1.75E-05** | **SEC14L2** | 23541 | 2.65E-04 | **IL27RA** | 9466 |
| **2.01E-05** | **WASF3** | 10810 | 2.72E-04 | **ATP8B3** | 148229 |
| **2.03E-05** | **FOXA1** | 3169 | 2.73E-04 | **SNRPA** | 6626 |
| **2.95E-05** | **XYLT2** | 64132 | 2.76E-04 | **PRR15L** | 79170 |
| **3.36E-05** | **AQR** | 9716 | 2.83E-04 | **FBXO17** | 115290 |
| **3.82E-05** | **ENO2** | 2026 | 2.99E-04 | **MAP1B** | 4131 |
| **4.52E-05** | **GNG11** | 2791 | 3.01E-04 | **TTL** | 150465 |
| **4.61E-05** | **HYAL2** | 8692 | 3.07E-04 | **SNORD58A** | 26791 |
| **5.35E-05** | **NINJ1** | 4814 | 3.14E-04 | **HERC6** | 55008 |
| **5.36E-05** | **HOXD8** | 3234 | 3.21E-04 | **WDFY1** | 57590 |
| **5.51E-05** | **BICD2** | 23299 | 3.28E-04 | **NUDT4P1** | 440672 |
| **6.77E-05** | **NOX1** | 27035 | 3.39E-04 | **MCF2L2** | 23101 |
| **7.25E-05** | **ABCD1** | 215 | 3.47E-04 | **RAB13** | 5872 |
| **7.80E-05** | **PPARD** | 5467 | 3.54E-04 | **PPFIBP1** | 8496 |
| **7.84E-05** | **IFNGR2** | 3460 | 3.66E-04 | **TMEM85** | 51234 |
| **9.11E-05** | **CTCF** | 10664 | 3.67E-04 | **CHRNB4** | 1143 |
| **9.14E-05** | **TSPAN8** | 7103 | 3.71E-04 | **PDHX** | 8050 |
| **9.34E-05** | **TBCD** | 6904 | 3.81E-04 | **RASGRF2** | 5924 |
| **9.76E-05** | **HNF1B** | 6928 | 3.82E-04 | **LY75** | 4065 |
| **9.77E-05** | **SPARC** | 6678 | 4.00E-04 | **CYBRD1** | 79901 |
| **1.04E-04** | **HECW1** | 23072 | 4.02E-04 | **MXRA7** | 439921 |
| **1.06E-04** | **RASGRP1** | 10125 | 4.03E-04 | **MIR17HG** | 407975 |
| **1.07E-04** | **KHDRBS3** | 10656 | 4.04E-04 | **STARD8** | 9754 |
| **1.10E-04** | **RFWD2** | 64326 | 4.19E-04 | **OVOL1** | 5017 |
| **1.17E-04** | **ARSE** | 415 | 4.21E-04 | **ARNT2** | 9915 |
| **1.22E-04** | **ERP27** | 121506 | 4.25E-04 | **MXRA7** | 439921 |
| **1.28E-04** | **OAS1** | 4938 | 4.29E-04 | **SNTA1** | 6640 |
| **1.35E-04** | **CAPN6** | 827 | 4.31E-04 | **PLA2G4C** | 8605 |
| **1.38E-04** | **HNF4G** | 3174 | 4.33E-04 | **RASSF8** | 11228 |
| **1.41E-04** | **TSPAN9** | 10867 | 4.37E-04 | **TPP2** | 7174 |
| **1.43E-04** | **PITX2** | 5308 | 4.37E-04 | **FKBP4** | 2288 |
| **1.44E-04** | **MBNL3** | 55796 | 4.44E-04 | **TMPRSS4** | 56649 |
| **1.48E-04** | **GNAL** | 2774 | 4.48E-04 | **DLG5** | 9231 |
| **1.50E-04** | **IL22RA1** | 58985 | 4.49E-04 | **SCN5A** | 6331 |
| **1.80E-04** | **SLC37A3** | 84255 | 4.60E-04 | **PKDCC** | 91461 |
| **1.86E-04** | **GPRASP2** | 114928 | 4.66E-04 | **APOBEC3F** | 200316 |
| **1.86E-04** | **CBX7** | 23492 | 4.72E-04 | **OAS3** | 4940 |
| **1.87E-04** | **ST3GAL5** | 8869 | 4.73E-04 | **PTMS** | 5763 |
| **1.92E-04** | **ZFP36** | 7538 | 4.74E-04 | **CCDC148** | 130940 |
| **1.93E-04** | **CALML4** | 91860 | 4.75E-04 | **APOD** | 347 |
| **1.97E-04** | **RBPMS** | 11030 | 4.76E-04 | **GATA6** | 2627 |
| **2.06E-04** | **CEACAM6** | 4680 | 4.78E-04 | **LOXL2** | 4017 |
| **2.12E-04** | **SLC12A4** | 6560 | 4.81E-04 | **ADC** | 113451 |

**Table 6.** Top 100 discriminative genes excluding the cell line RKO (41 genes from the final discriminative list-Table 9- where found and are highlighted in green).

**
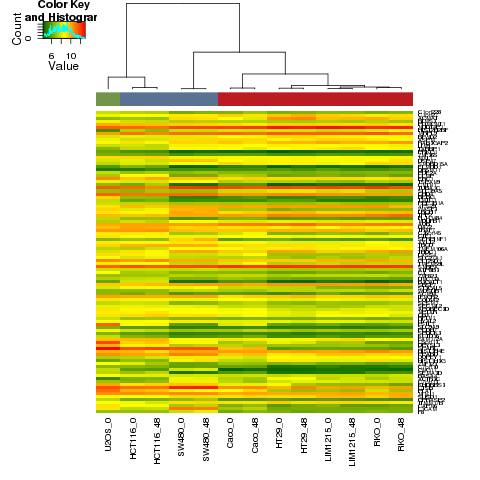
**

**Figure 18.** The arrays for the cell line SW480 (SW480_0h and SW480_48h) were excluded**.** A list of 100 best discriminative genes was retrieved (Table7). The performance of the list was tested by clustering the 13 arrays and the corresponding heatmap is depicted in the figure.

Pearson distance function and ward clustering were used. Colour bar on the right corners of the pictures indicates the expression levels for genes in the array, from green (low expressed) to red (high expressed). Colour bar on top of the heatmap indicates class membership. Blue indicates strong oscillator, red indicates weak oscillator, and green indicates reference sample (U2OS).

| **rawp** | **Gene Name** | **Entrez gene ID** | **rawp** | **Gene Name** | **Entrez gene ID** |
| --- | --- | --- | --- | --- | --- |
| **1.28E-07** | **CAPN6** | 827 | 2.83E-04 | **HYAL2** | 8692 |
| **8.55E-07** | **PRKG1** | 5592 | 3.01E-04 | **CTF1** | 1489 |
| **3.50E-06** | **DPYSL3** | 1809 | 3.04E-04 | **PLAT** | 5327 |
| **3.77E-06** | **SEMA3D** | 223117 | 3.16E-04 | **VPS29** | 51699 |
| **1.08E-05** | **WASF3** | 10810 | 3.28E-04 | **CBX7** | 23492 |
| **1.34E-05** | **ELMOD1** | 55531 | 3.34E-04 | **OR5M11** | 219487 |
| **3.65E-05** | **LIN9** | 286826 | 3.46E-04 | **WDSUB1** | 151525 |
| **3.69E-05** | **UNC13A** | 23025 | 3.63E-04 | **LASS4** | 79603 |
| **3.80E-05** | **GNG11** | 2791 | 3.81E-04 | **CLMP** | 79827 |
| **4.01E-05** | **TARBP1** | 6894 | 3.87E-04 | **CTSB** | 1508 |
| **4.16E-05** | **PPP1R1A** | 5502 | 3.89E-04 | **RIMS3** | 9783 |
| **4.26E-05** | **C1orf228** | 339541 | 4.13E-04 | **C3** | 718 |
| **4.42E-05** | **NUDT4P1** | 440672 | 4.16E-04 | **RAB3GAP2** | 25782 |
| **4.51E-05** | **SNRPA** | 6626 | 4.19E-04 | **SGSM1** | 129049 |
| **4.74E-05** | **TIMM17B** | 10245 | 4.25E-04 | **F8** | 2157 |
| **5.00E-05** | **AQR** | 9716 | 4.26E-04 | **RFTN1** | 23180 |
| **5.13E-05** | **B3GALT1** | 8708 | 4.27E-04 | **TSPAN9** | 10867 |
| **6.14E-05** | **SLC9A9** | 285195 | 4.44E-04 | **SCN9A** | 6335 |
| **6.36E-05** | **SLC27A1** | 376497 | 4.48E-04 | **C7orf10** | 79783 |
| **7.15E-05** | **CNTN1** | 1272 | 4.50E-04 | **MRPL9** | 65005 |
| **8.06E-05** | **SRSF7** | 6432 | 4.57E-04 | **TUBA1C** | 84790 |
| **8.36E-05** | **MXI1** | 4601 | 4.58E-04 | **RFWD2** | 64326 |
| **9.44E-05** | **AGMAT** | 79814 | 4.69E-04 | **RERG** | 85004 |
| **9.92E-05** | **OAZ2** | 4947 | 4.75E-04 | **TMEM59L** | 25789 |
| **9.96E-05** | **TMEM106A** | 113277 | 4.80E-04 | **XYLT2** | 64132 |
| **1.10E-04** | **CHD4** | 1108 | 4.81E-04 | **PCDH7** | 5099 |
| **1.15E-04** | **ASPM** | 259266 | 5.01E-04 | **L1CAM** | 3897 |
| **1.16E-04** | **C5orf13** | 9315 | 5.16E-04 | **IFNGR2** | 3460 |
| **1.40E-04** | **SPOCK3** | 50859 | 5.24E-04 | **HIST1H4E** | 8367 |
| **1.47E-04** | **SPARC** | 6678 | 5.30E-04 | **SEC14L2** | 23541 |
| **1.50E-04** | **ERC1** | 23085 | 5.34E-04 | **HIST1H3G** | 8355 |
| **1.74E-04** | **SUSD1** | 64420 | 5.37E-04 | **TRAF7** | 84231 |
| **1.81E-04** | **PDE2A** | 5138 | 5.46E-04 | **C1orf27** | 54953 |
| **1.84E-04** | **TPP2** | 7174 | 5.51E-04 | **ATP8B3** | 148229 |
| **1.97E-04** | **SERPINF1** | 5176 | 5.52E-04 | **C7orf13** | 129790 |
| **1.98E-04** | **ZNF365** | 22891 | 5.59E-04 | **FSTL1** | 11167 |
| **2.11E-04** | **NDUFB1** | 4707 | 5.63E-04 | **GPRASP2** | 114928 |
| **2.11E-04** | **NPTXR** | 23467 | 5.65E-04 | **KHDRBS3** | 10656 |
| **2.18E-04** | **GLT25D1** | 79709 | 5.72E-04 | **DOPEY1** | 23033 |
| **2.28E-04** | **SNORD15A** | 6079 | 5.79E-04 | **TBCD** | 6904 |
| **2.44E-04** | **ZNF323** | 64288 | 5.82E-04 | **SLC39A5** | 283375 |
| **2.45E-04** | **C16orf45** | 89927 | 5.93E-04 | **LRCH1** | 23143 |
| **2.45E-04** | **NINJ1** | 4814 | 5.95E-04 | **APOBEC3D** | 140564 |
| **2.50E-04** | **COL6A2** | 1292 | 6.09E-04 | **HIST2H2BF** | 440689 |
| **2.55E-04** | **USP47** | 55031 | 6.10E-04 | **ACTR3C** | 653857 |
| **2.63E-04** | **RASA4** | 10156 | 6.13E-04 | **TMTC1** | 83857 |
| **2.63E-04** | **FAM172A** | 83989 | 6.16E-04 | **KCTD16** | 57528 |
| **2.67E-04** | **POMGNT1** | 55624 | 6.20E-04 | **THOC1** | 9984 |
| **2.73E-04** | **PPARD** | 5467 | 6.25E-04 | **ST3GAL5** | 8869 |
| **2.81E-04** | **FLJ41484** | 650669 | 6.28E-04 | **ZNF823** | 55552 |

**Table 7.** Top 100 discriminative genes excluding the cell line SW480 (24 genes from the final discriminative list-Table 9- where found and are highlighted in green).

**
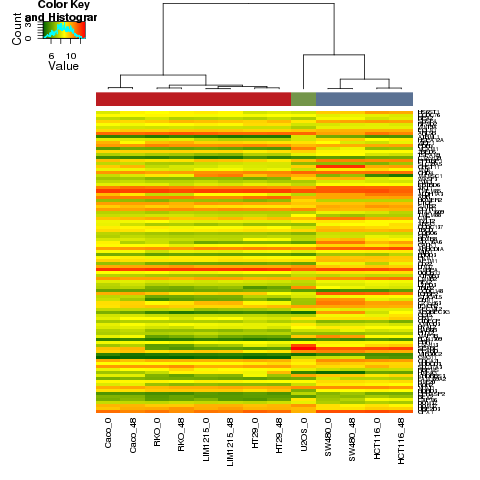
**

**Figure 19.** The arrays for the cell line U2OS (U2OS_0h and U2OS_48h) were excluded**.** A list of 100 best discriminative genes was retrieved (Table8). The performance of the list was tested by clustering the 13 arrays and the corresponding heatmap is depicted in the figure. Pearson distance function and ward clustering were used. Colour bar on the right corners of the pictures indicates the expression levels for genes in the array, from green (low expressed) to red (high expressed). Colour bar on top of the heatmap indicates class membership. Blue indicates strong oscillator, red indicates weak oscillator, and green indicates reference sample (U2OS).

| **rawp** | **Gene Name** | **Entrez gene ID** | **rawp** | **Gene Name** | **Entrez gene ID** |
| --- | --- | --- | --- | --- | --- |
| **6.77E-07** | **FAM189A2** | 9413 | 1.35E-04 | **RAB6B** | 51560 |
| **1.74E-06** | **TBCD** | 6904 | 1.41E-04 | **ATRNL1** | 26033 |
| **2.56E-06** | **PPFIBP1** | 8496 | 1.43E-04 | **TSPAN9** | 10867 |
| **3.06E-06** | **FHOD3** | 80206 | 1.47E-04 | **SLC37A3** | 84255 |
| **4.39E-06** | **APOBEC3G** | 60489 | 1.55E-04 | **NUDCD3** | 23386 |
| **4.58E-06** | **CD22** | 933 | 1.56E-04 | **EGLN1** | 54583 |
| **5.62E-06** | **LIN37** | 55957 | 1.57E-04 | **ALDH1A3** | 220 |
| **7.04E-06** | **HOMER2** | 9455 | 1.59E-04 | **AP1M1** | 8907 |
| **1.01E-05** | **ARHGDIA** | 396 | 1.67E-04 | **TRIB2** | 28951 |
| **1.06E-05** | **GLYR1** | 84656 | 1.69E-04 | **CCDC76** | 54482 |
| **1.09E-05** | **SLC16A6** | 9120 | 1.72E-04 | **GPX1** | 2876 |
| **1.23E-05** | **PKN1** | 5585 | 1.72E-04 | **HOXB8** | 3218 |
| **1.44E-05** | **MVK** | 4598 | 1.75E-04 | **NCAM1** | 4684 |
| **1.50E-05** | **UQCR11** | 10975 | 1.78E-04 | **IFNGR2** | 3460 |
| **1.83E-05** | **IGFBP2** | 3485 | 1.81E-04 | **HNF4G** | 3174 |
| **1.95E-05** | **DOM3Z** | 1797 | 1.85E-04 | **WEE1** | 7465 |
| **2.25E-05** | **HDHD3** | 81932 | 1.93E-04 | **MIR30C2** | 407032 |
| **2.28E-05** | **WNT9A** | 7483 | 1.97E-04 | **SNTB2** | 6645 |
| **2.56E-05** | **SP6** | 80320 | 2.01E-04 | **BEX2** | 84707 |
| **2.58E-05** | **WASF3** | 10810 | 2.01E-04 | **ARL5B** | 221079 |
| **2.99E-05** | **LYPD3** | 27076 | 2.03E-04 | **CDCA7L** | 55536 |
| **3.03E-05** | **HYAL2** | 8692 | 2.06E-04 | **MIB1** | 57534 |
| **3.46E-05** | **GALK1** | 2584 | 2.07E-04 | **CORO6** | 84940 |
| **3.52E-05** | **AQR** | 9716 | 2.10E-04 | **PITX2** | 5308 |
| **3.62E-05** | **GPRASP2** | 114928 | 2.12E-04 | **UBE2D3** | 7323 |
| **3.71E-05** | **ZBED5** | 58486 | 2.15E-04 | **RPA2** | 6118 |
| **3.89E-05** | **NINJ1** | 4814 | 2.15E-04 | **SEC14L2** | 23541 |
| **3.98E-05** | **CHD4** | 1108 | 2.25E-04 | **CCDC137** | 339230 |
| **4.09E-05** | **HSPA12A** | 259217 | 2.27E-04 | **CHST11** | 50515 |
| **5.53E-05** | **RBPMS** | 11030 | 2.27E-04 | **CNP** | 1267 |
| **6.00E-05** | **GNG11** | 2791 | 2.37E-04 | **CTCF** | 10664 |
| **6.06E-05** | **LMNB2** | 84823 | 2.40E-04 | **KIAA1609** | 57707 |
| **6.96E-05** | **DOM3Z** | 1797 | 2.44E-04 | **FLJ41309** | 645079 |
| **7.19E-05** | **CAPN6** | 827 | 2.54E-04 | **HS6ST1** | 9394 |
| **7.90E-05** | **TMEM88** | 92162 | 2.57E-04 | **AMIGO3** | 386724 |
| **8.02E-05** | **GGT5** | 2687 | 2.57E-04 | **PDHX** | 8050 |
| **8.78E-05** | **RNF20** | 56254 | 2.59E-04 | **AKT1** | 207 |
| **9.08E-05** | **PPARD** | 5467 | 2.66E-04 | **ATP8B3** | 148229 |
| **9.68E-05** | **RFX2** | 5990 | 2.67E-04 | **RFWD2** | 64326 |
| **9.75E-05** | **ARSG** | 22901 | 2.69E-04 | **CHN1** | 1123 |
| **1.01E-04** | **KBTBD6** | 89890 | 2.76E-04 | **MANSC1** | 54682 |
| **1.06E-04** | **CIDECP** | 152302 | 2.77E-04 | **SNRPA** | 6626 |
| **1.09E-04** | **XYLT2** | 64132 | 2.78E-04 | **C5orf13** | 9315 |
| **1.10E-04** | **ROR2** | 4920 | 2.82E-04 | **CCDC148** | 130940 |
| **1.11E-04** | **PRR7** | 80758 | 2.83E-04 | **SLC39A5** | 283375 |
| **1.12E-04** | **CBX7** | 23492 | 2.95E-04 | **MAP1B** | 4131 |
| **1.19E-04** | **GJA3** | 2700 | 2.96E-04 | **TMEM85** | 51234 |
| **1.23E-04** | **ST3GAL5** | 8869 | 2.98E-04 | **C21orf63** | 59271 |
| **1.24E-04** | **SRSF4** | 6429 | 2.99E-04 | **KHDRBS3** | 10656 |
| **1.32E-04** | **SPARC** | 6678 | 3.00E-04 | **C12orf39** | 80763 |

**Table 8.** Top 100 discriminative genes excluding the cell line U2OS (31 genes from the final discriminative list-Table 9- where found and are highlighted in green).

| **Gene name** | **Gene ID** | **p-value** | **Description (DAVID)** | **KEGG_PATHWAY** |
| --- | --- | --- | --- | --- |
| **APOBEC3F** | 200316 | 1.44E-04 | apolipoprotein B mRNA editing enzyme, catalytic polypeptide-like 3F |  |
| **AQR** | 9716 | 1.13E-05 | aquarius homolog (mouse) | Spliceosome |
| **ARGLU1** | 55082 | 3.55E-04 | arginine and glutamate rich 1 |  |
| **ATP8B3** | 148229 | 1.32E-04 | ATPase, class I, type 8B, member 3 |  |
| **CAPN6** | 827 | 2.94E-05 | calpain 6 |  |
| **CBX7** | 23492 | 5.13E-05 | chromobox homolog 7 |  |
| **CHD4** | 1108 | 1.56E-05 | chromodomain helicase DNA binding protein 4 |  |
| **CHRNB4** | 1143 | 9.40E-05 | cholinergic receptor, nicotinic, beta 4 |  |
| **CTCF** | 10664 | 1.06E-04 | CCCTC-binding factor (zinc finger protein) |  |
| **CTSB** | 1508 | 1.19E-04 | cathepsin B | Lysosome, Antigen processing and presentation |
| **DTNB** | 1838 | 1.19E-04 | dystrobrevin, beta |  |
| **FBXO17** | 115290 | 6.52E-05 | F-box protein 17 |  |
| **FOXA1** | 3169 | 1.70E-04 | forkhead box A1 |  |
| **GNG11** | 2791 | 9.70E-06 | guanine nucleotide binding protein (G protein), gamma 11 | Chemokine signaling pathway |
| **GPRASP2** | 114928 | 4.88E-05 | G protein-coupled receptor associated sorting protein 2 |  |
| **HNF4G** | 3174 | 3.76E-05 | hepatocyte nuclear factor 4, gamma | Maturity onset diabetes of the young |
| **HYAL2** | 8692 | 2.82E-05 | hyaluronoglucosaminidase 2 | Glycosaminoglycan degradation |
| **IFNGR2** | 3460 | 5.83E-05 | interferon gamma receptor 2 (interferon gamma transducer 1) | Cytokine-cytokine receptor interaction, Jak-STAT signaling pathway, Natural killer cell mediated cytotoxicity |
| **INO80C** | 125476 | 1.36E-04 | INO80 complex subunit C |  |
| **KHDRBS3** | 10656 | 5.49E-05 | KH domain containing, RNA binding, signal transduction associated 3 |  |
| **LOXL2** | 4017 | 1.26E-04 | lysyl oxidase-like 2 |  |
| **MAP1B** | 4131 | 6.93E-05 | microtubule-associated protein 1B |  |
| **NINJ1** | 4814 | 3.18E-05 | ninjurin 1 |  |
| **NUDT4P1** | 440672 | 1.31E-04 | nudix (nucleoside diphosphate linked moiety X)-type motif 4; nudix (nucleoside diphosphate linked moiety X)-type motif 4 pseudogene 1 |  |
| **PDHX** | 8050 | 1.52E-04 | pyruvate dehydrogenase complex, component X |  |
| **PITX2** | 5308 | 9.84E-05 | paired-like homeodomain 2 | TGF-beta signaling pathway |
| **PPARδ** | 5467 | 1.96E-05 | peroxisome proliferator-activated receptor delta | PPAR signaling pathway, Wnt signaling pathway, Pathways in cancer, Acute myeloid leukemia |
| **PPFIBP1** | 8496 | 9.75E-05 | PTPRF interacting protein, binding protein 1 (liprin beta 1) |  |
| **RAB6B** | 51560 | 6.76E-05 | RAB6B, member RAS oncogene family |  |
| **RBPMS** | 11030 | 1.16E-04 | RNA binding protein with multiple splicing |  |
| **RFWD2** | 64326 | 6.53E-05 | ring finger and WD repeat domain 2 | p53 signaling pathway,hsa04120:Ubiquitin mediated proteolysis |
| **SCN5α** | 6331 | 1.06E-04 | sodium channel, voltage-gated, type V, alpha subunit |  |
| **Gene name** | **ID** | **p-value** | **Description (DAVID)** | **KEGG_PATHWAY** |
| **SEC14L2** | 23541 | 7.94E-05 | SEC14-like 2 (S. cerevisiae) |  |
| **SLC37A3** | 84255 | 5.70E-05 | solute carrier family 37 (glycerol-3-phosphate transporter), member 3 |  |
| **SLC39A5** | 283375 | 8.42E-05 | solute carrier family 39 (metal ion transporter), member 5 |  |
|  |  |  |  |  |
| **SNORD58A** | 26791 | 3.04E-04 | U58 small nucleolar RNA |  |
| **SNRPA** | 6626 | 1.70E-04 | small nuclear ribonucleoprotein polypeptide A | Spliceosome |
| **SPARC** | 6678 | 2.15E-05 | secreted protein, acidic, cysteine-rich (osteonectin) |  |
| **ST3GAL5** | 8869 | 1.02E-04 | ST3 beta-galactoside alpha-2,3-sialyltransferase 5 | Glycosphingolipid biosynthesis |
| **STARD8** | 9754 | 1.33E-04 | StAR-related lipid transfer (START) domain containing 8 |  |
| **TBCD** | 6904 | 4.37E-05 | tubulin folding cofactor D |  |
| **TSPAN9** | 10867 | 4.31E-05 | tetraspanin 9 |  |
| **USP22** | 23326 | 3.03E-04 | ubiquitin specific peptidase 22 |  |
| **WASF3** | 10810 | 3.81E-06 | WAS protein family, member 3 | Adherens junction, Fc gamma R-mediated phagocytosis |
| **XYLT2** | 64132 | 4.02E-05 | xylosyltransferase II | Chondroitin sulfate biosynthesis, Heparan sulfate biosynthesis |

**Table 9.** **List of discriminative genes.** The list of genes depicted in this table was obtained from the intersection of the results in Tables 3 and 5. Raw p-values are derived using moderated t-test between groups of strong versus weak oscillators.

**Figure 20.** Heatmap generated with the list of 45 discriminative genes. Pearson distance function and ward clustering were used. Colour bar on the right corners of the pictures indicates the expression levels for genes in the array, from green (low expressed) to red (high expressed). Colour bar on top of the heatmap indicates class membership. Blue indicates strong oscillator, red indicates weak oscillator, and green indicates reference sample (U2OS). Genes are ordered by profile similarity.

**References**

1. Kauffmann A, Gentleman R, Huber W (2009) arrayQualityMetrics--a bioconductor package for quality assessment of microarray data. Bioinformatics 25: 415-416.
