# Supplementary material for: Ras-Mediated Deregulation of the Circadian Clock in Cancer
Source: PLoS Genet. 2014 May 29;10(5):e1004338. doi: 10.1371/journal.pgen.1004338 (PMC4038477; doi:10.1371/journal.pgen.1004338)
Supplement: Text S2 — A comprehensive regulatory network for the mammalian circadian clock. (DOC) [file pgen.1004338.s011.doc]

**Text S2 – A comprehensive regulatory network for the mammalian circadian clock.**

We assembled a comprehensive genetic network for the mammalian circadian clock, based on a network of clock and clock-controlled genes interactions which we previously developed. We used a text mining approach to complete the initial network and curated all interactions found; as a result we obtained a circadian clock network system presented in the main text, Figure 2B. All genes present in the network are indicated in Table 1, together with the corresponding Gene Entrez ID (ID) as well as a description and molecular pathways were the gene has been reported to be involved retrieved from KEGG. In Table2 we list all interactions present in the network and the corresponding references.

| **ID** | **Symbol** | **Description** | **KEGG_PATHWAY** |
| --- | --- | --- | --- |
| **196** | **AHR** | aryl hydrocarbon receptor |  |
| **211** | **ALAS1** | aminolevulinate, delta-, synthase 1 | Glycine, serine and threonine metabolism, Porphyrin and chlorophyll metabolism, |
| **5562** | **AMPK** | protein kinase, AMP-activated, alpha 1 catalytic subunit | hsa04140:Regulation of autophagy,hsa04150:mTOR signaling pathway,hsa04910:Insulin signaling pathway,hsa04920:Adipocytokine signaling pathway,hsa05410:Hypertrophic cardiomyopathy (HCM), |
| **8945** | **βTRCP** | beta-transducin repeat containing | hsa04114:Oocyte meiosis,hsa04120:Ubiquitin mediated proteolysis,hsa04310:Wnt signaling pathway,hsa04340:Hedgehog signaling pathway, |
| **406** | **BMAL1** | aryl hydrocarbon receptor nuclear translocator-like | hsa04710:Circadian rhythm, |
| **56938** | **BMAL2** | aryl hydrocarbon receptor nuclear translocator-like 2 |  |
| **9970** | **CAR** | nuclear receptor subfamily 1, group I, member 3 |  |
| **1387** | **CBP** | CREB binding protein | hsa04110:Cell cycle,hsa04310:Wnt signaling pathway,hsa04330:Notch signaling pathway,hsa04350:TGF-beta signaling pathway,hsa04520:Adherens junction,hsa04630:Jak-STAT signaling pathway,hsa04720:Long-term potentiation,hsa04916:Melanogenesis,hsa05016:Huntington's disease,hsa05200:Pathways in cancer,hsa05211:Renal cell carcinoma,hsa05215:Prostate cancer, |
| **9575** | **CLOCK** | clock homolog (mouse) | hsa04710:Circadian rhythm, |
| **1385** | **CREB** | cAMP responsive element binding protein 1 | hsa04612:Antigen processing and presentation,hsa04916:Melanogenesis,hsa05016:Huntington's disease,hsa05215:Prostate cancer, |
| **1407** | **CRY1** | cryptochrome 1 (photolyase-like) | hsa04710:Circadian rhythm, |
| **1408** | **CRY2** | cryptochrome 2 (photolyase-like) | hsa04710:Circadian rhythm, |
| **1453** | **CSNK1** | casein kinase 1, delta | hsa04340:Hedgehog signaling pathway,hsa04540:Gap junction,hsa04710:Circadian rhythm, |
| **1454** | **CSNK1ε** | casein kinase 1, epsilon | hsa04310:Wnt signaling pathway,hsa04340:Hedgehog signaling pathway,hsa04710:Circadian rhythm, |
| **1457** | **CSNK2α** | casein kinase 2, alpha 1 polypeptide pseudogene; casein kinase 2, alpha 1 polypeptide | hsa04310:Wnt signaling pathway,hsa04520:Adherens junction,hsa04530:Tight junction, |
| **1628** | **DBP** | D site of albumin promoter (albumin D-box) binding protein |  |
| **8553** | **DEC1** | basic helix-loop-helix family, member e40 | hsa04710:Circadian rhythm, |
| **79365** | **DEC2** | basic helix-loop-helix family, member e41 | hsa04710:Circadian rhythm, |
| **4783** | **E4BP4** | nuclear factor, interleukin 3 regulated |  |
| **26224** | **FBXL3** | F-box and leucine-rich repeat protein 3 |  |
| **2932** | **GSK3β** | glycogen synthase kinase 3 beta | hsa04012:ErbB signaling pathway,hsa04062:Chemokine signaling pathway,hsa04110:Cell cycle,hsa04310:Wnt signaling pathway,hsa04340:Hedgehog signaling pathway,hsa04360:Axon guidance,hsa04510:Focal adhesion,hsa04660:T cell receptor signaling pathway,hsa04662:B cell receptor signaling pathway,hsa04722:Neurotrophin signaling pathway,hsa04910:Insulin signaling pathway,hsa04916:Melanogenesis,hsa05010:Alzheimer's disease,hsa05200:Pathways in cancer,hsa05210:Colorectal cancer,hsa05213:Endometrial cancer,hsa05215:Prostate cancer,hsa05217:Basal cell carcinoma, |
| **3131** | **HLF** | hepatic leukemia factor |  |
| **3439** | **IFNα** | interferon, alpha 1 | hsa04060:Cytokine-cytokine receptor interaction,hsa04140:Regulation of autophagy,hsa04612:Antigen processing and presentation,hsa04620:Toll-like receptor signaling pathway,hsa04622:RIG-I-like receptor signaling pathway,hsa04623:Cytosolic DNA-sensing pathway,hsa04630:Jak-STAT signaling pathway,hsa04650:Natural killer cell mediated cytotoxicity,hsa05320:Autoimmune thyroid disease, |
| **4841** | **NONO** | non-POU domain containing, octamer-binding |  |
| **4862** | **NPAS2** | neuronal PAS domain protein 2 | hsa04710:Circadian rhythm, |
| **2033** | **p300** | E1A binding protein p300 | hsa04110:Cell cycle,hsa04310:Wnt signaling pathway,hsa04330:Notch signaling pathway,hsa04350:TGF-beta signaling pathway,hsa04520:Adherens junction,hsa04630:Jak-STAT signaling pathway,hsa04720:Long-term potentiation,hsa04916:Melanogenesis,hsa05016:Huntington's disease,hsa05200:Pathways in cancer,hsa05211:Renal cell carcinoma,hsa05215:Prostate cancer, |
| **142** | **PARP1** | poly (ADP-ribose) polymerase 1 | hsa03410:Base excision repair, |
| **5187** | **PER1** | period homolog 1 (Drosophila) | hsa04710:Circadian rhythm, |
| **8864** | **PER2** | period homolog 2 (Drosophila) | hsa04710:Circadian rhythm, |
| **8863** | **PER3** | period homolog 3 (Drosophila) | hsa04710:Circadian rhythm, |
| **5465** | **PPARα** | peroxisome proliferator-activated receptor alpha | hsa03320:PPAR signaling pathway,hsa04920:Adipocytokine signaling pathway, |
| **5468** | **PPARγ** | peroxisome proliferator-activated receptor gamma | hsa03320:PPAR signaling pathway,hsa05016:Huntington's disease,hsa05200:Pathways in cancer,hsa05216:Thyroid cancer, |
| **5578** | **PRKC-α** | protein kinase C, alpha | MAPK signaling pathway, ErbB signaling pathway, Calcium signaling pathway, Phosphatidylinositol signaling system, Vascular smooth muscle contraction, Wnt signaling pathway, VEGF signaling pathway, Focal adhesion, Tight junction, Gap junction, Natural killer cell mediated cytotoxicity, Fc epsilon RI signaling pathway, Fc gamma R-mediated phagocytosis, Leukocyte transendothelial migration, Long-term potentiation, Long-term depression, GnRH signaling pathway, Melanogenesis, Aldosterone-regulated sodium reabsorption, Vibrio cholerae infection, Pathogenic Escherichia coli infection, Pathways in cancer, Glioma, Non-small cell lung cancer, |
| **10399** | **RACK1** | guanine nucleotide binding protein (G protein), beta polypeptide 2-like 1 |  |
| **9572** | **REV-ERBα** | nuclear receptor subfamily 1, group D, member 1 | hsa04710:Circadian rhythm, |
| **9975** | **REV-ERBβ** | nuclear receptor subfamily 1, group D, member 2 |  |
| **6095** | **RORα** | RAR-related orphan receptor A |  |
| **6096** | **RORβ** | RAR-related orphan receptor B |  |
| **6097** | **RORγ** | RAR-related orphan receptor C |  |
| **23411** | **SIRT1** | sirtuin (silent mating type information regulation 2 homolog) 1 (S. cerevisiae) |  |
| **7008** | **TEF** | thyrotrophic embryonic factor |  |
| **7124** | **TNFα** | tumor necrosis factor (TNF superfamily, member 2) | hsa04010:MAPK signaling pathway,hsa04060:Cytokine-cytokine receptor interaction,hsa04210:Apoptosis,hsa04350:TGF-beta signaling pathway,hsa04620:Toll-like receptor signaling pathway,hsa04621:NOD-like receptor signaling pathway,hsa04622:RIG-I-like receptor signaling pathway,hsa04640:Hematopoietic cell lineage,hsa04650:Natural killer cell mediated cytotoxicity,hsa04660:T cell receptor signaling pathway,hsa04664:Fc epsilon RI signaling pathway,hsa04920:Adipocytokine signaling pathway,hsa04930:Type II diabetes mellitus,hsa04940:Type I diabetes mellitus,hsa05010:Alzheimer's disease,hsa05014:Amyotrophic lateral sclerosis (ALS),hsa05310:Asthma,hsa05322:Systemic lupus erythematosus,hsa05330:Allograft rejection,hsa05332:Graft-versus-host disease,hsa05410:Hypertrophic cardiomyopathy (HCM),hsa05414:Dilated cardiomyopathy, |
| **11091** | **WDR5** | WD repeat domain 5 |  |

**Table 1.** List of clock and clock-associated genes

| **Interaction** | **Reference** |
| --- | --- |
| **AhR - BMAL1** | (Xu et al., 2010) |
| **AhR - CREB** | (Gomez-Duran et al., 2008; Hashimoto et al., 2009; Reymann and Borlak, 2006) |
| **AhR - PER1,2** | (Claudel et al., 2007; Garrett and Gasiewicz, 2006; Mukai and Tischkau, 2007) |
| **AhR - PPARα, γ** | (Cimafranca et al., 2004; Lovekamp-Swan et al., 2003; Shaban et al., 2004; Villard et al., 2007) |
| **AhR - TNFα** | (Ke et al., 2001; Kobayashi et al., 2008)13, |
| **AhR - β-TRCP** | (Gluschnaider et al., 2010) |
| **ALAS1 - NPAS2** | (Pardee et al., 2009; Yin et al., 2010) |
| **ALAS1 - PPARα** | (Degenhardt et al., 2009) |
| **AMPK - CRY1** | (Lamia et al., 2009) |
| **AMPK - PPARα** | (Yuan et al., 2010) |
| **BMAL1 - BMAL2** | (Shi et al., 2010) |
| **BMAL1 - CLOCK** | (Baggs et al., 2009) |
| **BMAL1 - CRY1, 2** | (Chen et al., 2009; Etchegaray et al., 2003; Ko and Takahashi, 2006; Langmesser et al., 2008; Shearman et al., 2000) |
| **BMAL1 - DBP** | (Ripperger and Schibler, 2006) |
| **BMAL1 - NPAS** | (Takahashi et al., 2008) |
| **BMAL1 - PER3** | (Chen et al., 2009; Ko and Takahashi, 2006; Langmesser et al., 2008; Sasaki et al., 2009; Shearman et al., 2000) |
| **BMAL1 - PKCα** | (Robles et al., 2010) |
| **BMAL1 - PPARα, γ** | (Teboul et al., 2008; Wang et al., 2008) |
| **BMAL1 - RACK1** | (Robles et al.) |
| **BMAL1 - REV-ERBα, β** | (Guillaumond et al., 2005; Preitner et al., 2002) |
| **BMAL1 - RORα, β, γ** | (Guillaumond et al., 2005) |
| **BMAL1 - SIRT1** | (Asher et al., 2008) |
| **BMAL1, 2 - IFNα** | (Ohdo et al., 2001) |
| **BMAL2 - CLOCK** | (Sasaki et al., 2009) |
| **BMAL2 - CRY1** | (Dardente et al., 2007; Lavebratt et al., 2010) |
| **BMAL1, 2 - PER1, 2** | (Chen et al., 2009; Ko and Takahashi, 2006; Langmesser et al., 2008; Sasaki et al., 2009; Shearman et al., 2000) |
| **bTRCP - p300** | (Kimbrel and Kung, 2009) |
| **bTRCP - PER1, 2, 3** | (Takahashi et al., 2008) |
| **CAR - DBP** | (Gachon et al., 2006) |
| **CAR - HLF** | (Gachon et al., 2006) |
| **CAR - TEF** | (Gachon et al., 2006) |
| **CBP - CLOCK** | (Etchegaray et al., 2003; Hung et al., 2007; Lee et al., 2010) |
| **CBP - CREB** | (Campbell and Lumb, 2002; Chaudhary and Skinner, 2001; Vendel et al., 2003) |
| **CBP - IFN-α** | (Plath et al., 1999) |
| **CBP - p300** | (Etchegaray et al., 2003; Hung et al., 2007; Lee et al., 2010) |
| **CBP - PARP-1** | (Zerfaoui et al., 2008) |
| **CBP - PPARα, β, γ** | (Blanquart et al., 2004; Majdalawieh and Ro, 2010; Powell et al., 2007; Sugawara et al., 2003; Tolon et al., 1998) |
| **CBP - SIRT1** | (MacIsaac et al., 2010) |
| **CLOCK - CRY1, 2** | (Chen et al., 2009; Etchegaray et al., 2003; Ko and Takahashi, 2006; Langmesser et al., 2008) |
| **CLOCK - DBP** | (Ripperger and Schibler, 2006) |
| **CLOCK - GSK-3β** | (Spengler et al., 2009) |
| **CLOCK - IFNα** | (Ohdo et al., 2001) |
| **CLOCK - p300** | (Etchegaray et al., 2003; Hung et al., 2007; Lee et al., 2010) |
| **CLOCK - PARP-1** | (Asher et al., 2010) |
| **CLOCK - PER1, 2, 3** | (Gekakis et al., 1998; Hennig et al., 2009; Sasaki et al., 2009; Shearman et al., 2000) |
| **CLOCK - REV-ERBα** | (Preitner et al., 2002) |
| **CLOCK - RORα** | (Ueda et al., 2002) |
| **CREB - CREB** | (Ahn et al., 1998) |
| **CREB - GSK-3β** | (El Jamali et al., 2004; Kaidanovich-Beilin and Eldar-Finkelman, 2006; Tyson et al., 2002) |
| **CREB - NONO** | (Amelio et al., 2007) |
| **CREB - p300** | (Chaudhary and Skinner, 2001; Lee et al., 1996; Solomou et al., 2001) |
| **CREB - PER1, 2, 3** | (Levi and Schibler, 2007) |
| **CREB - PPARα, β, γ** | (Beaumont et al., 2008; Herzig et al., 2003; Scoditti et al., 2009; Zhou et al., 2007) |
| **CREB - TNFα** | (Avni et al., 2009; Ono et al., 2004) |
| **CRY1 - DBP** | (Stratmann et al., 2010) |
| **CRY1 - REV-ERBα, β** | (Liu et al., 2008) |
| **CRY1, 2 - CSNK1ε, ** | (Takahashi et al., 2008) |
| **CRY1, 2 - FBXL3** | (Takahashi et al., 2008; Zhang and Kay, 2010) |
| **CRY1, 2 - NPAS2** | (Lavebratt et al., 2010; Pendergast et al., 2010; Wisor et al., 2002) |
| **CRY1, 2 - PER1, 2** | (Chen et al., 2009; Daan et al., 2001; Ko and Takahashi, 2006; Langmesser et al., 2008; Ozber et al., 2010; Yang et al., 2008) |
| **CRY2 - E4BP4** | (Ohno et al., 2007a) |
| **CRY2 - GSK-3β** | (Rana and Mahmood, 2010) |
| **CSNK1δ, ε- PER1, 2, 3** | (Takahashi et al., 2008; Zhang and Kay, 2010) |
| **CSNK2α - PER1, 2, 3** | (Zhang and Kay, 2010) |
| **DBP - PER1, 2, 3** | (Hastings et al., 2007) |
| **DBP - PPARα** | (Gachon et al., 2011; Gachon et al., 2006) |
| **DBP - TNFα** | (Cavadini et al., 2007) |
| **DEC1, 2 - PER1** | (Li et al., 2004) |
| **E4BP4 - PER2** | (Ohno et al., 2007a, b) |
| **GSK-3β - REV-ERBα** | (Rana and Mahmood, 2010) |
| **GSK-3β - PER2** | (Iitaka et al., 2005) |
| **GSK-3β - PPARα, γ** | (Li et al., 2007) (Farmer, 2005) |
| **GSK-3β - TNFα** | (Kramer et al., 2009) (Oguma et al., 2008) |
| **HLF - PER1, 2, 3** | (Hastings et al., 2007) |
| **HLF - PPARα** | (Gachon et al., 2011; Gachon et al., 2006) |
| **HLF - TNFα** | (Cavadini et al., 2007) |
| **IFNα - RACK1** | (Usacheva et al., 2001; Usacheva et al., 2003) |
| **IFN-α - RORγ** | (Hirohata et al., 2009) |
| **IFN-α - TNFα** | (Jansen et al., 1992; Szabo et al., 2001) |
| **NONO - TNFα** | (Stier et al., 2005) |
| **NONO - PER1** | (Brown et al., 2005; Kowalska et al., 2012) |
| **NPAS2 - p300** | (Curtis et al., 2004) |
| **NPAS2 - PER1,2,3** | (Etchegaray et al., 2010; Franken et al., 2006; Kondratova et al., 2010; Pendergast et al., 2010) |
| **NPAS2 - RORγ** | (Liu et al., 2008) |
| **p300 - PARP-1** | (Zerfaoui et al., 2008) |
| **p300 - PPARα, γ** | (Cho et al., 2009; Dowell et al., 1999; Gelman et al., 1999; Majdalawieh and Ro; Misra et al., 2002; Powell et al., 2007; Sugawara et al., 2003; Wang et al., 2001) |
| **p300 - RORα** | (Lau et al., 1999) |
| **p300 - SIRT1** | (Bourguignon et al., 2009) |
| **p300 - TNFα** | (Deng et al., 2003; Granja et al., 2006; Yu et al., 2009) |
| **PARP-1 - SIRT1** | (Rajamohan et al., 2009) |
| **PARP-1 - TNFα** | (Mathieu et al., 2008) |
| **PER1 - RACK1** | (Hu et al., 2006) |
| **PER1 - REV-ERBα, β** | (Baggs et al., 2009) |
| **PER1 - WDR5** | (Brown et al., 2005) |
| **PER1, 2 - PER3** | (Hennig et al., 2009; Lee et al., 2004; Loop and Pieler, 2005; Yagita et al., 2000) |
| **PER1, 2, 3 - TEF** | (Hastings et al., 2007) |
| **PER1, 2, 3 - TNFα** | (Cavadini et al., 2007) |
| **PER2 - PPARα** | (Schmutz et al., 2010) |
| **PER2 - REV-ERBα, β** | (Cheng et al., 2005; Cho et al., 2012; Preitner et al., 2002; Ueda et al., 2002) |
| **PKCα - RACK1** | (Robles et al.) |
| **PPARα - TEF** | (Gachon et al., 2011; Gachon et al., 2006) |
| **PPARα, γ - SIRT1** | (Canto and Auwerx, 2011; Das, 2005; Laule et al., 2006; Picard et al., 2004; Purushotham et al., 2009) |
| **PPARα, γ - RORα** | (Tordjman et al., 2007) |
| **PPARα, γ - TNFα** | (Beier et al., 1997; Lee et al., 2009; Rosenbaum and Greenberg, 1998) |
| **PPARγ - REV-ERBα** | (Duez and Staels, 2008) |
| **PPARγ- CK2 (CSNK1E)** | (von Knethen et al., 2009) |
| **REV-ERBα, β - RORα, γ** | (Lin et al., 2009; Liu et al., 2008) |
| **RORα, γ - TNFα** | (Migita and Morser, 2005; Migita et al., 2004) |
| **SIRT1 - TNFα** | (Yoshizaki et al., 2009) (Zhang et al., 2010) |
| **TEF - TNFα** | (Cavadini et al., 2007) |

**Table2.** Full list of interactions present in the regulatory network of the mammalian circadian clock (Figure2A)

**References**

Ahn, S., Olive, M., Aggarwal, S., Krylov, D., Ginty, D.D., and Vinson, C. (1998). A dominant-negative inhibitor of CREB reveals that it is a general mediator of stimulus-dependent transcription of c-fos. Mol Cell Biol *18*, 967-977.

Amelio, A.L., Miraglia, L.J., Conkright, J.J., Mercer, B.A., Batalov, S., Cavett, V., Orth, A.P., Busby, J., Hogenesch, J.B., and Conkright, M.D. (2007). A coactivator trap identifies NONO (p54nrb) as a component of the cAMP-signaling pathway. Proc Natl Acad Sci U S A *104*, 20314-20319.

Asher, G., Gatfield, D., Stratmann, M., Reinke, H., Dibner, C., Kreppel, F., Mostoslavsky, R., Alt, F.W., and Schibler, U. (2008). SIRT1 regulates circadian clock gene expression through PER2 deacetylation. Cell *134*, 317-328.

Asher, G., Reinke, H., Altmeyer, M., Gutierrez-Arcelus, M., Hottiger, M.O., and Schibler, U. (2010). Poly(ADP-ribose) polymerase 1 participates in the phase entrainment of circadian clocks to feeding. Cell *142*, 943-953.

Avni, D., Philosoph, A., Meijler, M.M., and Zor, T. (2009). The ceramide-1-phosphate analogue PCERA-1 modulates tumour necrosis factor-alpha and interleukin-10 production in macrophages via the cAMP-PKA-CREB pathway in a GTP-dependent manner. Immunology *129*, 375-385.

Baggs, J.E., Price, T.S., DiTacchio, L., Panda, S., Fitzgerald, G.A., and Hogenesch, J.B. (2009). Network features of the mammalian circadian clock. PLoS Biol *7*, e52.

Beaumont, J., Arias, T., Ravassa, S., and Diez, J. (2008). Overexpression of human truncated peroxisome proliferator-activated receptor alpha induces apoptosis in HL-1 cardiomyocytes. Cardiovasc Res *79*, 458-463.

Beier, K., Volkl, A., and Fahimi, H.D. (1997). TNF-alpha downregulates the peroxisome proliferator activated receptor-alpha and the mRNAs encoding peroxisomal proteins in rat liver. FEBS Lett *412*, 385-387.

Blanquart, C., Mansouri, R., Fruchart, J.C., Staels, B., and Glineur, C. (2004). Different ways to regulate the PPARalpha stability. Biochem Biophys Res Commun *319*, 663-670.

Bourguignon, L.Y., Xia, W., and Wong, G. (2009). Hyaluronan-mediated CD44 interaction with p300 and SIRT1 regulates beta-catenin signaling and NFkappaB-specific transcription activity leading to MDR1 and Bcl-xL gene expression and chemoresistance in breast tumor cells. J Biol Chem *284*, 2657-2671.

Brown, S.A., Ripperger, J., Kadener, S., Fleury-Olela, F., Vilbois, F., Rosbash, M., and Schibler, U. (2005). PERIOD1-associated proteins modulate the negative limb of the mammalian circadian oscillator. Science *308*, 693-696.

Campbell, K.M., and Lumb, K.J. (2002). Structurally distinct modes of recognition of the KIX domain of CBP by Jun and CREB. Biochemistry *41*, 13956-13964.

Canto, C., and Auwerx, J. (2011). Targeting sirtuin 1 to improve metabolism: all you need is NAD(+)? Pharmacol Rev *64*, 166-187.

Cavadini, G., Petrzilka, S., Kohler, P., Jud, C., Tobler, I., Birchler, T., and Fontana, A. (2007). TNF-alpha suppresses the expression of clock genes by interfering with E-box-mediated transcription. Proc Natl Acad Sci U S A *104*, 12843-12848.

Chaudhary, J., and Skinner, M.K. (2001). Role of the transcriptional coactivator CBP/p300 in linking basic helix-loop-helix and CREB responses for follicle-stimulating hormone-mediated activation of the transferrin promoter in Sertoli cells. Biol Reprod *65*, 568-574.

Chen, R., Schirmer, A., Lee, Y., Lee, H., Kumar, V., Yoo, S.H., Takahashi, J.S., and Lee, C. (2009). Rhythmic PER abundance defines a critical nodal point for negative feedback within the circadian clock mechanism. Mol Cell *36*, 417-430.

Cheng, M.Y., Bittman, E.L., Hattar, S., and Zhou, Q.Y. (2005). Regulation of prokineticin 2 expression by light and the circadian clock. BMC Neurosci *6*, 17.

Cho, H., Zhao, X., Hatori, M., Yu, R.T., Barish, G.D., Lam, M.T., Chong, L.W., DiTacchio, L., Atkins, A.R., Glass, C.K.*, et al.* (2012). Regulation of circadian behaviour and metabolism by REV-ERB-alpha and REV-ERB-beta. Nature *485*, 123-127.

Cho, M.C., Lee, S., Choi, H.S., Yang, Y., Tae Hong, J., Kim, S.J., and Yoon, D.Y. (2009). Optimization of an enzyme-linked immunosorbent assay to screen ligand of Peroxisome proliferator-activated receptor alpha. Immunopharmacol Immunotoxicol *31*, 459-467.

Cimafranca, M.A., Hanlon, P.R., and Jefcoate, C.R. (2004). TCDD administration after the pro-adipogenic differentiation stimulus inhibits PPARgamma through a MEK-dependent process but less effectively suppresses adipogenesis. Toxicol Appl Pharmacol *196*, 156-168.

Claudel, T., Cretenet, G., Saumet, A., and Gachon, F. (2007). Crosstalk between xenobiotics metabolism and circadian clock. FEBS Lett *581*, 3626-3633.

Curtis, A.M., Seo, S.B., Westgate, E.J., Rudic, R.D., Smyth, E.M., Chakravarti, D., FitzGerald, G.A., and McNamara, P. (2004). Histone acetyltransferase-dependent chromatin remodeling and the vascular clock. J Biol Chem *279*, 7091-7097.

Daan, S., Albrecht, U., van der Horst, G.T., Illnerova, H., Roenneberg, T., Wehr, T.A., and Schwartz, W.J. (2001). Assembling a clock for all seasons: are there M and E oscillators in the genes? J Biol Rhythms *16*, 105-116.

Dardente, H., Fortier, E.E., Martineau, V., and Cermakian, N. (2007). Cryptochromes impair phosphorylation of transcriptional activators in the clock: a general mechanism for circadian repression. Biochem J *402*, 525-536.

Das, U.N. (2005). A defect in the activity of Delta6 and Delta5 desaturases may be a factor predisposing to the development of insulin resistance syndrome. Prostaglandins Leukot Essent Fatty Acids *72*, 343-350.

Degenhardt, T., Vaisanen, S., Rakhshandehroo, M., Kersten, S., and Carlberg, C. (2009). Peroxisome proliferator-activated receptor alpha controls hepatic heme biosynthesis through ALAS1. J Mol Biol *388*, 225-238.

Deng, W.G., Zhu, Y., and Wu, K.K. (2003). Up-regulation of p300 binding and p50 acetylation in tumor necrosis factor-alpha-induced cyclooxygenase-2 promoter activation. J Biol Chem *278*, 4770-4777.

Dowell, P., Ishmael, J.E., Avram, D., Peterson, V.J., Nevrivy, D.J., and Leid, M. (1999). Identification of nuclear receptor corepressor as a peroxisome proliferator-activated receptor alpha interacting protein. J Biol Chem *274*, 15901-15907.

Duez, H., and Staels, B. (2008). Rev-erb alpha gives a time cue to metabolism. FEBS Lett *582*, 19-25.

El Jamali, A., Freund, C., Rechner, C., Scheidereit, C., Dietz, R., and Bergmann, M.W. (2004). Reoxygenation after severe hypoxia induces cardiomyocyte hypertrophy in vitro: activation of CREB downstream of GSK3beta. FASEB J *18*, 1096-1098.

Etchegaray, J.P., Lee, C., Wade, P.A., and Reppert, S.M. (2003). Rhythmic histone acetylation underlies transcription in the mammalian circadian clock. Nature *421*, 177-182.

Etchegaray, J.P., Yu, E.A., Indic, P., Dallmann, R., and Weaver, D.R. (2010). Casein kinase 1 delta (CK1delta) regulates period length of the mouse suprachiasmatic circadian clock in vitro. PLoS One *5*, e10303.

Farmer, S.R. (2005). Regulation of PPARgamma activity during adipogenesis. Int J Obes (Lond) *29 Suppl 1*, S13-16.

Franken, P., Dudley, C.A., Estill, S.J., Barakat, M., Thomason, R., O'Hara, B.F., and McKnight, S.L. (2006). NPAS2 as a transcriptional regulator of non-rapid eye movement sleep: genotype and sex interactions. Proc Natl Acad Sci U S A *103*, 7118-7123.

Gachon, F., Leuenberger, N., Claudel, T., Gos, P., Jouffe, C., Fleury Olela, F., de Mollerat du Jeu, X., Wahli, W., and Schibler, U. (2011). Proline- and acidic amino acid-rich basic leucine zipper proteins modulate peroxisome proliferator-activated receptor alpha (PPARalpha) activity. Proc Natl Acad Sci U S A *108*, 4794-4799.

Gachon, F., Olela, F.F., Schaad, O., Descombes, P., and Schibler, U. (2006). The circadian PAR-domain basic leucine zipper transcription factors DBP, TEF, and HLF modulate basal and inducible xenobiotic detoxification. Cell Metab *4*, 25-36.

Garrett, R.W., and Gasiewicz, T.A. (2006). The aryl hydrocarbon receptor agonist 2,3,7,8-tetrachlorodibenzo-p-dioxin alters the circadian rhythms, quiescence, and expression of clock genes in murine hematopoietic stem and progenitor cells. Mol Pharmacol *69*, 2076-2083.

Gekakis, N., Staknis, D., Nguyen, H.B., Davis, F.C., Wilsbacher, L.D., King, D.P., Takahashi, J.S., and Weitz, C.J. (1998). Role of the CLOCK protein in the mammalian circadian mechanism. Science *280*, 1564-1569.

Gelman, L., Zhou, G., Fajas, L., Raspe, E., Fruchart, J.C., and Auwerx, J. (1999). p300 interacts with the N- and C-terminal part of PPARgamma2 in a ligand-independent and -dependent manner, respectively. J Biol Chem *274*, 7681-7688.

Gluschnaider, U., Hidas, G., Cojocaru, G., Yutkin, V., Ben-Neriah, Y., and Pikarsky, E. (2010). beta-TrCP inhibition reduces prostate cancer cell growth via upregulation of the aryl hydrocarbon receptor. PLoS One *5*, e9060.

Gomez-Duran, A., Ballestar, E., Carvajal-Gonzalez, J.M., Marlowe, J.L., Puga, A., Esteller, M., and Fernandez-Salguero, P.M. (2008). Recruitment of CREB1 and histone deacetylase 2 (HDAC2) to the mouse Ltbp-1 promoter regulates its constitutive expression in a dioxin receptor-dependent manner. J Mol Biol *380*, 1-16.

Granja, A.G., Nogal, M.L., Hurtado, C., Del Aguila, C., Carrascosa, A.L., Salas, M.L., Fresno, M., and Revilla, Y. (2006). The viral protein A238L inhibits TNF-alpha expression through a CBP/p300 transcriptional coactivators pathway. J Immunol *176*, 451-462.

Guillaumond, F., Dardente, H., Giguere, V., and Cermakian, N. (2005). Differential control of Bmal1 circadian transcription by REV-ERB and ROR nuclear receptors. J Biol Rhythms *20*, 391-403.

Hashimoto, Y., Loftis, D.W., and Adams, J.C. (2009). Fascin-1 promoter activity is regulated by CREB and the aryl hydrocarbon receptor in human carcinoma cells. PLoS One *4*, e5130.

Hastings, M., O'Neill, J.S., and Maywood, E.S. (2007). Circadian clocks: regulators of endocrine and metabolic rhythms. J Endocrinol *195*, 187-198.

Hennig, S., Strauss, H.M., Vanselow, K., Yildiz, O., Schulze, S., Arens, J., Kramer, A., and Wolf, E. (2009). Structural and functional analyses of PAS domain interactions of the clock proteins Drosophila PERIOD and mouse PERIOD2. PLoS Biol *7*, e94.

Herzig, S., Hedrick, S., Morantte, I., Koo, S.H., Galimi, F., and Montminy, M. (2003). CREB controls hepatic lipid metabolism through nuclear hormone receptor PPAR-gamma. Nature *426*, 190-193.

Hirohata, S., Shibuya, H., and Tejima, S. (2009). Suppressive influences of IFN-alpha on IL-17 expression in human CD4+ T cells. Clin Immunol *134*, 340-344.

Hu, L., Lu, F., Wang, Y., Liu, Y., Liu, D., Jiang, Z., Wan, C., Zhu, B., Gan, L., and Wang, Z. (2006). RACK1, a novel hPER1-interacting protein. J Mol Neurosci *29*, 55-63.

Hung, H.C., Maurer, C., Kay, S.A., and Weber, F. (2007). Circadian transcription depends on limiting amounts of the transcription co-activator nejire/CBP. J Biol Chem *282*, 31349-31357.

Iitaka, C., Miyazaki, K., Akaike, T., and Ishida, N. (2005). A role for glycogen synthase kinase-3beta in the mammalian circadian clock. J Biol Chem *280*, 29397-29402.

Jansen, J.H., Wientjens, G.J., Willemze, R., and Kluin-Nelemans, J.C. (1992). Production of tumor necrosis factor-alpha by normal and malignant B lymphocytes in response to interferon-alpha, interferon-gamma and interleukin-4. Leukemia *6*, 116-119.

Kaidanovich-Beilin, O., and Eldar-Finkelman, H. (2006). Long-term treatment with novel glycogen synthase kinase-3 inhibitor improves glucose homeostasis in ob/ob mice: molecular characterization in liver and muscle. J Pharmacol Exp Ther *316*, 17-24.

Ke, S., Rabson, A.B., Germino, J.F., Gallo, M.A., and Tian, Y. (2001). Mechanism of suppression of cytochrome P-450 1A1 expression by tumor necrosis factor-alpha and lipopolysaccharide. J Biol Chem *276*, 39638-39644.

Kimbrel, E.A., and Kung, A.L. (2009). The F-box protein beta-TrCp1/Fbw1a interacts with p300 to enhance beta-catenin transcriptional activity. J Biol Chem *284*, 13033-13044.

Ko, C.H., and Takahashi, J.S. (2006). Molecular components of the mammalian circadian clock. Hum Mol Genet *15 Spec No 2*, R271-277.

Kobayashi, S., Okamoto, H., Iwamoto, T., Toyama, Y., Tomatsu, T., Yamanaka, H., and Momohara, S. (2008). A role for the aryl hydrocarbon receptor and the dioxin TCDD in rheumatoid arthritis. Rheumatology (Oxford) *47*, 1317-1322.

Kondratova, A.A., Dubrovsky, Y.V., Antoch, M.P., and Kondratov, R.V. (2010). Circadian clock proteins control adaptation to novel environment and memory formation. Aging (Albany NY) *2*, 285-297.

Kowalska, E., Ripperger, J.A., Hoegger, D.C., Bruegger, P., Buch, T., Birchler, T., Mueller, A., Albrecht, U., Contaldo, C., and Brown, S.A. (2012). Feature Article: NONO couples the circadian clock to the cell cycle. Proc Natl Acad Sci U S A *110*, 1592-1599.

Kramer, P.R., Winger, V., and Reuben, J. (2009). PI3K limits TNF-alpha production in CD16-activated monocytes. Eur J Immunol *39*, 561-570.

Lamia, K.A., Sachdeva, U.M., DiTacchio, L., Williams, E.C., Alvarez, J.G., Egan, D.F., Vasquez, D.S., Juguilon, H., Panda, S., Shaw, R.J.*, et al.* (2009). AMPK regulates the circadian clock by cryptochrome phosphorylation and degradation. Science *326*, 437-440.

Langmesser, S., Tallone, T., Bordon, A., Rusconi, S., and Albrecht, U. (2008). Interaction of circadian clock proteins PER2 and CRY with BMAL1 and CLOCK. BMC Mol Biol *9*, 41.

Lau, P., Bailey, P., Dowhan, D.H., and Muscat, G.E. (1999). Exogenous expression of a dominant negative RORalpha1 vector in muscle cells impairs differentiation: RORalpha1 directly interacts with p300 and myoD. Nucleic Acids Res *27*, 411-420.

Laule, O., Hirsch-Hoffmann, M., Hruz, T., Gruissem, W., and Zimmermann, P. (2006). Web-based analysis of the mouse transcriptome using Genevestigator. BMC Bioinformatics *7*, 311.

Lavebratt, C., Sjoholm, L.K., Soronen, P., Paunio, T., Vawter, M.P., Bunney, W.E., Adolfsson, R., Forsell, Y., Wu, J.C., Kelsoe, J.R.*, et al.* (2010). CRY2 is associated with depression. PLoS One *5*, e9407.

Lee, C., Weaver, D.R., and Reppert, S.M. (2004). Direct association between mouse PERIOD and CKIepsilon is critical for a functioning circadian clock. Mol Cell Biol *24*, 584-594.

Lee, J.S., Zhang, X., and Shi, Y. (1996). Differential interactions of the CREB/ATF family of transcription factors with p300 and adenovirus E1A. J Biol Chem *271*, 17666-17674.

Lee, T.I., Kao, Y.H., Chen, Y.C., and Chen, Y.J. (2009). Proinflammatory cytokine and ligands modulate cardiac peroxisome proliferator-activated receptors. Eur J Clin Invest *39*, 23-30.

Lee, Y., Lee, J., Kwon, I., Nakajima, Y., Ohmiya, Y., Son, G.H., Lee, K.H., and Kim, K. (2010). Coactivation of the CLOCK-BMAL1 complex by CBP mediates resetting of the circadian clock. J Cell Sci *123*, 3547-3557.

Levi, F., and Schibler, U. (2007). Circadian rhythms: mechanisms and therapeutic implications. Annu Rev Pharmacol Toxicol *47*, 593-628.

Li, R., Zheng, W., Pi, R., Gao, J., Zhang, H., Wang, P., Le, K., and Liu, P. (2007). Activation of peroxisome proliferator-activated receptor-alpha prevents glycogen synthase 3beta phosphorylation and inhibits cardiac hypertrophy. FEBS Lett *581*, 3311-3316.

Li, Y., Song, X., Ma, Y., Liu, J., Yang, D., and Yan, B. (2004). DNA binding, but not interaction with Bmal1, is responsible for DEC1-mediated transcription regulation of the circadian gene mPer1. Biochem J *382*, 895-904.

Lin, K.K., Kumar, V., Geyfman, M., Chudova, D., Ihler, A.T., Smyth, P., Paus, R., Takahashi, J.S., and Andersen, B. (2009). Circadian clock genes contribute to the regulation of hair follicle cycling. PLoS Genet *5*, e1000573.

Liu, A.C., Tran, H.G., Zhang, E.E., Priest, A.A., Welsh, D.K., and Kay, S.A. (2008). Redundant function of REV-ERBalpha and beta and non-essential role for Bmal1 cycling in transcriptional regulation of intracellular circadian rhythms. PLoS Genet *4*, e1000023.

Loop, S., and Pieler, T. (2005). Nuclear import of mPER3 in Xenopus oocytes and HeLa cells requires complex formation with mPER1. FEBS J *272*, 3714-3724.

Lovekamp-Swan, T., Jetten, A.M., and Davis, B.J. (2003). Dual activation of PPARalpha and PPARgamma by mono-(2-ethylhexyl) phthalate in rat ovarian granulosa cells. Mol Cell Endocrinol *201*, 133-141.

MacIsaac, K.D., Lo, K.A., Gordon, W., Motola, S., Mazor, T., and Fraenkel, E. (2010). A quantitative model of transcriptional regulation reveals the influence of binding location on expression. PLoS Comput Biol *6*, e1000773.

Majdalawieh, A., and Ro, H.S. PPARgamma1 and LXRalpha face a new regulator of macrophage cholesterol homeostasis and inflammatory responsiveness, AEBP1. Nucl Recept Signal *8*, e004.

Majdalawieh, A., and Ro, H.S. (2010). PPARgamma1 and LXRalpha face a new regulator of macrophage cholesterol homeostasis and inflammatory responsiveness, AEBP1. Nucl Recept Signal *8*, e004.

Mathieu, J., Flexor, M., Lanotte, M., and Besancon, F. (2008). A PARP-1/JNK1 cascade participates in the synergistic apoptotic effect of TNFalpha and all-trans retinoic acid in APL cells. Oncogene *27*, 3361-3370.

Migita, H., and Morser, J. (2005). 15-deoxy-Delta12,14-prostaglandin J2 (15d-PGJ2) signals through retinoic acid receptor-related orphan receptor-alpha but not peroxisome proliferator-activated receptor-gamma in human vascular endothelial cells: the effect of 15d-PGJ2 on tumor necrosis factor-alpha-induced gene expression. Arterioscler Thromb Vasc Biol *25*, 710-716.

Migita, H., Satozawa, N., Lin, J.H., Morser, J., and Kawai, K. (2004). RORalpha1 and RORalpha4 suppress TNF-alpha-induced VCAM-1 and ICAM-1 expression in human endothelial cells. FEBS Lett *557*, 269-274.

Misra, P., Qi, C., Yu, S., Shah, S.H., Cao, W.Q., Rao, M.S., Thimmapaya, B., Zhu, Y., and Reddy, J.K. (2002). Interaction of PIMT with transcriptional coactivators CBP, p300, and PBP differential role in transcriptional regulation. J Biol Chem *277*, 20011-20019.

Mukai, M., and Tischkau, S.A. (2007). Effects of tryptophan photoproducts in the circadian timing system: searching for a physiological role for aryl hydrocarbon receptor. Toxicol Sci *95*, 172-181.

Oguma, K., Oshima, H., Aoki, M., Uchio, R., Naka, K., Nakamura, S., Hirao, A., Saya, H., Taketo, M.M., and Oshima, M. (2008). Activated macrophages promote Wnt signalling through tumour necrosis factor-alpha in gastric tumour cells. EMBO J *27*, 1671-1681.

Ohdo, S., Koyanagi, S., Suyama, H., Higuchi, S., and Aramaki, H. (2001). Changing the dosing schedule minimizes the disruptive effects of interferon on clock function. Nat Med *7*, 356-360.

Ohno, T., Onishi, Y., and Ishida, N. (2007a). The negative transcription factor E4BP4 is associated with circadian clock protein PERIOD2. Biochem Biophys Res Commun *354*, 1010-1015.

Ohno, T., Onishi, Y., and Ishida, N. (2007b). A novel E4BP4 element drives circadian expression of mPeriod2. Nucleic Acids Res *35*, 648-655.

Ono, H., Ichiki, T., Fukuyama, K., Iino, N., Masuda, S., Egashira, K., and Takeshita, A. (2004). cAMP-response element-binding protein mediates tumor necrosis factor-alpha-induced vascular smooth muscle cell migration. Arterioscler Thromb Vasc Biol *24*, 1634-1639.

Ozber, N., Baris, I., Tatlici, G., Gur, I., Kilinc, S., Unal, E.B., and Kavakli, I.H. (2010). Identification of two amino acids in the C-terminal domain of mouse CRY2 essential for PER2 interaction. BMC Mol Biol *11*, 69.

Pardee, K.I., Xu, X., Reinking, J., Schuetz, A., Dong, A., Liu, S., Zhang, R., Tiefenbach, J., Lajoie, G., Plotnikov, A.N.*, et al.* (2009). The structural basis of gas-responsive transcription by the human nuclear hormone receptor REV-ERBbeta. PLoS Biol *7*, e43.

Pendergast, J.S., Friday, R.C., and Yamazaki, S. (2010). Distinct functions of Period2 and Period3 in the mouse circadian system revealed by in vitro analysis. PLoS One *5*, e8552.

Picard, F., Kurtev, M., Chung, N., Topark-Ngarm, A., Senawong, T., Machado De Oliveira, R., Leid, M., McBurney, M.W., and Guarente, L. (2004). Sirt1 promotes fat mobilization in white adipocytes by repressing PPAR-gamma. Nature *429*, 771-776.

Plath, T., Hocker, M., Riecken, E.O., Wang, T.C., Wiedenmann, B., and Rosewicz, S. (1999). Interferon-alpha inhibits chromogranin A promoter activity in neuroendocrine pancreatic cancer cells. FEBS Lett *458*, 378-382.

Powell, E., Kuhn, P., and Xu, W. (2007). Nuclear Receptor Cofactors in PPARgamma-Mediated Adipogenesis and Adipocyte Energy Metabolism. PPAR Res *2007*, 53843.

Preitner, N., Damiola, F., Lopez-Molina, L., Zakany, J., Duboule, D., Albrecht, U., and Schibler, U. (2002). The orphan nuclear receptor REV-ERBalpha controls circadian transcription within the positive limb of the mammalian circadian oscillator. Cell *110*, 251-260.

Purushotham, A., Schug, T.T., Xu, Q., Surapureddi, S., Guo, X., and Li, X. (2009). Hepatocyte-specific deletion of SIRT1 alters fatty acid metabolism and results in hepatic steatosis and inflammation. Cell Metab *9*, 327-338.

Rajamohan, S.B., Pillai, V.B., Gupta, M., Sundaresan, N.R., Birukov, K.G., Samant, S., Hottiger, M.O., and Gupta, M.P. (2009). SIRT1 promotes cell survival under stress by deacetylation-dependent deactivation of poly(ADP-ribose) polymerase 1. Mol Cell Biol *29*, 4116-4129.

Rana, S., and Mahmood, S. (2010). Circadian rhythm and its role in malignancy. J Circadian Rhythms *8*, 3.

Reymann, S., and Borlak, J. (2006). Transcriptome profiling of human hepatocytes treated with Aroclor 1254 reveals transcription factor regulatory networks and clusters of regulated genes. BMC Genomics *7*, 217.

Ripperger, J.A., and Schibler, U. (2006). Rhythmic CLOCK-BMAL1 binding to multiple E-box motifs drives circadian Dbp transcription and chromatin transitions. Nat Genet *38*, 369-374.

Robles, M.S., Boyault, C., Knutti, D., Padmanabhan, K., and Weitz, C.J. Identification of RACK1 and protein kinase Calpha as integral components of the mammalian circadian clock. Science *327*, 463-466.

Robles, M.S., Boyault, C., Knutti, D., Padmanabhan, K., and Weitz, C.J. (2010). Identification of RACK1 and protein kinase Calpha as integral components of the mammalian circadian clock. Science *327*, 463-466.

Rosenbaum, S.E., and Greenberg, A.S. (1998). The short- and long-term effects of tumor necrosis factor-alpha and BRL 49653 on peroxisome proliferator-activated receptor (PPAR)gamma2 gene expression and other adipocyte genes. Mol Endocrinol *12*, 1150-1160.

Sasaki, M., Yoshitane, H., Du, N.H., Okano, T., and Fukada, Y. (2009). Preferential inhibition of BMAL2-CLOCK activity by PER2 reemphasizes its negative role and a positive role of BMAL2 in the circadian transcription. J Biol Chem *284*, 25149-25159.

Schmutz, I., Ripperger, J.A., Baeriswyl-Aebischer, S., and Albrecht, U. (2010). The mammalian clock component PERIOD2 coordinates circadian output by interaction with nuclear receptors. Genes Dev *24*, 345-357.

Scoditti, E., Massaro, M., Carluccio, M.A., Distante, A., Storelli, C., and De Caterina, R. (2009). PPARgamma agonists inhibit angiogenesis by suppressing PKCalpha- and CREB-mediated COX-2 expression in the human endothelium. Cardiovasc Res *86*, 302-310.

Shaban, Z., El-Shazly, S., Abdelhady, S., Fattouh, I., Muzandu, K., Ishizuka, M., Kimura, K., Kazusaka, A., and Fujita, S. (2004). Down regulation of hepatic PPARalpha function by AhR ligand. J Vet Med Sci *66*, 1377-1386.

Shearman, L.P., Jin, X., Lee, C., Reppert, S.M., and Weaver, D.R. (2000). Targeted disruption of the mPer3 gene: subtle effects on circadian clock function. Mol Cell Biol *20*, 6269-6275.

Shi, S., Hida, A., McGuinness, O.P., Wasserman, D.H., Yamazaki, S., and Johnson, C.H. (2010). Circadian clock gene Bmal1 is not essential; functional replacement with its paralog, Bmal2. Curr Biol *20*, 316-321.

Solomou, E.E., Juang, Y.T., and Tsokos, G.C. (2001). Protein kinase C-theta participates in the activation of cyclic AMP-responsive element-binding protein and its subsequent binding to the -180 site of the IL-2 promoter in normal human T lymphocytes. J Immunol *166*, 5665-5674.

Spengler, M.L., Kuropatwinski, K.K., Schumer, M., and Antoch, M.P. (2009). A serine cluster mediates BMAL1-dependent CLOCK phosphorylation and degradation. Cell Cycle *8*, 4138-4146.

Stier, S., Totzke, G., Gruewald, E., Neuhaus, T., Fronhoffs, S., Schoneborn, S., Vetter, H., and Ko, Y. (2005). Identification of p54(nrb) and the 14-3-3 Protein HS1 as TNF-alpha-inducible genes related to cell cycle control and apoptosis in human arterial endothelial cells. J Biochem Mol Biol *38*, 447-456.

Stratmann, M., Stadler, F., Tamanini, F., van der Horst, G.T., and Ripperger, J.A. (2010). Flexible phase adjustment of circadian albumin D site-binding protein (DBP) gene expression by CRYPTOCHROME1. Genes Dev *24*, 1317-1328.

Sugawara, A., Takeuchi, K., Uruno, A., Kudo, M., Sato, K., and Ito, S. (2003). Effects of mitogen-activated protein kinase pathway and co-activator CREP-binding protein on peroxisome proliferator-activated receptor-gamma-mediated transcription suppression of angiotensin II type 1 receptor gene. Hypertens Res *26*, 623-628.

Szabo, G., Catalano, D., Bellerose, G., and Mandrekar, P. (2001). Interferon alpha and alcohol augment nuclear regulatory factor-kappaB activation in HepG2 cells, and interferon alpha increases pro-inflammatory cytokine production. Alcohol Clin Exp Res *25*, 1188-1197.

Takahashi, J.S., Hong, H.K., Ko, C.H., and McDearmon, E.L. (2008). The genetics of mammalian circadian order and disorder: implications for physiology and disease. Nat Rev Genet *9*, 764-775.

Teboul, M., Guillaumond, F., Grechez-Cassiau, A., and Delaunay, F. (2008). The nuclear hormone receptor family round the clock. Mol Endocrinol *22*, 2573-2582.

Tolon, R.M., Castillo, A.I., and Aranda, A. (1998). Activation of the prolactin gene by peroxisome proliferator-activated receptor-alpha appears to be DNA binding-independent. J Biol Chem *273*, 26652-26661.

Tordjman, J., Leroyer, S., Chauvet, G., Quette, J., Chauvet, C., Tomkiewicz, C., Chapron, C., Barouki, R., Forest, C., Aggerbeck, M., and Antoine, B. (2007). Cytosolic aspartate aminotransferase, a new partner in adipocyte glyceroneogenesis and an atypical target of thiazolidinedione. J Biol Chem *282*, 23591-23602.

Tyson, D.R., Swarthout, J.T., Jefcoat, S.C., and Partridge, N.C. (2002). PTH induction of transcriptional activity of the cAMP response element-binding protein requires the serine 129 site and glycogen synthase kinase-3 activity, but not casein kinase II sites. Endocrinology *143*, 674-682.

Ueda, H.R., Chen, W., Adachi, A., Wakamatsu, H., Hayashi, S., Takasugi, T., Nagano, M., Nakahama, K., Suzuki, Y., Sugano, S.*, et al.* (2002). A transcription factor response element for gene expression during circadian night. Nature *418*, 534-539.

Usacheva, A., Smith, R., Minshall, R., Baida, G., Seng, S., Croze, E., and Colamonici, O. (2001). The WD motif-containing protein receptor for activated protein kinase C (RACK1) is required for recruitment and activation of signal transducer and activator of transcription 1 through the type I interferon receptor. J Biol Chem *276*, 22948-22953.

Usacheva, A., Tian, X., Sandoval, R., Salvi, D., Levy, D., and Colamonici, O.R. (2003). The WD motif-containing protein RACK-1 functions as a scaffold protein within the type I IFN receptor-signaling complex. J Immunol *171*, 2989-2994.

Vendel, A.C., McBryant, S.J., and Lumb, K.J. (2003). KIX-mediated assembly of the CBP-CREB-HTLV-1 tax coactivator-activator complex. Biochemistry *42*, 12481-12487.

Villard, P.H., Caverni, S., Baanannou, A., Khalil, A., Martin, P.G., Penel, C., Pineau, T., Seree, E., and Barra, Y. (2007). PPARalpha transcriptionally induces AhR expression in Caco-2, but represses AhR pro-inflammatory effects. Biochem Biophys Res Commun *364*, 896-901.

von Knethen, A., Tzieply, N., Jennewein, C., and Brune, B. (2009). Casein-kinase-II-dependent phosphorylation of PPARgamma provokes CRM1-mediated shuttling of PPARgamma from the nucleus to the cytosol. J Cell Sci *123*, 192-201.

Wang, C., Fu, M., D'Amico, M., Albanese, C., Zhou, J.N., Brownlee, M., Lisanti, M.P., Chatterjee, V.K., Lazar, M.A., and Pestell, R.G. (2001). Inhibition of cellular proliferation through IkappaB kinase-independent and peroxisome proliferator-activated receptor gamma-dependent repression of cyclin D1. Mol Cell Biol *21*, 3057-3070.

Wang, N., Yang, G., Jia, Z., Zhang, H., Aoyagi, T., Soodvilai, S., Symons, J.D., Schnermann, J.B., Gonzalez, F.J., Litwin, S.E., and Yang, T. (2008). Vascular PPARgamma controls circadian variation in blood pressure and heart rate through Bmal1. Cell Metab *8*, 482-491.

Wisor, J.P., O'Hara, B.F., Terao, A., Selby, C.P., Kilduff, T.S., Sancar, A., Edgar, D.M., and Franken, P. (2002). A role for cryptochromes in sleep regulation. BMC Neurosci *3*, 20.

Xu, C.X., Krager, S.L., Liao, D.F., and Tischkau, S.A. (2010). Disruption of CLOCK-BMAL1 transcriptional activity is responsible for aryl hydrocarbon receptor-mediated regulation of Period1 gene. Toxicol Sci *115*, 98-108.

Yagita, K., Yamaguchi, S., Tamanini, F., van Der Horst, G.T., Hoeijmakers, J.H., Yasui, A., Loros, J.J., Dunlap, J.C., and Okamura, H. (2000). Dimerization and nuclear entry of mPER proteins in mammalian cells. Genes Dev *14*, 1353-1363.

Yang, J., Kim, K.D., Lucas, A., Drahos, K.E., Santos, C.S., Mury, S.P., Capelluto, D.G., and Finkielstein, C.V. (2008). A novel heme-regulatory motif mediates heme-dependent degradation of the circadian factor period 2. Mol Cell Biol *28*, 4697-4711.

Yin, L., Wu, N., and Lazar, M.A. (2010). Nuclear receptor Rev-erbalpha: a heme receptor that coordinates circadian rhythm and metabolism. Nucl Recept Signal *8*, e001.

Yoshizaki, T., Schenk, S., Imamura, T., Babendure, J.L., Sonoda, N., Bae, E.J., Oh, D.Y., Lu, M., Milne, J.C., Westphal, C.*, et al.* (2009). SIRT1 inhibits inflammatory pathways in macrophages and modulates insulin sensitivity. Am J Physiol Endocrinol Metab *298*, E419-428.

Yu, F., Chou, C.W., and Chen, C.C. (2009). TNF-alpha suppressed TGF-beta-induced CTGF expression by switching the binding preference of p300 from Smad4 to p65. Cell Signal *21*, 867-872.

Yuan, H.D., Yuan, H.Y., Chung, S.H., Jin, G.Z., and Piao, G.C. (2010). An Active Part of Artemisia sacrorum Ledeb. Attenuates Hepatic Lipid Accumulation through Activating AMP-Activated Protein Kinase in Human HepG2 Cells. Bioscience Biotechnology and Biochemistry *74*, 322-328.

Zerfaoui, M., Suzuki, Y., Naura, A.S., Hans, C.P., Nichols, C., and Boulares, A.H. (2008). Nuclear translocation of p65 NF-kappaB is sufficient for VCAM-1, but not ICAM-1, expression in TNF-stimulated smooth muscle cells: Differential requirement for PARP-1 expression and interaction. Cell Signal *20*, 186-194.

Zhang, E.E., and Kay, S.A. (2010). Clocks not winding down: unravelling circadian networks. Nat Rev Mol Cell Biol *11*, 764-776.

Zhang, H.N., Li, L., Gao, P., Chen, H.Z., Zhang, R., Wei, Y.S., Liu, D.P., and Liang, C.C. (2010). Involvement of the p65/RelA subunit of NF-kappaB in TNF-alpha-induced SIRT1 expression in vascular smooth muscle cells. Biochem Biophys Res Commun *397*, 569-575.

Zhou, L., Li, Y., Nie, T., Feng, S., Yuan, J., Chen, H., and Yang, Z. (2007). Clenbuterol inhibits SREBP-1c expression by activating CREB1. J Biochem Mol Biol *40*, 525-531.
