# Supplementary material for: Ras-Mediated Deregulation of the Circadian Clock in Cancer
Source: PLoS Genet. 2014 May 29;10(5):e1004338. doi: 10.1371/journal.pgen.1004338 (PMC4038477; doi:10.1371/journal.pgen.1004338)
Supplement: Text S3 — Analysis of circadian expression data for the genes of interest indicates enrichment in circadian genes. (DOC) [file pgen.1004338.s012.doc]

**Text S3 – Analysis of circadian expression data for the genes of interest indicates enrichment in circadian genes.**

To investigate possible circadian properties in the genes of interest indicated in Table 1, we collected several sets of gene expression data from distinct sources . These include time course expression data for human mouse and rat in a number of different tissues and cell lines, as represented in the schematic work flow in Figure 1.

The R programming language, version 2.12.0 for Windows 64bit is used for loading and processing the data, ID conversion, statistical testing and to ensure compatibility of platform and species, making the actual comparison and outputting the results . For importing the mRNA data into R we used GEOquery version 2.15.1. R is complemented by the BiomaRt (version 2.6.0) and org.Mm.eg.db (version 2.4.6.) packages. Both packages are developed for identifier conversion; BiomaRt also retrieves homologues and metadata of the genes that are being investigated from its web-service with the same name. Homologue identifier conversion is used to create a universally comparable dataset by finding homologue genes in human, mouse and rat genome. org.Mm.eg.db is used to complement BiomaRt for Entrez to gene symbol conversion. Each gene was assigned an Esembl ID, Gene Bank ID, gene name and gene symbol. Circadian expression was evaluated by statistical tests (Fisher’s exact test, Fisher’s G test). The overlap between our genes of interest and the different studies analysed is depicted in Tables 2-4. A final comparison listing all circadian genes-of-interest found is presented in Table 5.

| **Cancer-related genes/ID** | | | | **Core-clock genes/ID** | | **Discriminative genes/ID** | |
| --- | --- | --- | --- | --- | --- | --- | --- |
| **AKT (RAC)** | 207 | **PDK1** | 5163 | **AHR** | 196 | **APOBEC3F** | 200316 |
| **APC** | 324 | **PRKC-α** | 5578 | **ALAS1** | 211 | **AQR** | 9716 |
| **ATF-2 (CREB 2)** | 1386 | **PL3K** | 5290 | **AMPK** | 5562 | **ARGLU1** | 55082 |
| **Axin** | 8312 | **PLA2** | 5320 | **βTRCP** | 8945 | **ATP8B3** | 148229 |
| **BAD** | 572 | **PLA2G10** | 8399 | **BMAL1** | 406 | **CAPN6** | 827 |
| **BAX** | 581 | **PLC** | 5335 | **BMAL2** | 56938 | **CBX7** | 23492 |
| **β-catenin** | 1499 | **PLD** | 5337 | **CAR** | 9970 | **CHD4** | 1108 |
| **BRAF** | 673 | **RAF** | 673 | **CBP** | 1387 | **CHRNB4** | 1143 |
| **CD21** | 1380 | **RAL** | 5898 | **CLOCK** | 9575 | **CTCF** | 10664 |
| **CD24** | 960 | **RALGDS** | 5900 | **CREB** | 1385 | **CTSB** | 1508 |
| **CDC4** | 55294 | **RBP** | 5930 | **CRY1** | 1407 | **DTNB** | 1838 |
| **c-Fos** | 2353 | **Rho** | 29984 | **CRY2** | 1408 | **FBXO17** | 115290 |
| **c-Jun** | 3725 | **RON** | 7301 | **CSNK1** | 1453 | **FOXA1** | 3169 |
| **DPD (DPYD)** | 1806 | **RSK** | 6195 | **CSNK1ε** | 1454 | **GNG11** | 2791 |
| **DSH** | 8215 | **SFRP4** | 6424 | **CSNK2α** | 1457 | **GPRASP2** | 114928 |
| **DUSP16** | 80824 | **SHC** | 6464 | **DBP** | 1628 | **HNF4G** | 3174 |
| **ELK1** | 2002 | **SMAD4** | 4089 | **DEC1** | 8553 | **HYAL2** | 8692 |
| **EPHB2** | 2048 | **SOS** | 6654 | **DEC2** | 79365 | **IFNGR2** | 3460 |
| **ERCC1** | 2067 | **TCF4** | 6925 | **E4BP4** | 4783 | **INO80C** | 125476 |
| **ERCC2** | 2068 | **TGFBR2** | 7048 | **FBXL3** | 26224 | **KHDRBS3** | 10656 |
| **ERK** | 5595 | **TNFα** | 7124 | **GSK3β** | 2932 | **LOXL2** | 4017 |
| **FGFR1** | 2260 | **TNFR1** | 7133 | **HLF** | 3131 | **MAP1B** | 4131 |
| **FORKHEAD box M1** | 2305 | **TP53** | 7157 | **IFNα** | 3439 | **NINJ1** | 4814 |
| **FZD7** | 8324 | **TYMS** | 7298 | **NONO** | 4841 | **NUDT4P1** | 440672 |
| **GRB2** | 2885 | **UGT1A1** | 54658 | **NPAS2** | 4862 | **PDHX** | 8050 |
| **GSK3α** | 2931 | **VEGF** | 7422 | **p300** | 2033 | **PITX2** | 5308 |
| **GSK3β** | 2932 | **WNT** | 7471 | **PARP1** | 142 | **PPARδ** | 5467 |
| **GSTP1** | 2950 | **XRCC1** | 7515 | **PER1** | 5187 | **PPFIBP1** | 8496 |
| **HGF** | 3082 | **XRCC3** | 7517 | **PER2** | 8864 | **RAB6B** | 51560 |
| **HRAS** | 3265 |  |  | **PER3** | 8863 | **RBPMS** | 11030 |
| **IGF2R** | 3482 |  |  | **PPARα** | 5465 | **RFWD2** | 64326 |
| **JNKK** | 6416 |  |  | **PPARγ** | 5468 | **SCN5α** | 6331 |
| **KRAS** | 3845 |  |  | **PRKC-α** | 5578 | **SEC14L2** | 23541 |
| **MAP2K2** | 5605 |  |  | **RACK1** | 10399 | **SLC37A3** | 84255 |
| **MAPK1** | 5594 |  |  | **REV-ERBα** | 9572 | **SLC39A5** | 283375 |
| **MCC1** | 4163 |  |  | **REV-ERBβ** | 9975 | **SNORD58A** | 26791 |
| **MDM2** | 4193 |  |  | **RORα** | 6095 | **SNRPA** | 6626 |
| **MEK** | 5604 |  |  | **RORβ** | 6096 | **SPARC** | 6678 |
| **MEKK1** | 4214 |  |  | **RORγ** | 6097 | **ST3GAL5** | 8869 |
| **MET** | 4233 |  |  | **SIRT1** | 23411 | **STARD8** | 9754 |
| **MTHFR** | 4524 |  |  | **TEF** | 7008 | **TBCD** | 6904 |
| **NRAS** | 4893 |  |  | **TNFα** | 7124 | **TSPAN9** | 10867 |
| **p16** | 1029 |  |  | **WDR5** | 11091 | **USP22** | 23326 |
| **p70S6K** | 6198 |  |  |  |  | **WASF3** | 10810 |
| **PAK** | 5058 |  |  |  |  | **XYLT2** | 64132 |

**Table1.** Genes of interest grouped into three categories: genes found to be associated to carcinogenesis (cancer-related genes), core-clock genes and a novel set of genes retrieved from the microarray analysis and able to discriminate strong and weak oscillators among the analyzed cell lines (discriminative genes). Human NCBI entrez gene-IDs are indicated.

##

**Figure 1.** Methodological work-flow. The time-course gene expression data was collected from three studies . A total of 14 different tissues and three different cell lines including Human, mouse and rat as model organism, were screened for circadian gene expression profiles. The intersection between the lists of genes found and the genes of interest (Table 1) was determined. As a result a subset of circadian expressed genes was obtained and listed in Table 5.

| **Cancer-related genes** | | | **Core-clock genes** | | | **Discriminative genes** | | | |
| --- | --- | --- | --- | --- | --- | --- | --- | --- | --- |
| **Name_H** | **ID_H** | **ID_M** | **Name_H** | **ID_H** | **ID_M** | | **Name_H** | **ID_H** | **ID_M** |
| **ATF2** | 1386 | 11909 | **ARNTL** | 406 | 11865 | | **GNG11** | 2791 | 66066 |
| **ATF2** | 1386 | 100047997 | **BHLHE40** | 8553 | 20893 | | **HYAL2** | 8692 | 15587 |
| **DUSP16** | 80824 | 70686 | **CLOCK** | 9575 | 12753 | | **NINJ1** | 4814 | 18081 |
| **FBXW7** | 55294 | 50754 | **CREBBP** | 1387 | 12914 | | **PDHX** | 8050 | 27402 |
| **GSK3A** | 2931 | 606496 | **CRY1** | 1407 | 12952 | | **PPFIBP1** | 8496 | 67533 |
| **HRAS** | 3265 | 15461 | **CSNK2A1** | 1457 | 12995 | | **RBPMS** | 11030 | 19663 |
| **IGF2R** | 3482 | 16004 | **DBP** | 1628 | 13170 | | **SPARC** | 6678 | 20692 |
| **MAP2K4** | 6416 | 26398 | **FBXL3** | 26224 | 50789 | | **ST3GAL5** | 8869 | 20454 |
| **MAP3K1** | 4214 | 26401 | **HLF** | 3131 | 217082 | | **TSPAN9** | 10867 | 109246 |
| **MCC** | 4163 | 328949 | **NFIL3** | 4783 | 18030 | |  |  |  |
| **MET** | 4233 | 17295 | **NONO** | 4841 | 53610 | |  |  |  |
| **MTHFR** | 4524 | 17769 | **NPAS2** | 4862 | 18143 | |  |  |  |
| **NRAS** | 4893 | 18176 | **NR1D1** | 9572 | 217166 | |  |  |  |
| **PDK1** | 5163 | 228026 | **NR1D2** | 9975 | 353187 | |  |  |  |
| **PIK3CA** | 5290 | 18706 | **PER1** | 5187 | 18626 | |  |  |  |
| **PLCG1** | 5335 | 18803 | **PER2** | 8864 | 18627 | |  |  |  |
| **PLD1** | 5337 | 18805 | **PER3** | 8863 | 18628 | |  |  |  |
| **RBBP6** | 5930 | 19647 | **PPARA** | 5465 | 19013 | |  |  |  |
| **RHOD** | 29984 | 11854 | **RORA** | 6095 | 19883 | |  |  |  |
| **SMAD4** | 4089 | 17128 | **RORC** | 6097 | 19885 | |  |  |  |
|  |  |  | **TEF** | 7008 | 21685 | |  |  |  |

**Table2** - Genes of interested found to be oscillating in Hughes *et al*. gene expression data set.

| **Cancer-related genes** | | | **Core-clock genes** | | | **Discriminative genes** | | |
| --- | --- | --- | --- | --- | --- | --- | --- | --- |
| **Name_H** | **ID_H** | **ID_M** | **Name_H** | **ID_H** | **ID_M** | **Name_H** | **ID_H** | **ID_M** |
| **DUSP16** | 80824 | 70686 | **AHR** | 196 | 11622 | **KHDRBS3** | 10656 | 13992 |
| **FZD7** | 8324 | 14369 | **CLOCK** | 9575 | 12753 | **NINJ1** | 4814 | 18081 |
| **GSTP1** | 2950 | 14870 | **CRY2** | 1408 | 12953 | **SEC14L2** | 23541 | 67815 |
| **MAP3K1** | 4214 | 26401 | **DBP** | 1628 | 13170 |  |  |  |
| **MTHFR** | 4524 | 17769 | **NFIL3** | 4783 | 18030 |  |  |  |
| **PRKCA** | 5578 | 18750 | **PER2** | 8864 | 18627 |  |  |  |
| **RBBP6** | 5930 | 19647 | **PPARA** | 5465 | 19013 |  |  |  |
| **RHOD** | 29984 | 11854 | **RORA** | 6095 | 19883 |  |  |  |
| **SFRP4** | 6424 | 20379 | **RORC** | 6097 | 19885 |  |  |  |
| **SMAD4** | 4089 | 17128 | **BHLHE40** | 8553 | 20893 |  |  |  |
| **TNFRSF1B** | 7133 | 21938 | **TEF** | 7008 | 21685 |  |  |  |
|  |  |  | **NONO** | 4841 | 53610 |  |  |  |
|  |  |  | **HLF** | 3131 | 217082 |  |  |  |
|  |  |  | **NR1D1** | 9572 | 217166 |  |  |  |
|  |  |  | **NR1D2** | 9975 | 353187 |  |  |  |

**Table3** - Genes of interested found to be oscillating in Bozek *et al*. gene expression data set.

| **Cancer-related genes** | | | **Core-clock genes** | | | **Discriminative genes** | | |
| --- | --- | --- | --- | --- | --- | --- | --- | --- |
| **Name_H** | **ID_H** | **ID_M** | **Name_H** | **ID_H** | **ID_M** | **Name_H** | **ID_H** | **ID_M** |
| **AKT1** | 207 | 11651 | **AHR** | 196 | 11622 |  |  |  |
| **ATF2** | 1386 | 11909 | **ARNTL** | 406 | 11865 |  |  |  |
| **AXIN1** | 8312 | 12005 | **BHLHE40** | 8553 | 20893 |  |  |  |
| **BAD** | 572 | 12015 | **BTRC** | 8945 | 12234 |  |  |  |
| **CD44** | 960 | 12505 | **CLOCK** | 9575 | 12753 |  |  |  |
| **CR2** | 1380 | 12902 | **CREB1** | 1385 | 12912 |  |  |  |
| **CTNNB1** | 1499 | 12387 | **CRY1** | 1407 | 12952 |  |  |  |
| **DPYD** | 1806 | 99586 | **CRY2** | 1408 | 12953 |  |  |  |
| **ELK1** | 2002 | 13712 | **CSNK1D** | 1453 | 104318 |  |  |  |
| **ERCC1** | 2067 | 13870 | **CSNK1E** | 1454 | 27373 |  |  |  |
| **ERCC2** | 2068 | 13871 | **CSNK2A1** | 1457 | 12995 |  |  |  |
| **FBXW7** | 55294 | 50754 | **DBP** | 1628 | 13170 |  |  |  |
| **FGFR1** | 2260 | 14182 | **FBXL3** | 26224 | 50789 |  |  |  |
| **FOS** | 2353 | 14281 | **GNB2L1** | 10399 | 14694 |  |  |  |
| **FOXM1** | 2305 | 14235 | **GSK3B** | 2932 | 56637 |  |  |  |
| **FZD7** | 8324 | 14369 | **HLF** | 3131 | 217082 |  |  |  |
| **GRB2** | 2885 | 14784 | **NFIL3** | 4783 | 18030 |  |  |  |
| **GSK3A** | 2931 | 606496 | **NONO** | 4841 | 53610 |  |  |  |
| **GSK3B** | 2932 | 56637 | **NPAS2** | 4862 | 18143 |  |  |  |
| **HRAS** | 3265 | 15461 | **NR1D1** | 9572 | 217166 |  |  |  |
| **IGF2R** | 3482 | 16004 | **NR1D2** | 9975 | 353187 |  |  |  |
| **JUN** | 3725 | 16476 | **PARP1** | 142 | 11545 |  |  |  |
| **MAP2K1** | 5604 | 26395 | **PER1** | 5187 | 18626 |  |  |  |
| **MTHFR** | 4524 | 17769 | **PER2** | 8864 | 18627 |  |  |  |
| **NRAS** | 4893 | 18176 | **PER3** | 8863 | 18628 |  |  |  |
| **PAK1** | 5058 | 18479 | **PPARA** | 5465 | 19013 |  |  |  |
| **PIK3CA** | 5290 | 18706 | **RORA** | 6095 | 19883 |  |  |  |
| **PLA2G10** | 8399 | 26565 | **RORB** | 6096 | 225998 |  |  |  |
| **PLD1** | 5337 | 18805 | **RORC** | 6097 | 19885 |  |  |  |
| **PRKCA** | 5578 | 18750 | **TEF** | 7008 | 21685 |  |  |  |
| **RALA** | 5898 | 56044 | **WDR5** | 11091 | 140858 |  |  |  |
| **RBBP6** | 5930 | 19647 |  |  |  |  |  |  |
| **RHOD** | 29984 | 11854 |  |  |  |  |  |  |
| **RPS6KA1** | 6195 | 20111 |  |  |  |  |  |  |
| **RPS6KB1** | 6198 | 72508 |  |  |  |  |  |  |
| **SHC1** | 6464 | 20416 |  |  |  |  |  |  |
| **SOS1** | 6654 | 20662 |  |  |  |  |  |  |
| **TGFBR2** | 7048 | 21813 |  |  |  |  |  |  |
| **TP53** | 7157 | 22059 |  |  |  |  |  |  |
| **TYMS** | 7298 | 22171 |  |  |  |  |  |  |
| **TYRO3** | 7301 | 22174 |  |  |  |  |  |  |
| **VEGFA** | 7422 | 22339 |  |  |  |  |  |  |

**Table 4.** - Genes of interested found to be oscillating in Yan *et al* gene expression data set.

| **circadian**  **(33/43)** | **ID** | **Cancer**  **(55/74)** | **ID** | **Cancer**  **(55/74)** | **ID** | **Discriminative**  **(11/45)** | **ID** |
| --- | --- | --- | --- | --- | --- | --- | --- |
| p-value = 2.6517e-26 | | p-value = 1.0249e-41 | | | | p-value = 0.0019 | |
| **AHR** | 196 | **AKT1** | 207 | **PDK1** | 5163 | **GNG11** | *2791* |
| **ARNTL** | 406 | **ATF2** | 1386 | **PIK3CA** | 5290 | **HYAL2** | *8692* |
| **BHLHE40** | 8553 | **AXIN1** | 8312 | **PLA2G10** | 8399 | **KHDRBS3** | *10656* |
| **BTRC** | 8945 | **BAD** | 572 | **PLCG1** | 5335 | **NINJ1** | *4814* |
| **CLOCK** | 9575 | **CD44** | 960 | **PLD1** | 5337 | **PDHX** | *8050* |
| **CREB1** | 1385 | **CR2** | 1380 | **PRKCA** | 5578 | **PPFIBP1** | *8496* |
| **CREBBP** | 1387 | **CTNNB1** | 1499 | **RALA** | 5898 | **RBPMS** | *11030* |
| **CRY1** | 1407 | **DPYD** | 1806 | **RALGDS** | 5900 | **SEC14L2** | *23541* |
| **CRY2** | 1408 | **DUSP16** | 80824 | **RBBP6** | 5930 | **SPARC** | *6678* |
| **CSNK1D** | 1453 | **ELK1** | 2002 | **RHOD** | 29984 | **ST3GAL5** | *8869* |
| **CSNK1E** | 1454 | **ERCC1** | 2067 | **RPS6KA1** | 6195 | **TSPAN9** | *10867* |
| **CSNK2A1** | 1457 | **ERCC2** | 2068 | **RPS6KB1** | 6198 |  |  |
| **DBP** | 1628 | **FBXW7** | 55294 | **SFRP4** | 6424 |  |  |
| **FBXL3** | 26224 | **FGFR1** | 2260 | **SHC1** | 6464 |  |  |
| **GNB2L1** | 10399 | **FOS** | 2353 | **SMAD4** | 4089 |  |  |
| **GSK3B** | 2932 | **FOXM1** | 2305 | **SOS1** | 6654 |  |  |
| **HLF** | 3131 | **FZD7** | 8324 | **TGFBR2** | 7048 |  |  |
| **NFIL3** | 4783 | **GRB2** | 2885 | **TNFRSF1B** | 7133 |  |  |
| **NONO** | 4841 | **GSK3A** | 2931 | **TP53** | 7157 |  |  |
| **NPAS2** | 4862 | **GSK3B** | 2932 | **TYMS** | 7298 |  |  |
| **NR1D1** | 9572 | **GSTP1** | 2950 | **TYRO3** | 7301 |  |  |
| **NR1D2** | 9975 | **HRAS** | 3265 | **VEGFA** | 7422 |  |  |
| **PARP1** | 142 | **IGF2R** | 3482 |  |  |  |  |
| **PER1** | 5187 | **JUN** | 3725 |  |  |  |  |
| **PER2** | 8864 | **MAP2K1** | 5604 |  |  |  |  |
| **PER3** | 8863 | **MAP2K4** | 6416 |  |  |  |  |
| **PPARA** | 5465 | **MAP3K1** | 4214 |  |  |  |  |
| **RORA** | 6095 | **MCC** | 4163 |  |  |  |  |
| **RORB** | 6096 | **MET** | 4233 |  |  |  |  |
| **RORC** | 6097 | **MTHFR** | 4524 |  |  |  |  |
| **TEF** | 7008 | **NRAS** | 4893 |  |  |  |  |
| **WDR5** | 11091 | **PAK1** | 5058 |  |  |  |  |

**Table 5.** Genes of interest found to display a circadian gene expression profile in at least one of the studies analyzed (p-values determined with Fisher’s test).

**References**

Bozek, K., Relogio, A., Kielbasa, S.M., Heine, M., Dame, C., Kramer, A., and Herzel, H. (2009). Regulation of clock-controlled genes in mammals. PLoS One *4*, e4882.

Hughes, M.E., DiTacchio, L., Hayes, K.R., Vollmers, C., Pulivarthy, S., Baggs, J.E., Panda, S., and Hogenesch, J.B. (2009). Harmonics of circadian gene transcription in mammals. PLoS Genet *5*, e1000442.

R. Ihaka, R.G. (1995). The R Statistical Programming Language.

Yan, J., Wang, H., Liu, Y., and Shao, C. (2008). Analysis of gene regulatory networks in the mammalian circadian rhythm. PLoS computational biology *4*, e1000193.
