# Supplementary material for: Ras-Mediated Deregulation of the Circadian Clock in Cancer
Source: PLoS Genet. 2014 May 29;10(5):e1004338. doi: 10.1371/journal.pgen.1004338 (PMC4038477; doi:10.1371/journal.pgen.1004338)
Supplement: Text S4 — Statistical significance of the 45-discriminative-gene list. (DOC) [file pgen.1004338.s013.doc]

**Text S4 – Statistical significance of the 45-discriminative-gene list**

**Quality control of microarrays**

A quality control procedure was carried out in all arrays, following the same method as before (Text S1). Additionally, we re-normalized all arrays using -frozen robust multiarray analysis (frma)- which has better characteristics when working incrementally with new batches of arrays . For array quality control we used the bioconductor package arrayQualityMetrics . A total of six quality tests were performed (Figures 1 – 12). The outlier detection criteria are explained below in the respective sections. Arrays that were called outliers by at least one criterion are marked in Table 1, where the overall performance of the arrays is listed.

| array | [*1](http://www2.informatik.hu-berlin.de/~thomas/qcFinal/#hm) | [*2](http://www2.informatik.hu-berlin.de/~thomas/qcFinal/#box) | [*3](http://www2.informatik.hu-berlin.de/~thomas/qcFinal/#rle) | [*4](http://www2.informatik.hu-berlin.de/~thomas/qcFinal/#nuse) | [*5](http://www2.informatik.hu-berlin.de/~thomas/qcFinal/#ma) | [*6](http://www2.informatik.hu-berlin.de/~thomas/qcFinal/#spm) | Cell line | Time point  [hours] |
| --- | --- | --- | --- | --- | --- | --- | --- | --- |
| 1 | x |  |  | x | x |  | U2OS | 0 |
| 2 |  |  |  |  | x | x | HCT116 | 0 |
| 3 |  |  |  |  | x |  | RKO | 0 |
| 4 |  |  |  |  | x |  | RKO | 48 |
| 5 |  |  |  |  | x |  | Caco | 0 |
| 6 |  |  |  |  | x |  | Caco | 48 |
| 7 |  |  |  |  |  |  | HCT116 | 48 |
| 8 |  |  |  |  |  |  | LIM1215 | 0 |
| 9 |  |  |  |  | x |  | LIM1215 | 48 |
| 10 |  |  |  |  | x |  | HT29 | 0 |
| 11 |  |  |  |  |  |  | HT29 | 48 |
| 12 |  |  |  |  |  | x | SW480 | 0 |
| 13 |  |  |  |  |  | x | SW480 | 48 |
| 14 |  |  |  |  | x |  | SW403 | 0 |
| 15 | x |  |  |  | x |  | SW403 | 48 |
| 16 |  |  |  |  | x |  | COLO205 | 0 |
| 17 |  |  |  |  | x |  | COLO205 | 48 |
| 18 |  |  |  |  |  |  | SW620 | 0 |
| 19 |  |  |  |  | x |  | SW620 | 48 |
| 20 |  |  |  |  |  |  | CaCo2 | 0 |
| 21 |  |  |  |  |  |  | CaCo2 | 48 |
| 22 |  |  |  |  |  |  | A5RT3 | 0 |
| 23 |  |  |  |  |  |  | A5RT3 | 48 |
| 24 |  |  |  |  | x |  | HaCat | 0 |
| 25 |  |  |  |  | x |  | HaCat | 48 |
| 26 |  |  |  |  | x |  | HKE3 | 0 |
| 27 |  |  |  |  | x |  | HKE3 | 48 |
| 28 |  |  |  |  | x |  | HKE3ind | 48 |
| 29 |  |  |  |  |  |  | Klon8 | 0 |
| 30 |  |  |  |  | x |  | Klon8 | 48 |
| 31 |  |  |  |  | x |  | Klon8ind | 48 |
| 32 |  |  | x |  |  |  | HCT116 | 0 |
| 33 |  |  |  |  |  |  | HCT116 | 48 |

[**Table1. Array metadata and outlier detection overview**](javascript: toggle('arraymetadata'))**.** The columns with * indicate the calls from the different outlier detection methods: 1 - outlier detection by Distances between arrays; 2 - outlier detection by [Boxplots](http://www2.informatik.hu-berlin.de/~thomas/qcFinal/#box); 3 - outlier detection by Relative Log Expression (RLE); 4 - outlier detection by Normalized Unscaled Standard Error (NUSE); 5 - outlier detection by MA plots; 6 - outlier detection by Spatial distribution of M.

## 1 – Outlier detection by distance between arrays


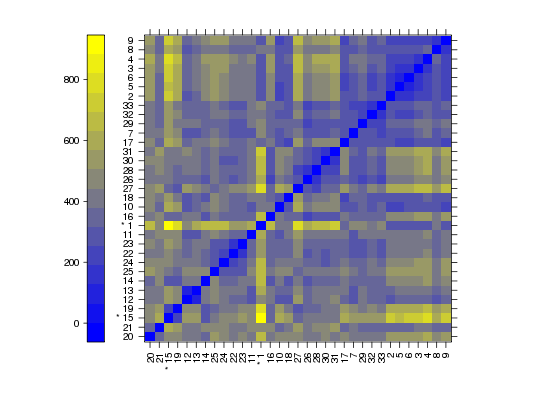


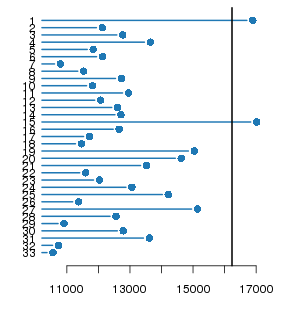
**Figure 1.** False colour heatmap of the distances between arrays. The colour scale is chosen to cover the range of distances encountered in the dataset. Patterns in this plot can indicate clustering of the arrays either because of intended biological or unintended experimental factors (batch effects). The distance *d_ab_* between two arrays *a* and *b* is computed as the mean absolute difference (L_1_-distance) between the data of the arrays (using the data from all probes without filtering). In formula, *d_ab_* = mean | *M_ai_ - M_bi_* |, where *M_ai_* is the value of the *i*-th probe on the *a*-th array. Outlier detection was performed by looking for arrays for which the sum of the distances to all other arrays, *S_a_* = Σ*_b_* *d_ab_* was exceptionally large. 2 such arrays were detected, and they are marked by an asterisk, *.

**Figure 2.** Bar chart of the sum of distances to other arrays Sa, the outlier detection criterion from the previous figure. The bars are shown in the original order of the arrays. Based on the distribution of the values across all arrays, a threshold of 4870 was determined, which is indicated by the vertical line. 2 arrays exceeded the threshold and were considered outliers.

**2 - Outlier detection by [Boxplots](http://www2.informatik.hu-berlin.de/~thomas/qcFinal/#box)**


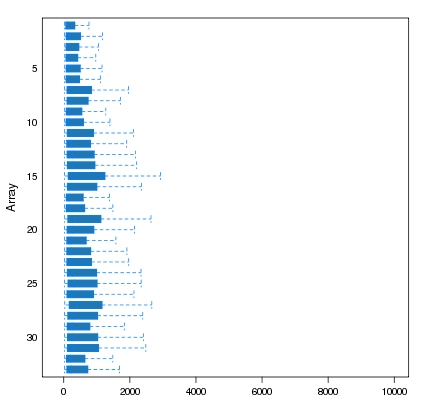


**Figure 3.** Boxplots representing summaries of the signal intensity distributions of the arrays. Each box corresponds to one array. Typically, it is expected that the boxes have similar positions and widths. If the distribution of an array is very different from the others, this may indicate an experimental problem. Outlier detection was performed by computing the Kolmogorov-Smirnov statistic *K_a_* between each array's distribution and the distribution of the pooled data.


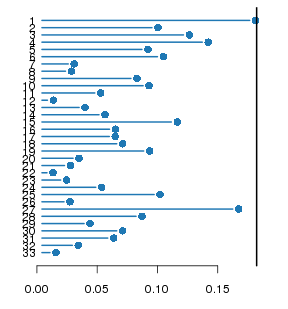


**Figure 4**. Bar chart of the Kolmogorov-Smirnov statistic *K_a_*, the outlier detection criterion from the previous figure. The bars are shown in the original order of the arrays. Based on the distribution of the values across all arrays, a threshold of 0.182 was determined, which is indicated by the vertical line. None of the arrays exceeded the threshold and was considered an outlier.

**3 - Outlier detection by Relative Log Expression (RLE)**


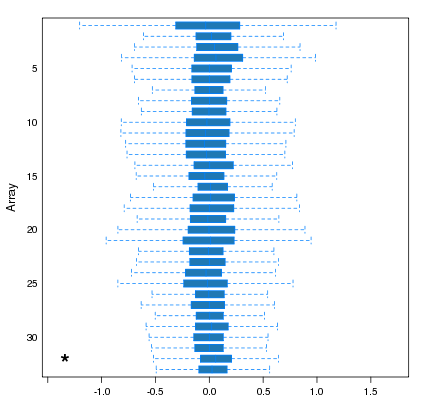


**Figure 5.** *Relative Log Expression (RLE)* plot. Arrays whose boxes are centered away from 0 and/or are more spread out are potentially problematic. Outlier detection was performed by computing the Kolmogorov-Smirnov statistic *R_a_* between each array's RLE values and the pooled, overall distribution of RLE values.


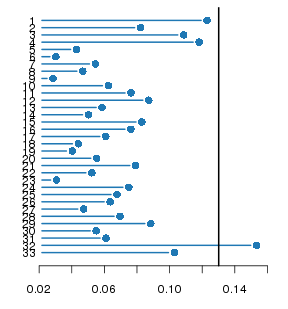


**Figure 6.** Bar chart of the Kolmogorov-Smirnov statistic Ra of the RLE values, the outlier detection criterion from the previous figure. The bars are shown in the original order of the arrays. Based on the distribution of the values across all arrays, a threshold of 0.13 was determined, which is indicated by the vertical line. One array exceeded the threshold.

**4 - Outlier detection by Normalized Unscaled Standard Error (NUSE)**


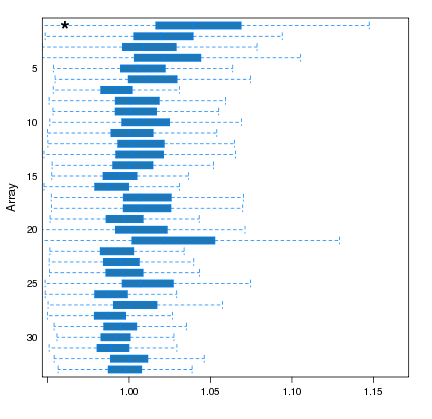


**Figure 7**. *Normalized Unscaled Standard Error (NUSE)* plot. For each array, the boxes should be centered around 1. An array were the values are elevated relative to the other arrays is typically of lower quality. Outlier detection was performed by computing the 75% quantile *N_a_* of each array's NUSE values and looking for arrays with large *N_a_*.

**
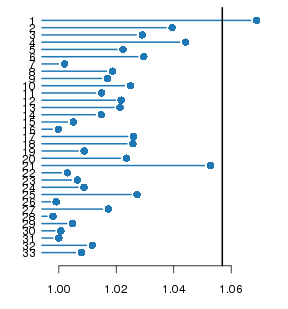
**

**Figure 8.** Bar chart of the *N_a_*, the outlier detection criterion from the previous figure. The bars are shown in the original order of the arrays. Based on the distribution of the values across all arrays, a threshold of 1.05 was determined, which is indicated by the vertical line. One array exceeded the threshold.

**5 - Outlier detection by MA plots**


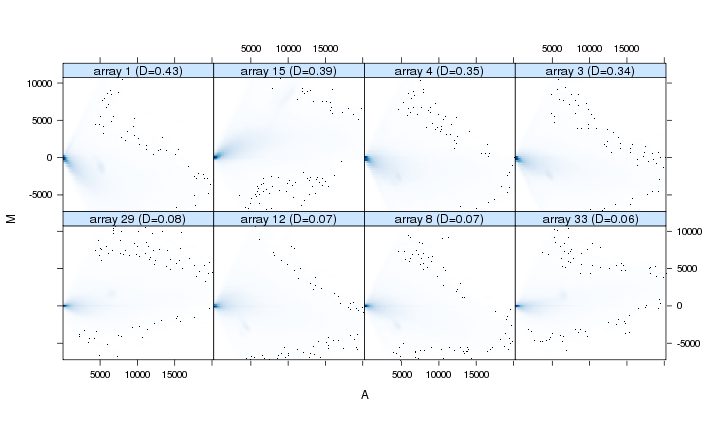


**Figure 9**. MA plots. M and A are defined as: M = log_2_(I_1_) - log_2_(I_2_) A = 1/2 (log_2_(I_1_)+log_2_(I_2_)), where I_1_ is the intensity of the array studied, and I_2_ is the intensity of a "pseudo"-array that consists of the median across arrays. Typically, we expect the mass of the distribution in an MA plot to be concentrated along the M = 0 axis, and there should be no trend in M as a function of A. If there is a trend in the lower range of A, this often indicates that the arrays have different background intensities; this may be addressed by background correction. A trend in the upper range of A can indicate saturation of the measurements; in mild cases, this may be addressed by non-linear normalisation (e.g. quantile normalisation). Outlier detection was performed by computing Hoeffding's statistic *D_a_* on the joint distribution of A and M for each array. Shown are the 4 arrays with the highest value of *D_a_* (top row), and the 4 arrays with the lowest value (bottom row). The value of *D_a_* is shown in the panel headings. 20 arrays had *D_a_*>0.15 and were marked as outliers. For more information on Hoeffing's *D*-statistic, please see the manual page of the function hoeffd in the Hmisc package.

**
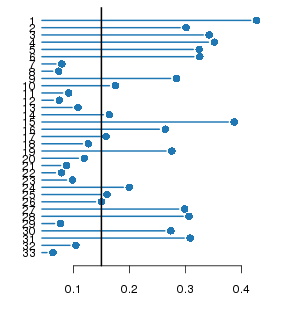
**

**Figure 10.** Bar chart of the Hoeffding's statistic *D_a_*, the outlier detection criterion from the previous figure. The bars are shown in the original order of the arrays. A threshold of 0.15 was used, which is indicated by the vertical line. 6 arrays exceeded the threshold.

**6 - Outlier detection by Spatial distribution of M.**


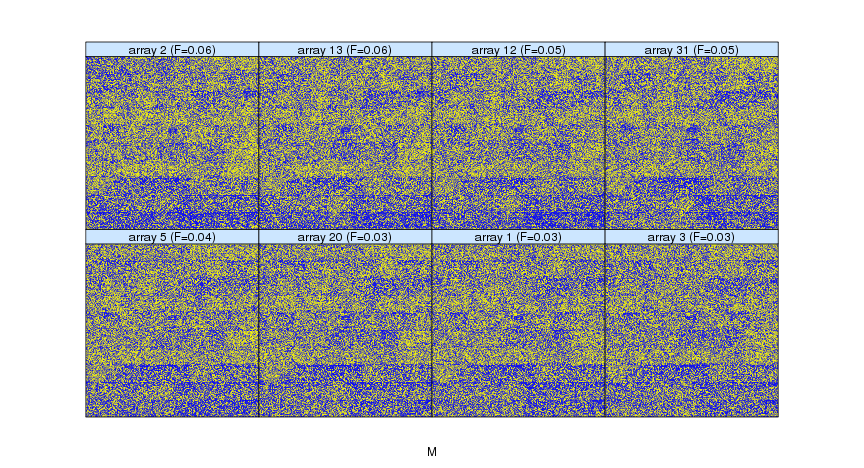


**Figure 11**. False colour representations of the arrays' spatial distributions of feature intensities (M). When the features are distributed randomly on the arrays, a uniform distribution is expected; control features with particularly high or low intensities stand out. The colour scale is proportional to the ranks of the probe intensities. The rank scale has the potential to amplify patterns that are small in amplitude but systematic within an array. It is possible to switch off the rank scaling by modifying the argument scale in the call of the aqm.spatial function. Outlier detection was performed by computing *F_a_* , the sum of the absolutes value of low frequency Fourier coefficients, as a measure of large scale spatial structures. Shown are the 4 arrays with the highest value of *S* (top row), and the 4 arrays with the lowest value (bottom row). The value of *F_a_* is shown in the panel headings.


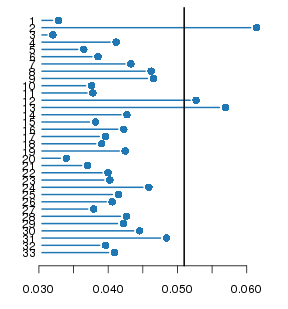


**Figure 12.** Bar chart of the *F_a_*, the outlier detection criterion from the previous figure. The bars are shown in the original order of the arrays. Based on the distribution of the values across all arrays, a threshold of 0.051 was determined, which is indicated by the vertical line. 3 arrays exceeded the threshold and were considered outliers.

**Statistical significance of the 45-discriminative-gene list**

The set of 45 genes obtained (Supplement 1) allowed the discrimination at the gene expression level between weak and strong oscillator. We now set to retrieve the discriminative power of our list of 45 genes, regarding the oscillator strength (strong vs week oscillator) as previously defined. We generated additional bioluminescence data and array data for several cell lines: a) three colon cancer cell lines (SW403, Colo205, SW620), two time points; b) two additional cell lines, derived from HCT116 (colon cancer) after genetic removal of oncogenic KRAS (Hke3) and a derivative cell line harbouring a conditional KRAS oncogene (Hek-klone8-ind) one time point, c) two keratinocytes cell lines (HaCat and A5RT3). Bioluminescence measurements (Figures S51, S6, S7) were carried out as previously described (Materials and Methods).

To test the robustness of our system, we repeated the arrays for HCT116 as a control for a strong oscillator and CaCo as a control for a week oscillator. The new replicate arrays behave as the previous ones and allow the reproduction of the data (Figure1). All arrays were normalized together, to allow a better comparison of the data. For the subsequent heat maps we use the new replicates to minimize potential batch problems within the previous and the new arrays. In total we evaluated the discriminative power of the 45 gene list for 8 cell lines, from which 7 were correctly classified and one was not correctly classified (Hke-Klone8 ind). Although this cell line is a week oscillator, with a much larger period and smaller amplitude than good oscillator cell lines it clusters with the HCT116 cell line (from which it was derived).

**Figure 13.** The U2OS (strong oscillator) cell line was used to test the discriminative power of the discriminative list. The Pearson distance function and ward clustering were used. Colour bar on the right corners of the pictures indicates the expression levels for genes in the array, from green (low expressed) to red (high expressed). Colour bar on top of the heatmap indicates class membership. Blue indicates strong oscillator, red indicates weak oscillator, and green indicates reference sample (U2OS).

**Figure 14.** The performance of the list for the cell line Colo205 (weak oscillator) was tested by clustering the 14 arrays and the corresponding heatmap is depicted in the figure. Pearson distance function and ward clustering were used. Colour bar on the right corners of the pictures indicates the expression levels for genes in the array, from green (low expressed) to red (high expressed). Colour bar on top of the heatmap indicates class membership. Blue indicates strong oscillator, red indicates weak oscillator.

**Figure 15.** The performance of the list for the cell line SW403 (weak oscillator) was tested by clustering the 14 arrays and the corresponding heatmap is depicted in the figure. Pearson distance function and ward clustering were used. Colour bar on the right corners of the pictures indicates the expression levels for genes in the array, from green (low expressed) to red (high expressed). Colour bar on top of the heatmap indicates class membership. Blue indicates strong oscillator, red indicates weak oscillator.

**Figure 16.** The performance of the list for the cell line SW620 (weak oscillator) was tested by clustering the 14 arrays and the corresponding heatmap is depicted in the figure. Pearson distance function and ward clustering were used. Colour bar on the right corners of the pictures indicates the expression levels for genes in the array, from green (low expressed) to red (high expressed). Colour bar on top of the heatmap indicates class membership. Blue indicates strong oscillator, red indicates weak oscillator.

**Figure 17.** The performance of the list for the cell line Hke3 (strong oscillator) and the control (Hke induced with mefiprestone) was tested by clustering the 15 arrays. The corresponding heatmap is depicted in the figure. Pearson distance function and ward clustering were used. Colour bar on the right corners of the pictures indicates the expression levels for genes in the array, from green (low expressed) to red (high expressed). Colour bar on top of the heatmap indicates class membership. Blue indicates strong oscillator, red indicates weak oscillator.

**Figure 18.** The performance of the list for the cell line Klone8 induced (weak oscillator) was tested by clustering the 13 arrays and the corresponding heatmap is depicted in the figure.

Pearson distance function and ward clustering were used. Colour bar on the right corners of the pictures indicates the expression levels for genes in the array, from green (low expressed) to red (high expressed). Colour bar on top of the heatmap indicates class membership. Blue indicates strong oscillator, red indicates weak oscillator.

**Figure 19.** The performance of the list for the cell line HaCat (strong oscillator) was tested by clustering the 14 arrays and the corresponding heatmap is depicted in the figure. Pearson distance function and ward clustering were used. Colour bar on the right corners of the pictures indicates the expression levels for genes in the array, from green (low expressed) to red (high expressed). Colour bar on top of the heatmap indicates class membership. Blue indicates strong oscillator, red indicates weak oscillator.

**Figure 20.** The performance of the list for the cell line A5RT3 (weak oscillator) was tested by clustering the 14 arrays and the corresponding heatmap is depicted in the figure. Pearson distance function and ward clustering were used. Colour bar on the right corners of the pictures indicates the expression levels for genes in the array, from green (low expressed) to red (high expressed). Colour bar on top of the heatmap indicates class membership. Blue indicates strong oscillator, red indicates weak oscillator.

The predefined list of 45 discriminative genes could be used to correctly classify seven out of eight cell-lines. Using binomial test we calculated the probability of observing the same or better classification result by random. According to this experiment our classifier performs significantly better than a random classifier (p=0.03516).

**References**

1. McCall, M.N., H.A. Jaffee, and R.A. Irizarry, *fRMA ST: frozen robust multiarray analysis for Affymetrix Exon and Gene ST arrays.* Bioinformatics, 2012. **28**(23): p. 3153-4.

2. Kauffmann, A., R. Gentleman, and W. Huber, *arrayQualityMetrics--a bioconductor package for quality assessment of microarray data.* Bioinformatics, 2009. **25**(3): p. 415-6.
